# *In-silico* analysis of heat shock transcription factor (*OsHSF*) gene family in rice (*Oryza sativa* L.)

**DOI:** 10.1186/s12870-023-04399-1

**Published:** 2023-08-17

**Authors:** Areeqa Shamshad, Muhammad Rashid, Qamar uz Zaman

**Affiliations:** 1 Nuclear Institute for Agriculture and Biology College, Pakistan Institute of Engineering and Applied Sciences (NIAB-C, PIEAS), Faisalabad, Pakistan; 2 Department of Environmental Sciences, The University of Lahore, Lahore, 54590 Pakistan

**Keywords:** *Oryza sativa* L, Heat shock factor gene family, Phylogeny, Chromosomal localization, Gene networking and expression analysis

## Abstract

**Background:**

One of the most important cash crops worldwide is rice (*Oryza sativa* L.). Under varying climatic conditions, however, its yield is negatively affected. In order to create rice varieties that are resilient to abiotic stress, it is essential to explore the factors that control rice growth, development, and are source of resistance. HSFs (heat shock transcription factors) control a variety of plant biological processes and responses to environmental stress. The *in-silico* analysis offers a platform for thorough genome-wide identification of *OsHSF* genes in the rice genome.

**Results:**

In this study, 25 randomly dispersed HSF genes with significant DNA binding domains (DBD) were found in the rice genome. According to a gene structural analysis, all members of the *OsHSF* family share Gly-66, Phe-67, Lys-69, Trp-75, Glu-76, Phe-77, Ala-78, Phe-82, Ile-93, and Arg-96. Rice HSF family genes are widely distributed in the vegetative organs, first in the roots and then in the leaf and stem; in contrast, in reproductive tissues, the embryo and lemma exhibit the highest levels of gene expression. According to chromosomal localization, tandem duplication and repetition may have aided in the development of novel genes in the rice genome. *OsHSFs* have a significant role in the regulation of gene expression, regulation in primary metabolism and tolerance to environmental stress, according to gene networking analyses.

**Conclusion:**

Six genes viz; *Os01g39020*, *Os01g53220*, *Os03g25080*, *Os01g54550*, *Os02g13800* and *Os10g28340* were annotated as promising genes. This study provides novel insights for functional studies on the *OsHSFs* in rice breeding programs. With the ultimate goal of enhancing crops, the data collected in this survey will be valuable for performing genomic research to pinpoint the specific function of the HSF gene during stress responses.

**Supplementary Information:**

The online version contains supplementary material available at 10.1186/s12870-023-04399-1.

## Background

The growth of plants is significantly impacted by a variety of detrimental environmental variables, including biotic and abiotic stresses [1] because they can hasten chlorophyll deterioration and reduce photosynthetic efficiency. The abiotic stresses like high temperatures and drought are particularly important because they can severely restrict plant growth, development, and function. Due to sessile structure of plants, which precludes them from actively avoiding stress, plants are dependent on physiological and biochemical processes to withstand external extremes [2, 3]. As a result, they must create a wide range of complex and effective mechanism to maintain normal physiology, metabolism, and development under stress conditions. The transcription factors like ABRE Binding Factor and MYC are involved in calcium signaling, abscisic acid and jasmonate signaling pathways that regulate the reactive oxygen species (ROS) and cell signaling pathways [4]. For plants to be resistant to stress, transcription factor (TF) gene expression is essential. For the reception and transmission of signals, eukaryotes usually contain a set of transcription factors called heat shock factors (HSFs). Plant stress response and the tolerance to heat are induced by the discovery of heat shock factors and the regulation of downstream genes [5, 6]. Numerous studies have documented interactions between heat and oxidative stress in the cellular pathways. The production of ROS is regarded to be a link between stressful situations like flooding, exposure to UV radiation, pathogen attack etc. [7]. Previously, it was proposed that redox responsive transcription factors like *HSFA4a* are probably responsible for detecting ROS levels in *Arabidopsis*. These "sensors" are thought to function upstream in a cascade that controls some stress-responsive proteins and other TF, including Zat and WRKY gene families [8]. In-depth investigation has shown that a variety of HSFs, including HSFA1b, HSFA4a, and HSFA8, are suspected of taking part in abiotic stress-induced ROS regulated gene networks. It is proposed, the generation of various ROS triggers HSF activation, which in turn causes the regulation of other genes. These mechanisms could act as a molecular bridge between the cellular response to heat stress and other types of stresses [9].

Heat shock transcription factors are the primary regulatory components of the plant towards heat stress response. The sequence of the *Arabidopsis thaliana* genome revealed 21 open reading frames (ORFs) that encode putative HSFs that were divided into three groups as A, B and C based on phylogenetic analysis and structural features [10]. The DNA binding domain (DBD), which interacts with "heat-shock elements" (5′-nGAAnnTTCn-3′) regulatory sequences found in the target gene via the helix-turn-helix motif and the oligomerization domain, which is responsible for HSF trimerization and has a bipartile heptad repeat pattern in the hydrophobic-associated region (HR-A/B) [11]. The HSF gene family has been characterized in several plant species, including *A. thaliana* [12], *Brachypodium distachyon* [13], *Glycine max* [14], *Solanum lycopersicum* [15], *Populus trichocarpa* [16], *Triticum aestivum* and *Zea mays* [17–19].

However, the function of HSFs in rice plant growth and development, responses to stressors and transcript expression profiling of HSFs gene has not been thoroughly investigated. The computational biology methods offer a practical and stable foundation on which additional wet-lab research could be carried out. Numerous abiotic stresses have been connected to HSF genes. In the study, we examined this important gene family in detail using the whole annotated rice genome sequence (TIGR Rice Annotation release 7).

## Material and methods

### Identification of HSF genes in *Oryza sativa* genome

The genome of the *Oryza sativa* L. japonica cultivar Nipponbare was initially mined for HSF genes using ESTs and cDNA sequences. The National Centre for Biotechnology Information (NCBI) https://www.ncbi.nlm.nih.gov/ [20], the Database of Rice Transcription Factors (DRTF) http://planttfdb.gao-lab.org/index.php?sp=Osj [21], MSU Rice Genome Annotation Project Database http://rice.uga.edu/ [22] and Plant Genome Database (PlantGDB) https://www.plantgdb.org/ [23] were used to mined the HSF genes. HSF genes in the rice genome were predicted using the BLAST online tool available at http://rice.uga.edu/analyses search blast.html on the RAP-DB website [24]. The sequences with more than 80% coverage in the BLAST analysis were found using the online tool GENSCAN (http://hollywood.mit.edu/GENSCAN.html). On both sides of the hit, the open reading frame (ORF) was expanded by around 2000 bp [25]. Additionally, the HSF domains in the query sequences were validated using the SMART (Simple Modular Architecture Research Tool) programme (http://smart.embl-heidelberg.de/).

### Phylogenetic and MEME motif analysis

Through the use of Clustal Omega (https://www.ebi.ac.uk/Tools/msa/clustalo/), the protein sequences obtained from several public repositories were aligned to remove the redundant sequences. Bootstrap (5000 replicates) and pairwise deletion were used as the default parameters to create a combined unrooted neighbor-joining (NJ) tree. Besides, the conserved motifs in HSF rice protein sequences were combed using online tool Multiple Em for Motif Elicitation (MEME Suite version 5.5.0) https://meme-suite.org/meme/tools/meme.

### Distribution of intron and exon size in *OsHSF* family genes

Using the Gene Structure Display Server (GSDS) http://gsds.gao-lab.org/, the positions of introns and exons in *OsHSF* genes were determined by gaps discovered during the alignment of full-length cDNA transcripts with genomic sequences [5]. Concisely, exons are proximal blocks of homologous sequence between full-length cDNA and genomic sequences. The introns are gaps between exons that are wholly made of genomic sequence for a single full-length cDNA that was matched to a conterminous stretch of genomic sequence [26]. To better comprehend the range and magnitude of HSF family genes, the total length of a gene is estimated by adding the lengths of each of its exons.

### Chromosomal localization

Chromosomal localization of *OsHSF* family genes was constructed using Tbtool https://github.com/CJ-Chen/TBtools/releases.

### Protein 3D structure

**Fig. 1:**
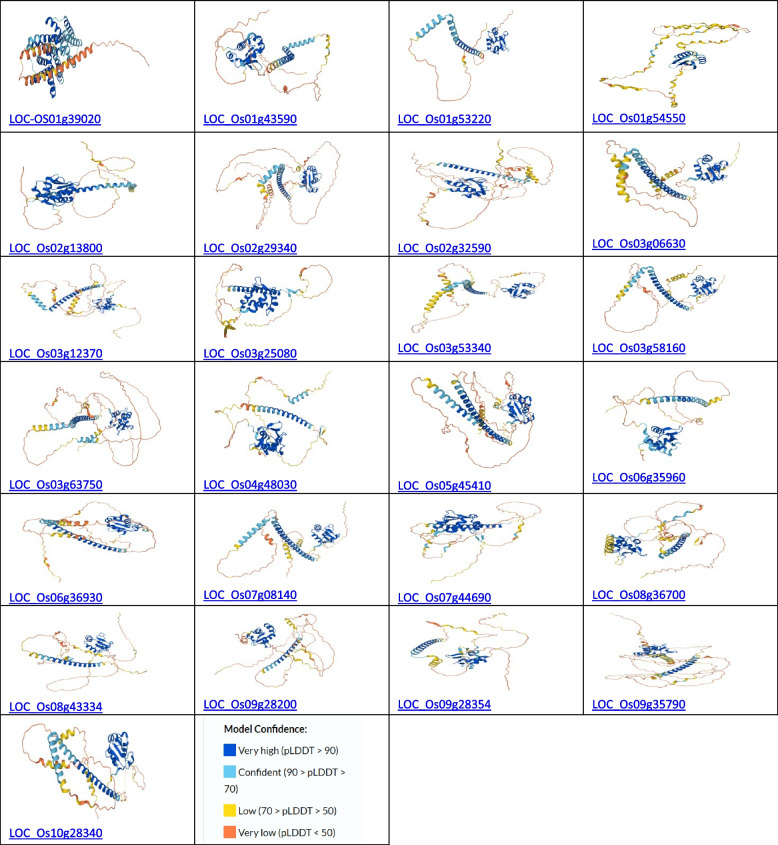
Protein structure of rice HSFs. The prediction model confidence level is presented at the bottom

Using the online programme AlphaFold, available at https://alphafold.ebi.ac.uk/ [27], the 3-D structure of the HSFs rice genes was predicted, as shown in Fig. 1.

### Gene expression analysis

The rice expression profile database (RiceXPro) [28], a public repository of gene expression, was utilized to analyze and confirm the expression of the *OsHSF* gene (s). The data from microarray experiments were used to study the entire life cycle of the rice plant, including field development (leaf day time, root day time, leaf sunset, leaf night time, root night time, reproductive organs, grain at early stage, grain ripening, spatio-temporal profile), and plant hormones (abscisic acid, auxin, brassinosteroid, cytokinin, gibberellin, and jasmonic acid in root and shoot). The most precise quantitative measurement of the transcript levels for particular genes is produced by creating a table of normalised signal intensity values for each gene in each plant tissue.

## Results

### Identification and chromosomal distribution of *OsHSF*s

**Fig. 2:**
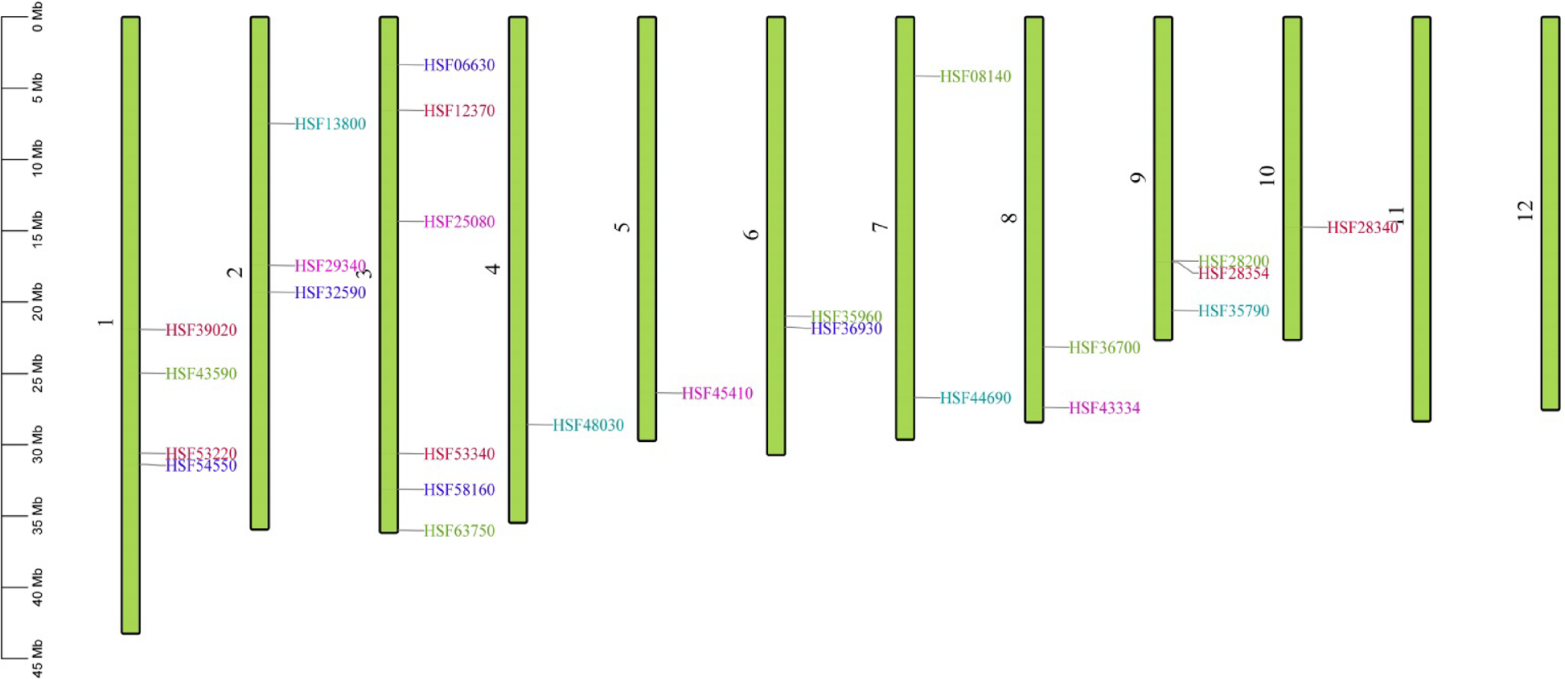
Localization of HSF family genes on rice chromosomes

With the development of genomic sequencing technology, it is now possible to recover the protein/nucleotide sequences of all *OsHSFs* family genes. After eliminating the duplicated sequences, 25 *OsHSFs* were discovered in the study, as indicated in Table 1. Using HMM and EMBL-EBI, all *OsHSF* proteins were evaluated for the presence of DBD. The SMART online tool certified the *OsHSFs*-DBDs. Table 2 lists all of the properties of the *OsHSF* genes. The 25 HSF genes were localized on rice chromosomes as shown in Fig. 2. Chromosome-1 and chromosome-3 had a maximum of 5 and 6 *OsHSF* genes respectively, whereas a single copy of *OsHSFs* gene was localized on chromosomes-4 and chromosome-5. In contrast, chromosomes-6, chromosome-7 and chromosome-8 harbor three paralogus genes, while two paralogus gene were identified on each of chromosomes-2 and chromosome-9 respectively. Except for *OsHSF13800*, *OsHSF06630*, *OsHSFSS12370*, *OsHSF25080* and *OsHSF08140*, all other *OsHSF* genes were confined on the lower arm of the chromosomes.

**Table 1:** Basic information of HSF gene family

Sr.No	Accession number		Gene Name	Protein Name	Description
	Locus ID	MSU ID			
1	Os01g0571300	LOC_Os01g39020	HSFA7	(HEAT STRESS TRANSCRIPTION FACTOR A7)	Similar to Heat shock transcription factor 31 (Fragment)
2	Os01g0625300	LOC_Os01g43590	HSFC1A	(HEAT STRESS TRANSCRIPTION FACTOR C1A)	Similar to Heat shock transcription factor 31 (Fragment)
3	Os01g0733200	LOC_Os01g53220	HSFC1B	(HEAT STRESS TRANSCRIPTION FACTOR C1b)	Similar to Heat shock transcription factor 29 (Fragment)
4	Os01g0749300	LOC_Os01g54550	HSFA4A	(HEAT STRESS TRANSCRIPTION FACTOR A4a)	Similar to Heat shock factor
5	Os02g0232000	LOC_Os02g13800	HSFC2A	(HEAT STRESS TRANSCRIPTION FACTOR C2A)	Similar to Heat shock transcription factor 29 (Fragment)
6	Os02g0496100	LOC_Os02g29340	HSFA5	(HEAT STRESS TRANSCRIPTION FACTOR A5)	Winged helix repressor DNA-binding domain containing protein
7	Os02g0527300	LOC_Os02g32590	HSFA3	(HEAT STRESS TRANSCRIPTION FACTOR A3)	Similar to Heat shock transcription factor 31 (Fragment)
8	Os03g0161900	LOC_Os03g06630	HSFA2D	(HEAT STRESS TRANSCRIPTION FACTOR A2D)	Similar to Heat shock factor 1 (Fragment)
9	Os03g0224700	LOC_Os03g12370	HSFA9	(HEAT STRESS TRANSCRIPTION FACTOR A9)	Similar to HSP protein (Fragment)
10	Os03g0366800	LOC_Os03g25080 LOC_Os03g25120	HSFB4d	(HEAT STRESS TRANSCRIPTION FACTOR B4d	Cyclin-like F-box domain containing protein
11	Os03g0745000	LOC_Os03g53340	HSFA2A	(HEAT STRESS TRANSCRIPTION FACTOR A2A)	Winged helix repressor DNA-binding domain containing protein
12	Os03g0795900	LOC_Os03g58160	HSFA2E	(HEAT STRESS TRANSCRIPTION FACTOR A2E)	Similar to Heat shock transcription factor 31 (Fragment)
13	Os03g0854500	LOC_Os03g63750	HSFA1	(HEAT STRESS TRANSCRIPTION FACTOR A1)	Similar to Heat shock transcription factor 31 (Fragment)
14	Os04g0568700	LOC_Os04g48030	HSFB2A	(HEAT STRESS TRANSCRIPTION FACTOR B2A)	Similar to Heat stress transcription factor Spl7 (Heat shock transcription factor) (Heat shock factor RHSF10)
15	Os05g0530400	LOC_Os05g45410	SPL7, HSFA4D	(HEAT STRESS TRANSCRIPTION FACTOR A4D)	Heat stress transcription factor Spl7 (Heat shock transcription factor) (Heat shock factor RHSF10)
16	Os06g0553100	LOC_Os06g35960	HSFC2B	(HEAT STRESS TRANSCRIPTION FACTOR C2B)	Heat shock factor (HSF)-type, DNA-binding domain containing protein
17	Os06g0565200	LOC_Os06g36930	HSFA6	(HEAT STRESS TRANSCRIPTION FACTOR A6)	Winged helix repressor DNA-binding domain containing protein
18	Os07g0178600	LOC_Os07g08140	HSFA2B	(HEAT STRESS TRANSCRIPTION FACTOR A2B)	Similar to Heat shock transcription factor 29 (Fragment)
19	Os07g0640900	LOC_Os07g44690	HSFB4B	(HEAT STRESS TRANSCRIPTION FACTOR B4B)	Similar to Heat shock transcription factor 33 (Fragment)
20	Os08g0471000	LOC_Os08g36700	HSFB4A	(HEAT STRESS TRANSCRIPTION FACTOR B4A	Similar to Heat shock factor
21	Os08g0546800	LOC_Os08g43334	HSFB2B	(HEAT STRESS TRANSCRIPTION FACTOR B2B)	Similar to Heat shock transcription factor 33 (Fragment)
22	Os09g0455200	LOC_Os09g28200	HSFB4C	(HEAT STRESS TRANSCRIPTION FACTOR B4C)	Winged helix repressor DNA-binding domain containing protein
23	Os09g0456800	LOC_Os09g28354	HSFB1	(HEAT STRESS TRANSCRIPTION FACTOR B1)	Similar to Heat stress transcription factor Spl7 (Heat shock transcription factor) (Heat shock factor RHSF10)
24	Os09g0526600	LOC_Os09g35790	HSFB2C	(HEAT STRESS TRANSCRIPTION FACTOR B2C)	Similar to Heat shock factor protein 3 (HSF 3) (Heat shock transcription factor 3) (HSTF 3)
25	Os10g0419300	LOC_Os10g28340	HSFA2C	(HEAT STRESS TRANSCRIPTION FACTOR A2C)	Similar to Heat shock transcription factor 31 (Fragment)

**Table 2:** Features of HSF gene family for chromosomal localization

Sr. No	MSU ID	Chr. No	Start	End	Strand	CDS BP	Protein length	Molecular weight	PI
**1**	LOC_Os01g39020	1	21,938,865	21,941,839	forward	1209	403	43,911.9883	7.47249985
**2**	LOC_Os01g43590	1	31,370,225	31,372,759	forward	1323	441	49,387.3789	5.0935998
	LOC_Os01g43590	1	24,967,398	24,969,047	forward	1020	340	36,862.7305	6.69099998
**3**	LOC_Os01g53220	1	30,582,485	30,583,743	forward	753	251	27,219.0293	8.94960022
**4**	LOC_Os01g54550	1	31,370,225	31,372,759	forward	1323	441	49,387.3789	5.0935998
**5**	LOC_Os02g32590	2	19,313,057	19,309,594	forward	1497	499	55,098.5391	4.50330019
**6**	LOC_Os02g29340	2	17,428,027	17,431,373	forward	1428	476	52,884.6797	5.17430019
**7**	LOC_Os02g13800	2	7,463,932	7,465,506	forward	897	299	31,919.5	6.51760006
**8**	LOC_Os03g53340	3	30,607,164	30,604,067	forward	1131	377	40,847.5312	4.71829987
	LOC_Os03g53340	3	30,607,164	30,603,964	forward	1131	377	40,847.5312	4.71829987
	LOC_Os03g53340	3	30,607,159	30,604,067	forward	1131	377	40,847.5312	4.71829987
	LOC_Os03g53340	3	30,607,152	30,604,067	forward	1131	377	40,847.5312	4.71829987
**9**	LOC_Os03g12370	3	6,537,569	6,541,400	forward	1233	411	45,465.6406	4.81960011
	LOC_Os03g12370	3	6,537,569	6,540,308	forward	1221	407	45,052.1289	4.88030005
**10**	LOC_Os03g25080	3	14,345,575	14,342,679	forward	918	306	33,950.9414	6.34940004
**11**	LOC_Os03g63750	3	35,989,011	35,992,635	forward	1521	507	55,277.2188	4.72679996
**12**	LOC_Os05g45410	5	26,344,414	26,346,889	forward	1380	460	51,163.8711	4.94820023
**13**	LOC_Os06g35960	6	20,998,867	20,996,264	forward	837	279	28,971.2695	8.23719978
**14**	LOC_Os06g36930	6	21,761,304	21,762,421	forward	996	332	36,103.3281	4.63579988
**15**	LOC_Os07g44690	7	26,673,639	26,676,932	forward	933	311	34,450.7812	7.0637002
**16**	LOC_Os08g36700	8	23,159,531	23,158,296	forward	1143	381	41,386.7305	9.63790035
**17**	LOC_Os08g43334	8	27,390,339	27,380,765	forward	1851	617	66,415.6094	8.41919994
	LOC_Os08g43334	8	27,384,520	27,382,865	forward	1173	391	41,374.0508	5.0078001
**18**	LOC_Os09g28200	9	17,111,077	17,109,289	forward	1185	395	42,036.1094	8.64169979
**19**	LOC_Os09g35790	9	20,595,143	20,591,230	forward	1365	455	47,014.1797	4.91120005
	LOC_Os09g35790	9	20,595,143	20,593,125	forward	1245	415	42,580.1914	4.95979977
**20**	LOC_Os03g06630	3	3,342,254	3,344,548	forward	1140	380	43,706.6094	8.90200043
	LOC_Os03g06630	3	3,342,254	3,344,548	forward	1080	360	41,134.6406	6.9769001
**21**	LOC_Os03g58160	3	33,105,828	33,109,091	forward	1074	358	40,258.9102	5.82919979
	LOC_Os03g58160	3	33,105,840	33,109,091	forward	591	197	22,186.0898	9.32019997
**22**	LOC_Os04g48030	4	28,574,411	28,576,248	forward	918	306	32,809	4.94759989
**23**	LOC_Os07g08140	7	4,139,160	4,142,449	forward	1119	373	41,524.1289	4.38819981
**24**	LOC_Os09g28354	9	17,221,426	17,228,961	forward	909	303	32,798.9805	9.71920013
	LOC_Os10g28340	10	14,750,175	14,746,825	forward	1077	359	40,784.6406	4.65320015
**25**	LOC_Os10g28340	10	14,750,175	14,746,101	forward	1077	359	40,784.6406	4.65320015

### Phylogenetic classification of *OsHSF*s family genes

**Fig. 3:**
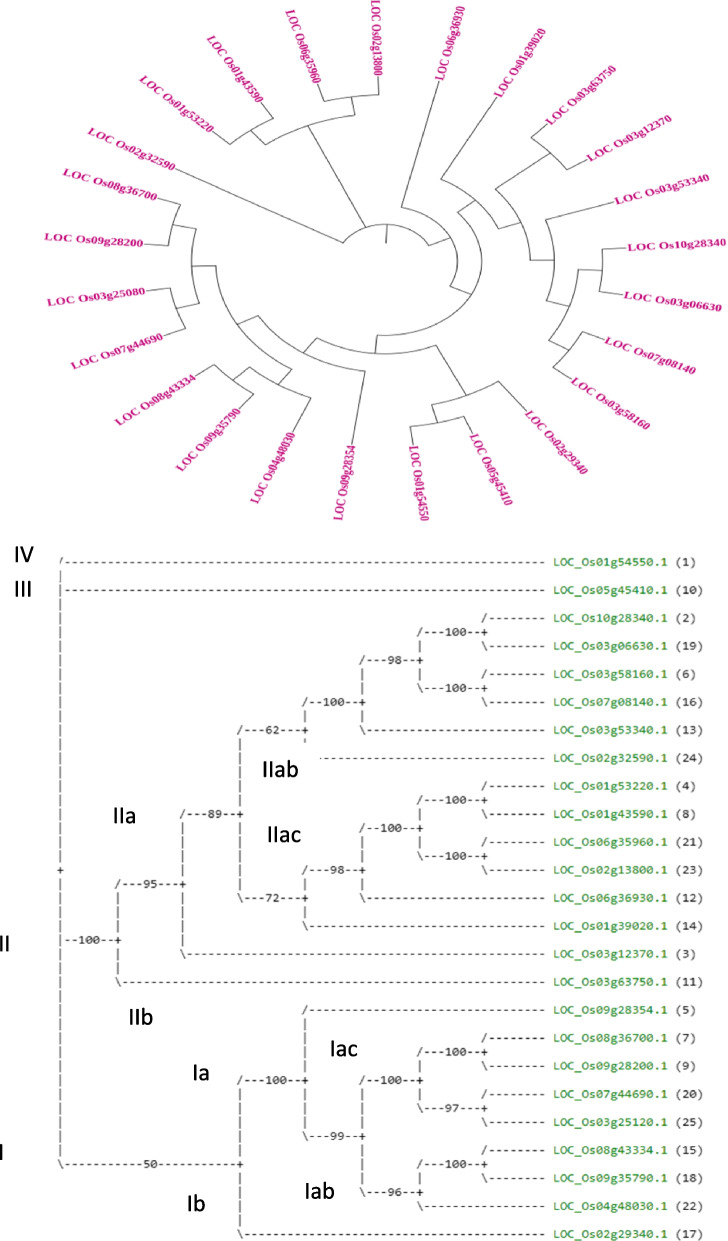
Protein based phylogenetic tree of HSF gene family

These findings led to the construction of a phylogenetic tree for 25 *OsHSF* genes using bootstrap analysis (5000 replicates) based on multiple alignments of protein sequences (Fig. 3). The phylogram is alienated into a total of four clades namely clade-I to clade-IV. Clade-I is further distinguished into sub-groups: I-a, I-b and with total of nine *OsHSF* genes. With 14 *OsHSF* genes, the clade-II is further split into clade-IIa and clade-IIb. Clade IIa also has two sub-clades called clade IIab and clade IIac. Two distinct groups with a single gene each are clade-III and clade-IV. Finally, the genes are characterized into *OsHSF* proteins, which are applied for abiotic factors like heat shock and drought resistance.

**Fig. 4:**
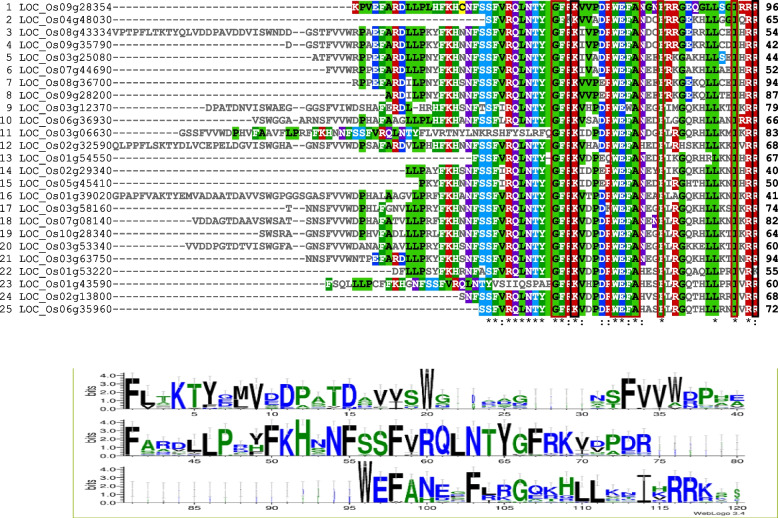
Multiple allignment of protein sequence and DNA binding domain (DBD) of HSF gene family

Using the amino acid sequences of the *OsHSF* domains, multiple alignment analysis was carried out to dissect the evolutionary relationships amongst *Oryza sativa* HSF family members. The alignment predicts that Ser-55, Ser-56, Phe-57, Val-58, Arg-59, Gln-60, Leu-61, Asn-62, Thr-63, Tyr-64, Arg-68, Val-70, Val-71, Pro-72, Asp-73, Arg-74, Asn-79, Gly-85 and Leu-89 are highly conserved whereas Gly-66, Phe-67, Lys-69, Trp-75, Glu-76, Phe-77, Ala-78, Phe-82, Ile-93 and Arg-96 are completely conserved in all *OsHSFs* family members in rice (Fig. 4). More than 10% of *OsHSFs* members have completely conserve amino acid residues whereas more than 19% *OsHSFs* amino acids are highly conserved in the *OsHSFs* domain. Multiple alignments of protein sequence and DBD of *OsHSFs* gene family are given in Fig. 4.

### Characteristics of each group in the rice HSFs family genes

The responses of these genes to abiotic factors have been documented in *Arabidopsis*, *Brachypodium* and *Oryza* species. These genes must be classified in accordance with various stress regimes in order to be included in unique groups based on their protein similarity, which may aid in related function within their evolutionary placement. The Table 4 provides a summary on the roles of each gene in the *OsHSF* family. The two sub-groups of Clade-I, Ia and Ib, harbour nine total genes. All genes in this group are involved in root development, vegetative growth and reproductive stages (embryo development). These genes have resistance against water stress in early seed germination and at the time of flowering during high temperature.

The clade-II comprised of 14 genes and involved in anther, ovary, embryo and endosperm and root development. In Clade-III and clade-IV has single gene. The gene in the clade-III is involved in vegetative i.e. leaf blade and root development along with reproductive (pistil and palea development). The gene in clade-IV involved in leaf blade, leaf sheath, stem, vegetative and root development and reproductive (pistil, palea, lemma anthers and inflorescence).

### Distribution of motifs

**Fig. 5:**
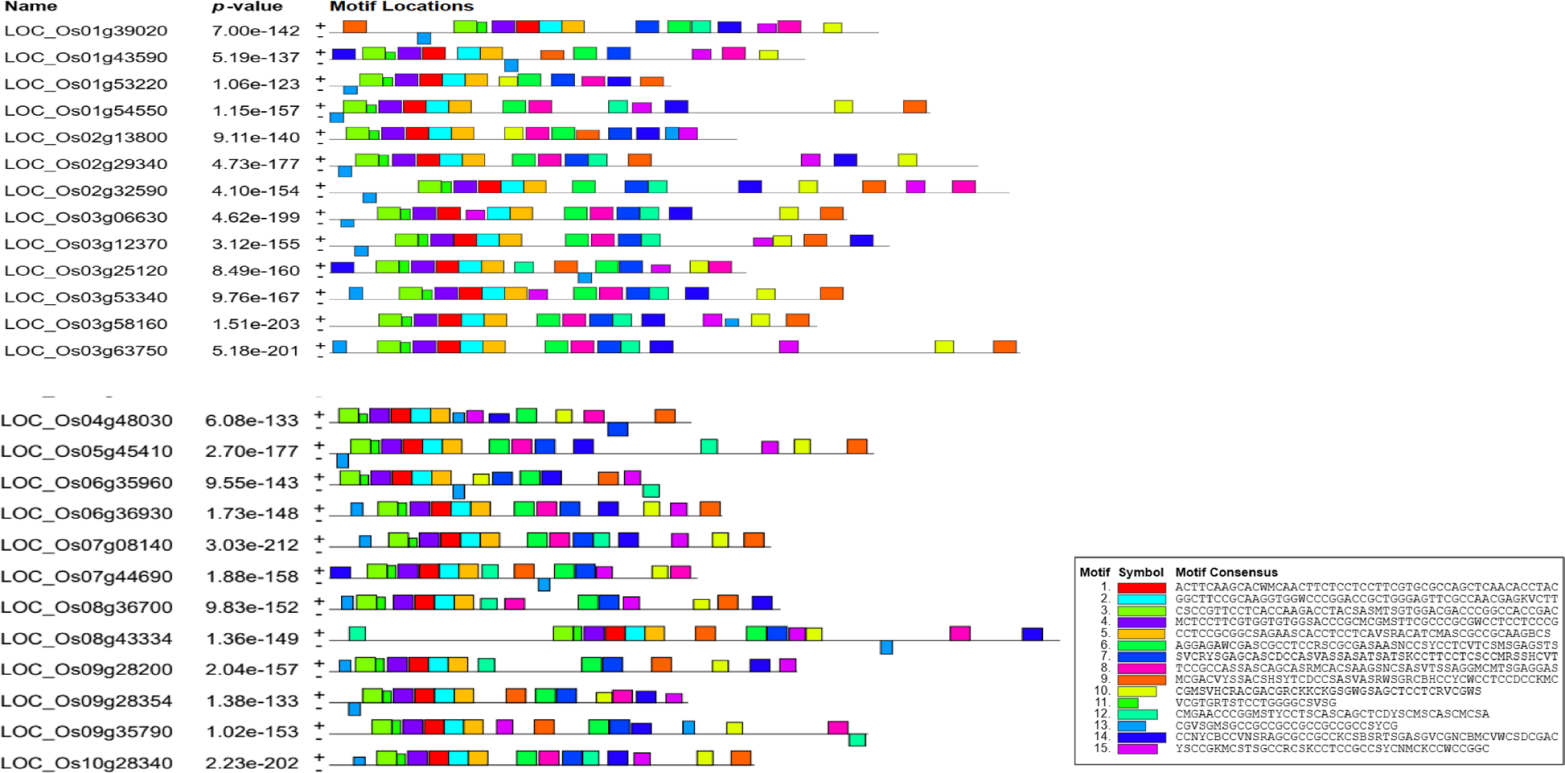
Motif location of HSF gene family

*OsHSFs* TF contain functionally important motifs linked to mitochondria and chloroplasts. Such functional sequencing motifs are typically conserved among members of a subgroup in vast families of transcription factors in plants, and the proteins of these motifs in their subgroups are likely to have similar activities. Multiple alignment analysis with Clustal Omega was used to investigate the conserved motifs in the nucleotides of each clade in the rice *OsHSFs* gene family. The MEME Suite version 5.5.0 was used to examine rice *OsHSFs* protein sequences for the presence of conserved motifs. Overall, 15 conserved motifs were predicted which correspond to the *OsHSFs* domain as shown in Fig. 5 and these conserved motifs found in the *OsHSFs* family are listed in Table 3.

**Table 3:** Conserved motif sequence of Oryza sativa L. HSFs

Motif	Consensus Sequence
1	ACTTCAAGCACWMCAACTTCTCCTCCTTCGTGCGCCAGCTCAACACCTAC
2	GGCTTCCGCAAGGTGGWCCCGGACCGCTGGGAGTTCGCCAACGAGKVCTT
3	CSCCGTTCCTCACCAAGACCTACSASMTSGTGGACGACCCGGCCACCGAC
4	MCTCCTTCGTGGTGTGGSACCCGCMCGMSTTCGCCCGCGWCCTCCTCCCG
5	CCTCCGCGGCSAGAASCACCTCCTCAVSRACATCMASCGCCGCAAGBCS
6	AGGAGAWCGASCGCCTCCRSCGCGASAASNCCSYCCTCVTCSMSGAGSTS
7	SVCRYSGAGCASCDCCASVASSASATSATSKCCTTCCTCSCCMRSSHCVT
8	TCCGCCASSASCAGCASRMCACSAAGSNCSASVTSSAGGMCMTSGAGGAS
9	MCGACVYSSACSHSYTCDCCSASVASRWSGRCBHCCYCWCCTCCDCCKMC
10	CGMSVHCRACGACGRCKKCKGSGWGSAGCTCCTCRVCGWS
11	VCGTGRTSTCCTGGGGCSVSG
12	CMGAACCCGGMSTYCCTSCASCAGCTCDYSCMSCASCMCSA
13	CGVSGMSGCCGCCGCCGCCGCCGCCSYCG
14	CCNYCBCCVNSRAGCGCCGCCKCSBSRTSGASGVCGNCBMCVWCSDCGAC
15	YSCCGKMCSTSGCCRCSKCCTCCGCCSYCNMCKCCWCCGGC

### Gene structure analysis

**Fig. 6:**
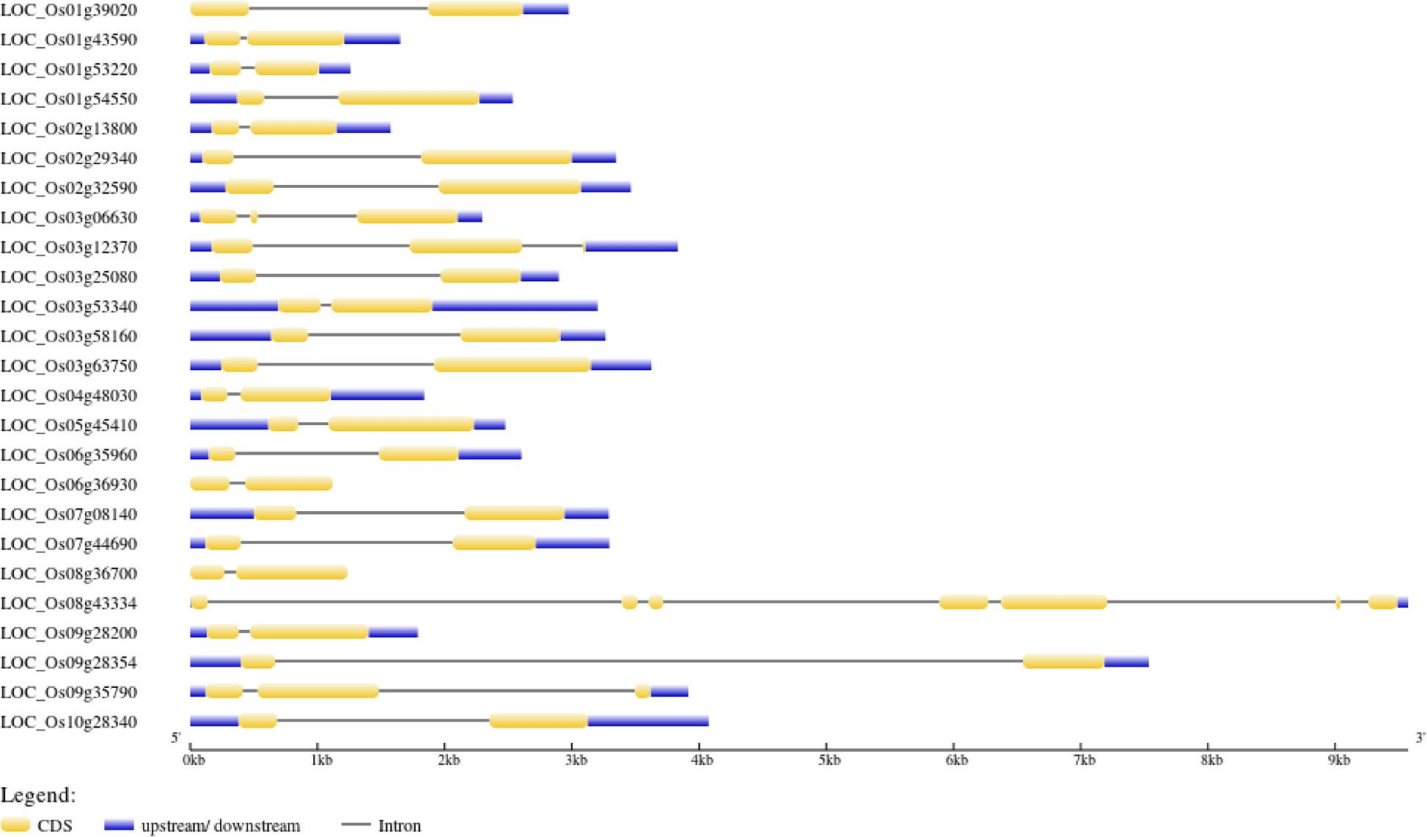
Intron/exon size distribution of HSF family gene

The GSDS tool was used to examine the intron–exon organization of the selected *OsHSFs* in order to determine the structural link between the genes. The quantity and structure of introns and exons have a significant impact on how gene families have evolved. The number of exons and introns was found to remain constant, and 84% (21/25) of *OsHSFs* contain just one intron for *Os08g43334*, *Os03g12370*, *Os09g35790* and *Os03g06630* (Fig. 6). The remaining *OsHSF* gene family has two introns. Exon counts for the *Os08g43334*, *Os03g12370*, *Os09g35790*, and *Os03g06630* revealed seven, two, three, and three exons, respectively. All *OsHSF*s contained 5′ and 3′ un-translated region (UTR). In terms of intron number, intron phase, exon length, and overall gene length, similar intron–exon patterns were observed in the *OsHSF* genes belonging to the same class and subclasses.

### Expression profiles of *OsHSFs* at different developmental stages

**Fig. 7:**
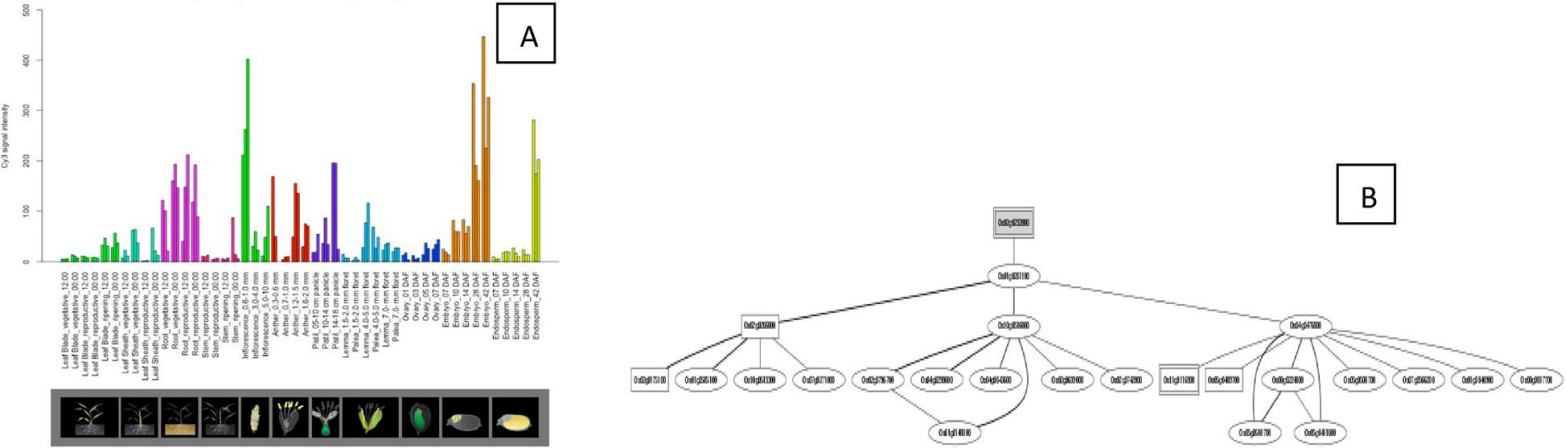
Role of *Os02g13800* gene in field development (**A**) and network image (**B**)

Investigations into the *OsHSF* gene expression patterns were conducted on various time scales and at various growth stages. Transcriptome profiles provide insight into the potential role of genes in a variety of biological processes, despite the fact that protein expression is not always associated with gene expression. The rice genome database RiceXpro was used to download the transcriptome data that was used in the current study. The *Os01g39020*, *Os01g53220*, *Os03g25080*, *Os01g54550*, *Os10g28340*, and *Os02g13800* have been demonstrated to be among the tissues with the highest up-regulation of *OsHSFs* during different growth phases (Fig. 7). The *Os01g39020*, *Os01g53220*, *Os01g54550*, *Os02g29340*, *Os03g58160*, *Os03g63750*, *Os06g35960*, *Os07g08140*, *Os07g44690*, *Os09g28200*, *Os09g35790*, and *Os10g28340* are also up-regulated throughout the development of reproductive organs and grain ripening stages. A total of 4 *OsHSF*s exhibited less expression at all as shown in Table 4.

**Table 4:** Role of HSF gene family in field development

Sr. No	Locus ID	Leaf sunrise	Leaf day time	Root day time	Leaf Diurnal	Root Diurnal	Leaf sunset	leaf night time	Root night time	Reproductive organs	Grain at early stage	Grain Ripening	Spatio-temporal profile
1	Os01g0571300	4:10–4:50 am	13–55 DAT	20–55 DAT	Vegetative to Reproductive ripening	15, 17, 44 DAT	18:20- 20:00 pm	14–70 DAT	49–70 DAT	Inflorescence, Anther and pistil	Embryo 8–10 DAF	Embryo 10–42 DAF, Endosperm 42 DAF	embryo and endosperm DAF development
2	Os01g0625300	5:20–6:00 am	104–125 DAT	69–104 DAT	Reproductive Ripening to Ripening	15, 16, 44 DAT	17:00–17:20, 20:00 pm	112–119 DAT	56–63, 98–105 DAT	Pistil 7 mm floret, lemma 7 mm floret	ovary 4 DAF-Embryo 7 DAF	Embryo 21- 42 DAF	reproductive and ripening of Leaf blade and leaf sheath
3	Os01g0733200	3:50–4:50 am	13,41 and 125 DAT	55–69 DAT	Reproductive to Reproductive Ripening	15, 44 DAT	17:00–18:00 pm	105–119 DAT	63, 84–91 DAT	Anther 1.2-6 mm, pistil, Palea 7 mm floret, lemma 7 mm floret	Embryo 7–10 DAF	Embryo 10–42 DAF,	anther and embryo development
4	Os01g0749300	5:40–6:00 am	48–62 DAT	48–55 DAT	Reproductive to Reproductive Ripening	15, 16 DAT	17:00–17:20 pm	56–77 DAT	49–70 DAT	Inflorescence, Anther, pistil and lemma	Ovary 1 DAF	Embryo 7–14 & 28–42 DAF,	Leaf blade, leaf sheath, Root vegetative and stem reproductive
5	Os02g0232000	5:00–5:30 am	11–125 DAT	34, 55 DAT, 69 DAT	Reproductive and Ripening	16, 17 DAT	17:00–17:40 pm	14, 112–126 DAT	56–63 DAT	Inflorescence, Anther, pistil	Ovary 7–9 DAF, Embryo 9–10 DAF	Embryo 28–42 DAF, Endosperm 42 DAF	root vegetative and reproductive, inflorescence and embryo
6	Os02g0496100	4:10–4:30 am	48–62 DAT	27–48 DAT	Reproductive to Reproductive Ripening	15, 16, 43 DAT	18:00–19:50 pm	49–56, 77 DAT	49 DAT	Inflorescence 2–2.5 mm, Anther, pistil, Palea 7 mm floret, lemma 7 mm floret	Ovary 02–06 DAF	Embryo 7–10 and 28–42 DAF,	leaf blade reproductive, root vegetative
7	Os02g0527300	3:50–4:10 am	14–48 DAT	20–41 DAT	Reproductive to Reproductive Ripening	15, 16 DAT	17:00–17:20 pm	63–70, 98–105 DAT	84 DAT	Anther, pistil, Lemma	Ovary 05–07 DAF	Embryo 28–42 DAF, Endosperm 07 DAF	Pistil, lemma
8	Os03g0161900	5:30–6:00 am	55–62 DAT	20 DAT	Vegetative reproductive to ripening	15, 16 DAT	17:00–17:20 pm	49, 70–77 DAT	49, 91 DAT	pistil, Lemma, palea	ovary 01 DAF	Embryo 07, 28–42 DAF, Endosperm 7 DAF	Leaf blade vegetative and reproductive, ovary 1 DAF
9	Os03g0224700	6:00 AM	55–62, 104 DAT	20 and 48 DAT	Reproductive to Reproductive Ripening	16, 44 DAT	17:00–17:20 pm	28, 77 DAT	21, 49 DAT	Anther	ovary 01 DAF	Embryo 07 DAF, Endosperm 07 DAF	Anther and ovary development
10	Os03g0366800	4:00–6:00 am	48–62, 104–111 DAT	20–55 DAT	Vegetative reproductive to ripening	15–44 DAT	18:00–19:20 pm	84–126 DAT	49–70 DAT	Inflorescence and pistil	Embryo 5–10 DAF	Embryo 07–42 DAF	stem reproductive, inflorescence, pistil, embryo development
11	Os03g0745000	4:40–5:10 am	118–125 DAT	20 and 55 DAT	Reproductive	16, 44 DAT	17:00–17:20 pm, 18:00–18:20 pm	14, 56, 84 DAT	49–70 DAT	Anther and pistil	ovary 01 DAF	Embryo 07 DAF,	ovary 01 DAF
12	Os03g0795900	4:30–5:00 am	48–62 DAT	20–55 DAT	Reproductive to Reproductive Ripening	15, 16 DAT	17:40–19:00 pm	49–84, 98–105 DAT	14, 49–70, 84–91 DAT	Inflorescence and pistil	ovary 01–02 DAF	Embryo 7–14 & 28–42 DAF,	leaf blade reproductive, pistil, ovary
13	Os03g0854500	4:00–6:00 am	76 DAT, 104–118 DAT	41–55 DAT	Reproductive to Reproductive Ripening	15, 16 DAT	18:20–19:50 pm	98–105 DAT	49–56 DAT	Inflorescence and pistil	Embryo 05–06, 8–10 DAF,	Embryo 07–42 DAF	Leaf blade vegetative and reproductive, pistil, root vegetative and inflorescence
14	Os04g0568700	5:40–6:00 am	55–62 DAT	20 DAT	Reproductive to Reproductive Ripening	16 DAT	17:00–17:30 pm	63–77 DAT	49–70 DAT	Pistil	ovary 01 DAF	Embryo 07–10 DAF, endosperm 07 DAF	ovary 01 DAF
15	Os05g0530400	5:30–6:00 am	69, 97, 111 DAT	41–55 DAT	Reproductive	15, 43 DAT	17:00–17:20 pm, 18:10–18:30 pm	77–98 DAT	49–63, 84 DAT	Pistil, Palea	ovary 01–02 DAF	Embryo 28–42 DAF, Endosperm 10–14 DAF	Leaf blade reproductive, root reproductive, Ovary 01 DAF
16	Os06g0553100	5:20–5:30 am	13, 14, 83 DAT	27–49 DAT	Vegetative	6, 17 DAT	17:00–17:40 pm, 19:30–19:50 pm	14–28 DAT	14–35, 56 DAT	Inflorescence, Anther and pistil	Embryo 8–10 DAF	Embryo 07–42 DAF	Embryo 07–42 DAF
17	Os06g0565200	4:50–5:00 am	48–62 DAT	20, 48–62 DAT	Reproductive to Reproductive Ripening	16 DAT	17:00–17:30 pm	21–35 DAT	49–63, 84–91 DAT	Anther and pistil	ovary 01 DAF	Embryo 10 and 28–42 DAF	ovary 01 DAF
18	Os07g0178600	5:20–6:00 am	20–41 DAT	20–34 and 48 DAT	Vegetative	15–16 DAT	17:00–17:20 pm	21–35 DAT	21–35 DAT	inflorescence, pistil and lemma	Embryo 6 and 8–10 DAF	Embryo 07–42 DAF	inflorescence, pistil, embryo and lemma
19	Os07g0640900	5:10–6:00 am	20–41 DAT	20–34 and 48 DAT	Vegetative	15–16 DAT	17:00–17:20 pm	21–35 DAT	21–35 DAT	inflorescence, pistil and lemma	Embryo 6 and 8–10 DAF	Embryo 07–42 DAF	inflorescence, pistil, embryo and lemma
20	Os08g0471000	5:20–5:50 am	97 and 111–125 DAT	41–55 DAT	Reproductive	15–16 DAT, 43 DAT	18:20–18:40 pm	70–126 DAT	49–63 DAT	Lamma, palea	ovary 01 and -7–09 DAF	Endosperm, 07–21 DAF	Root vegetative, stem and root reproductive, stem ripening
21	Os08g0546800	5:50–6:00 am	62, 104 and 125 DAT	20–27 DAT	Reproductive to Reproductive Ripening	16 DAT	17:00–17:20 pm	14, 77–84 DAT	63, 84–91 DAT	Inflorescence and anther	ovary 01 DAF	Embryo 07 and 42 DAF, Endosperm 07	ovary 01 DAF
22	Os09g0455200	4:00–5:10 am	48–55 DAT	20–34 and 48 DAT	Reproductive to Reproductive Ripening	16 DAT	17:00–18:20 pm	56–70 DAT	21–35 and 49 DAT	inflorescence, pistil and lemma	ovary 1–3 DAF and endosperm 5–10	Embryo 07–42 DAF	stem reproductive, inflorescence and pistil
23	Os09g0456800	5:20–5:50 am	97 and 111–125 DAT	41–55 DAT	Reproductive	15–16, 43 DAT	18:20–18:40 pm	70–126 DAT	49–63 DAT	Lemma, palea	ovary 01 and -7–09 DAF	Endosperm, 07–21 DAF	Root vegetative, stem and root reproductive, stem ripening
24	Os09g0526600	5:40–6:00 am	62, 92–97 DAT	20–55 DAT	Reproductive to Reproductive Ripening	16 and 44 DAT	17:10–17:30 pm	14, 28 and 91 DAT	49–63 DAT	inflorescence and pistil	ovary 01 DAF	Embryo 07–42 DAF, endosperm 7, 14 and 42 DAF	Leaf blade ripening, root vegetative, ovary 01 DAF, embryo 07 DAF
25	Os10g0419300	4:40–5:40 am	34–55 DAT	20–41 DAT	Vegetative reproductive, Reproductive and Reproductive Ripening	43 DAT	17:40–18:20 pm	42–70 DAT	28–56 DAT	inflorescence, Palea and lemma	Embryo 5–8 DAF	Embryo 07–42 DAF, Endosperm 07–14 DAF	Stem reproductive and ripening, lemma, palea, ovary 01–07 and embryo 07–42

**Fig. 8:**
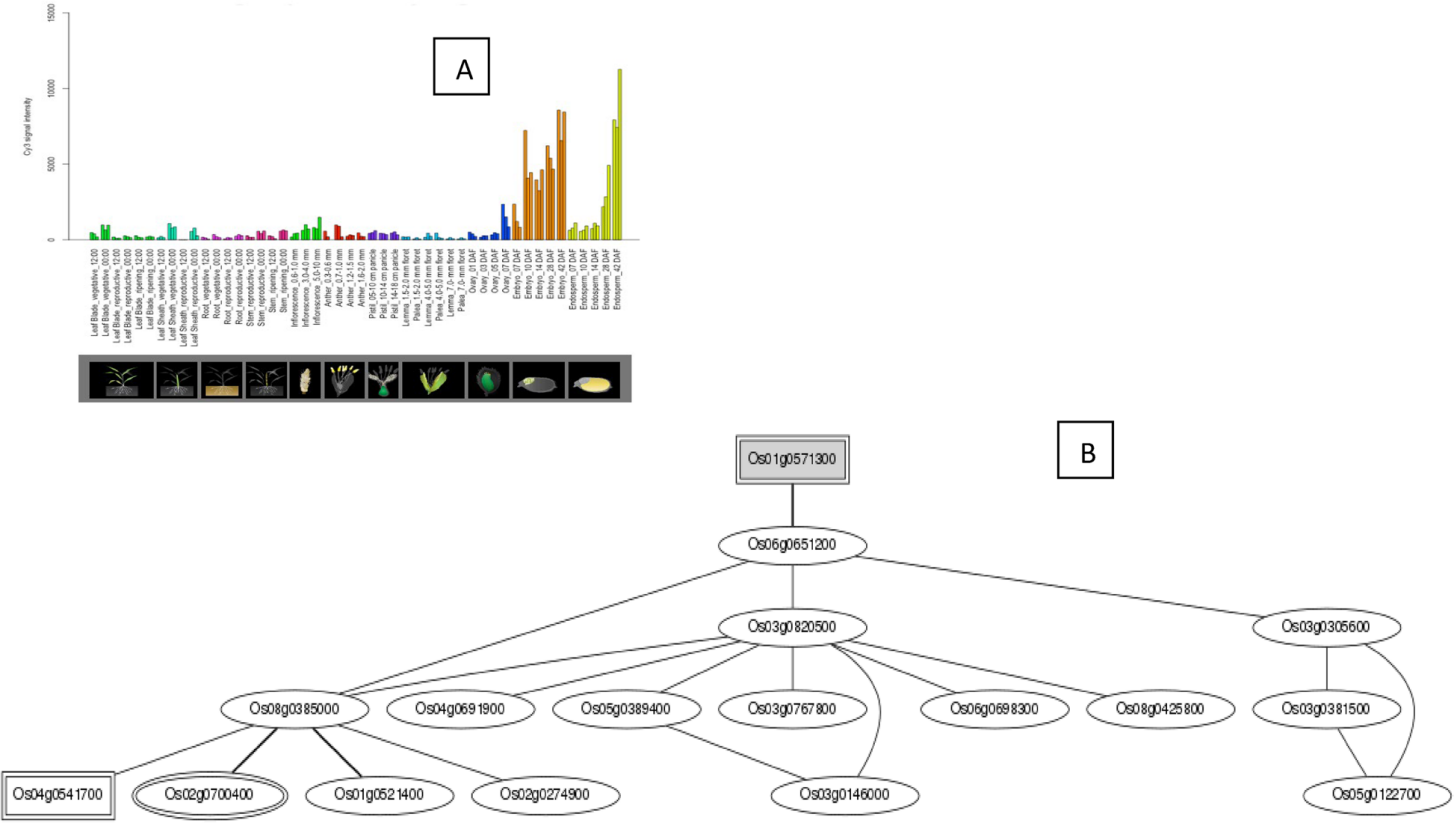
Role of *Os01g39020* gene in field development (**A**) and network image (**B**)

**Fig. 9:**
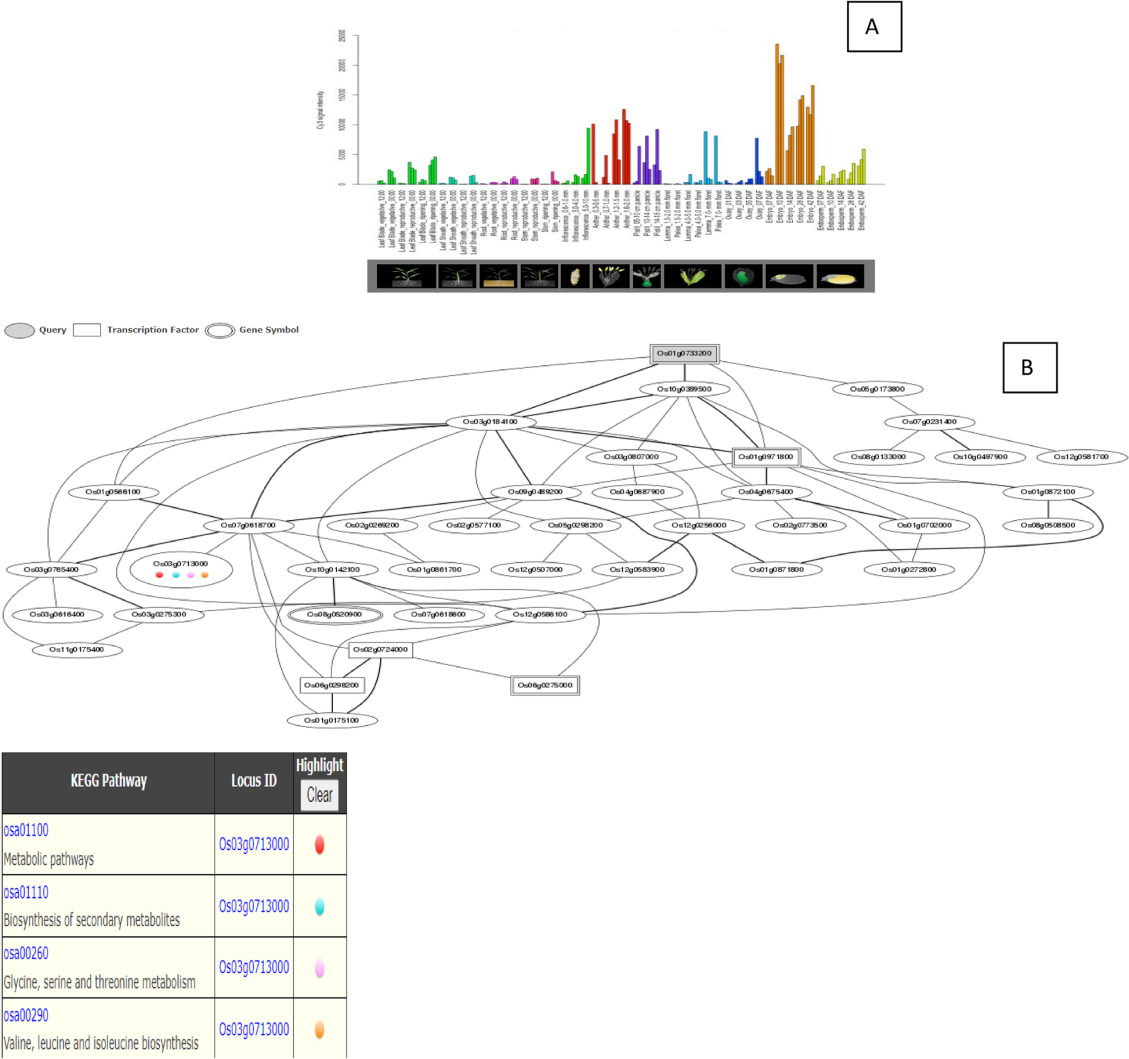
Role of *Os01g53220* gene in field development (**A**) and gene networking analysis on the basis locus ID (**B**)

**Fig. 10:**
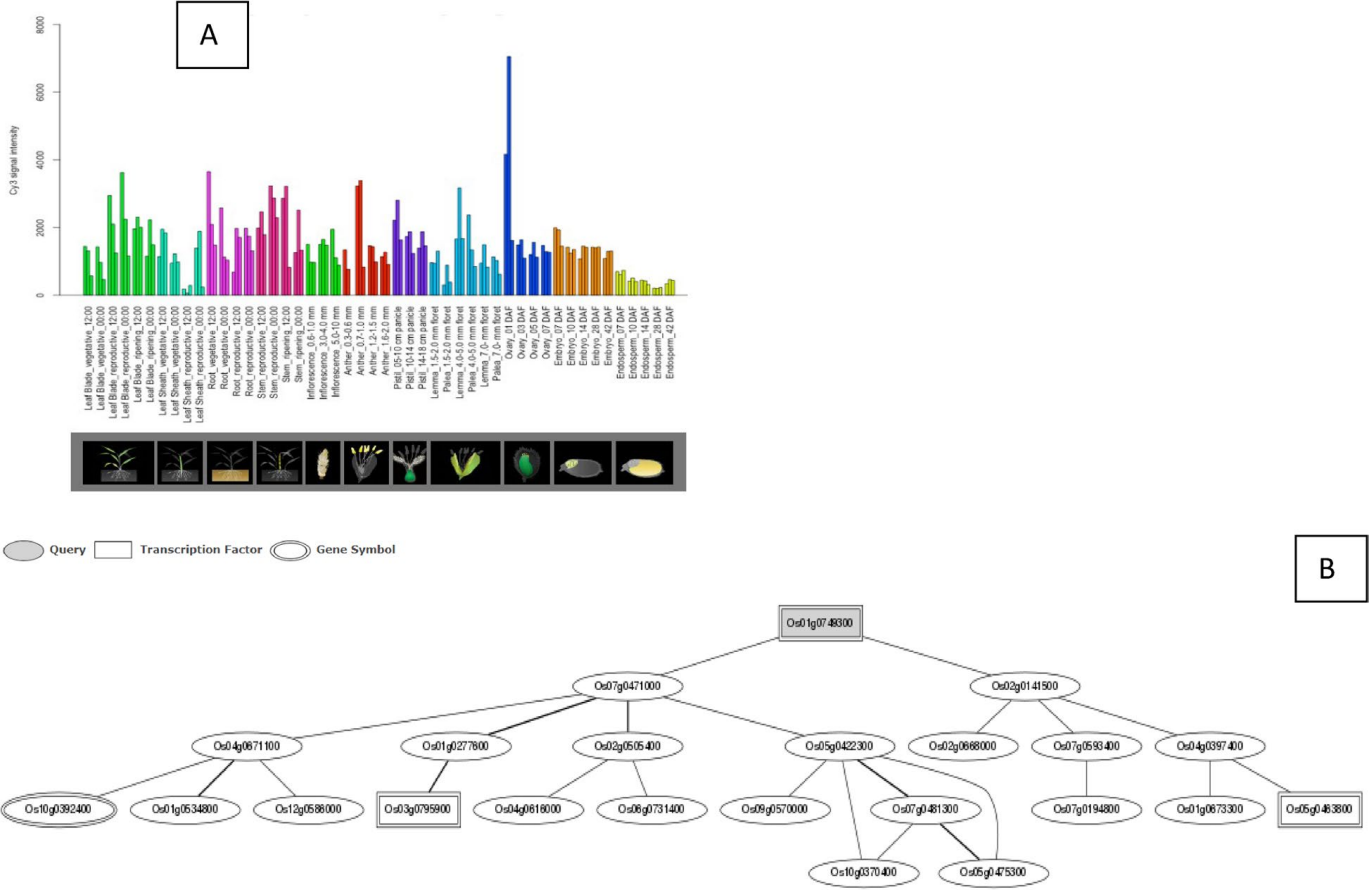
Role of *Os01g54550* gene in field development (**A**) and gene networking analysis on the basis locus ID (**B**)

**Fig. 11:**
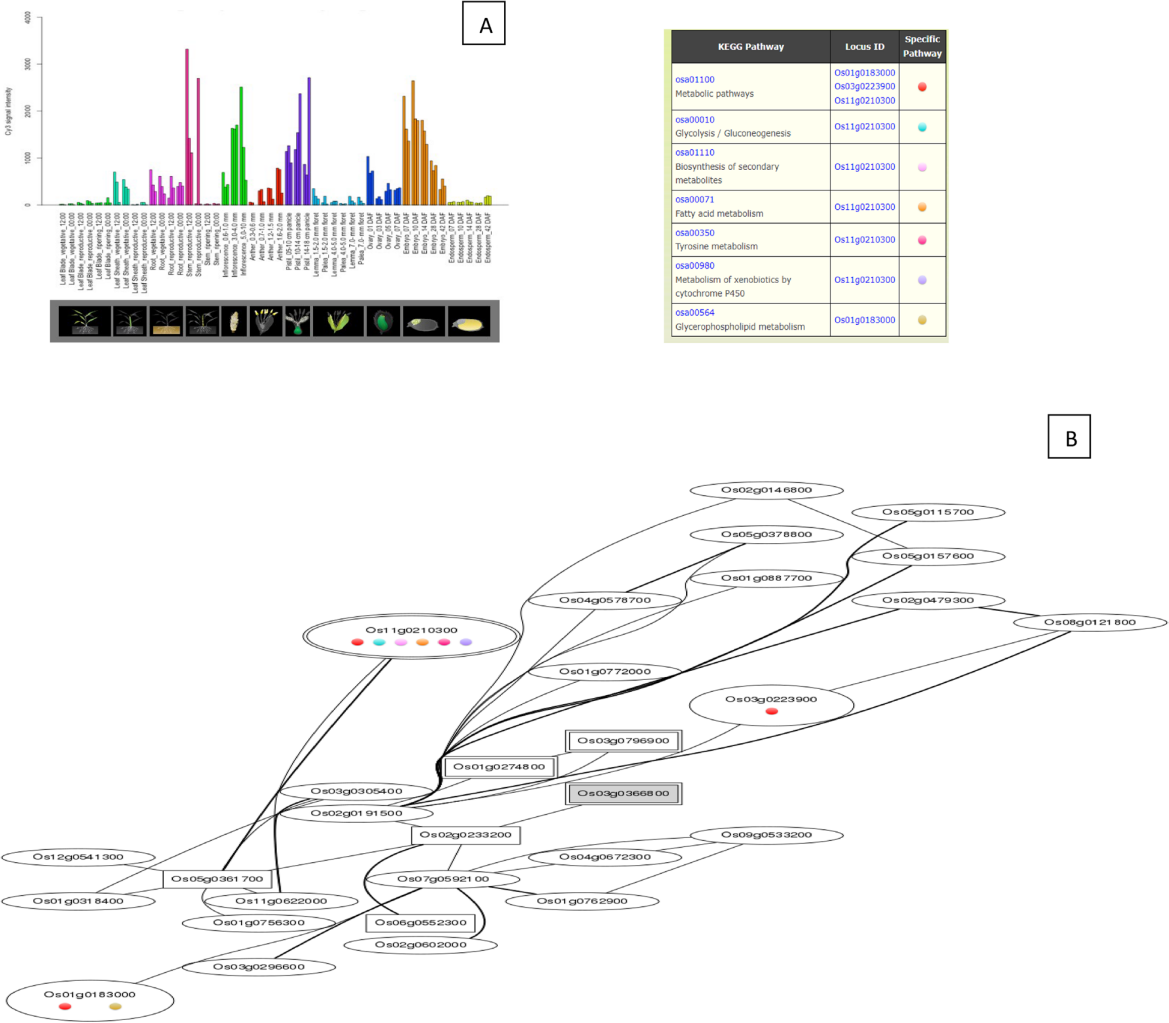
Role of *Os03g25080* gene in field development (**A**) and network image (**B**)

**Fig. 12:**
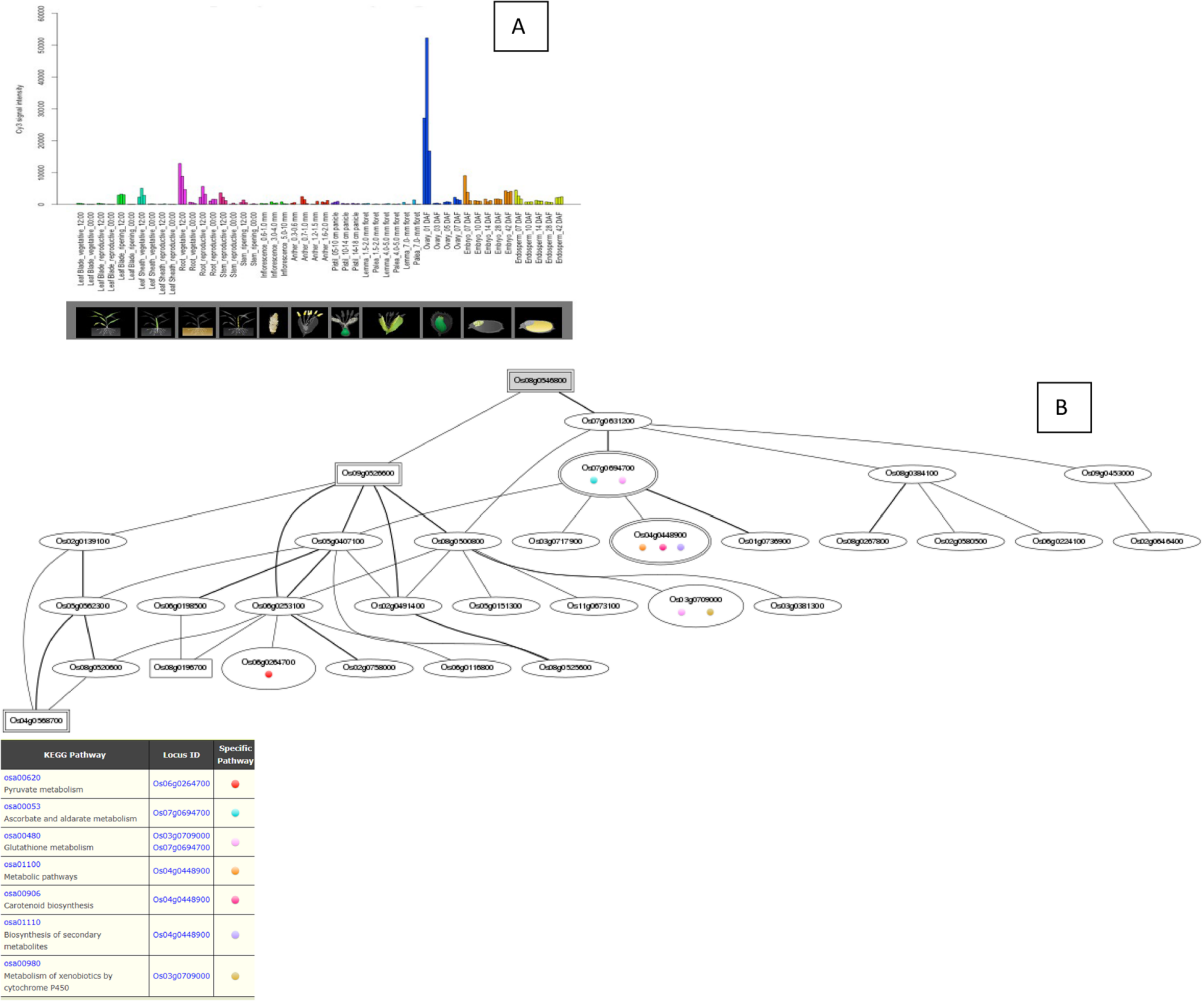
Role of *Os08g43334* gene in field development (**A**) and gene networking analysis on the basis of locus ID (**B**)

The *Os01g39020*, *Os01g43590*, *Os01g53220*, *Os02g29340*, *Os03g12370*, *Os03g53340*, and *Os09g35790* genes are up-regulated during the leaf and root diurnal stage. This suggests that these genes could potentially regulate these tissues and influence vegetative growth. In leaf tissues, *OsHSFs* were expressed most highly. In the same way, grain formation at early stage across different tissues, *Os01g39020* (Fig. 8), *Os01g53220* (Fig. 9), *Os01g54550* (Fig. 10), *Os03g25080* (Fig. 11) *Os02g29340*, *Os03g58160*, *Os03g63750*, *Os06g35960*, *Os07g08140*, *Os07g44690*, *Os08g43334* (Fig. 12)*, Os09g28200, Os09g35790* and *Os10g28340 *are up-regulated see Table 4.

### Expression profiles of *OsHSFs* at different plant hormone stages

A wide variety of plant hormones have an impact on rice growth, development, and yield. RiceXpro was used to analyze the data in order to survey rice *OsHSFs* expression in response to several plant hormones (Table 5). The genes *Os01g39020*, *Os01g53220*, *Os02g13800*, *Os03g12370*, *Os03g25080*, *Os04g48030*, *Os06g35960*, *Os07g08140*, *Os08g43334* and *Os09g28354* had the highest expression in the root and shoot in response to abscisic acid. *Os01g54550* and *Os03g25080* were moderately overexpressed in the root and shoot at various times when gibberellins were present. Only two *OsHSFs* (*Os01g39020* and *Os01g54550*) displayed considerably increased expression in the rice plant's root and shoot when auxin hormone was present. A single gene, *Os01g43590*, showed moderate expression in the root and shoot when brassinosteroid hormone was present. The genes *Os01g39020*, *Os01g53220*, *Os02g29340*, and *Os08g43334* had the maximum expression under cytokinin hormone; however no discernible effect was seen in the shoot. Most *OsHSFs* genes in the shoot are up-regulated in response to jasmonate. However, some *OsHSFs* could only be activated by a specific hormone. While other *OsHSFs* displayed virtually minimal expression in response to any hormone stimulation.

**Table 5:** Role of HSF gene family in plant hormones

RiceXpro/RiceFRND (Databases)		Abscisic acid		Gibberellin		Auxin		Brassinosteroid		Cytokinine		Jasmonic acid	
	Locus ID	Root	shoot	Root	shoot	Root	shoot	Root	shoot	Root	shoot	Root	shoot
1	Os01g0571300	3 h-6 h	6 h-12 h	30 min-1 h	equal to control	3 h-6 h	3 h-6 h	3 h	12 h	1 h and 6 h	equal to control	1 h-6 h	3 h- 12 h
2	Os01g0625300	30 min-3 h	less to control	30 min-1 h	equal to control	30 min- 3 h	equal to control	1 h- 6 h	3 h- 12 h	less to control	1 h-12 h	less to control	0 h only
3	Os01g0733200	30 min-6 h	3 h-12 h	equal to control	equal to control	at 3 h only	equal to control	less to control	equal to control	1 h-6 h	equal to control	less to control	equal or less to control
4	Os01g0749300	3 h-6 h	6 h-12 h	only at 3 h	12 h only or equal to control	1 h-6 h	3 h-6 h	6 h	0 h-12 h	6 h	12 h only or equal to control	30 min- 1 h	6 h-12 h
5	Os02g0232000	3 h-6 h	3 h-12 h	30 min-1 h	less to control	equal to control	equal to control	30 min-1 h	equal to control	6 h	equal to control	3 h	12 h
6	Os02g0496100	6 h	equal to control	equal to control	equal to control	6 h	equal to control	equal to control	equal to control	3 h-6 h	equal to control	6 h	6 h- 12 h
7	Os02g0527300	30 min-3 h	1 h-6 h	equal to control	equal to control	equal to control	equal to control	equal to control	equal to control	equal to control	equal to control	1 h-3 h	equal to control
8	Os03g0161900	equal to control	equal to control	3 h	equal to control	30 min-1 h	equal to control	equal to control	equal to control	0 min-1 h	3 h only	15 min-1 h	equal to control
9	Os03g0224700	3 h-6 h	1 h-12 h	equal to control	equal to control	3 h	3 h-12 h	equal to control	equal to control	equal to control	3 h only	1 h-6 h	12 h
10	Os03g0366800	3h-6 h	equal to control	3 h	6–12 h	3h-6 h	equal to control	equal to control	equal to control	equal to control	equal to control	30 min-6 h	equal to control
11	Os03g0745000	3 h-6 h	less to control	equal to control	equal to control	3 h	equal to control	6 h	equal to control	15 min	equal to control	30 min-6 h	equal to control
12	Os03g0795900	3 h	3 h-12 h	equal to control	equal to control	equal to control	equal to control	equal to control	equal to control	6 h	equal to control	0 min	6 h- 12 h
13	Os03g0854500	equal to control	equal to control	equal to control	1 h	equal to control	6 h	equal to control	1 h-3 h	equal to control	12 h	equal to control	6 h
14	Os04g0568700	3 h-6 h	1 h-12 h	equal to control	12 h	30 min-3 h	1 h-12 h	30 min-3 h	equal to control	equal to control	equal to control	1 h-3 h	3 h-12 h
15	Os05g0530400	3 h-6 h	6 h	equal to control	equal to control	1 h-3 h	6 h	Less to control	Less to control	6 h	Less to control	30 min-1 h	3 h-12 h
16	Os06g0553100	3 h-6 h	3 h-12 h	equal to control	equal to control	equal to control	3 h	Less to control	12 h	Less to control	equal to control	3 h	3 h-12 h
17	Os06g0565200	30 min-3 h	Less to control	equal to control	equal to control	30 min-3 h	equal to control	6 h	12 h	1 h	equal to control	equal to control and at 3 h reduced	Less to control
18	Os07g0178600	30 min-6 h	3 h-12 h	equal to control	equal to control	1 h-12 h	equal to control	equal to control	6 h- 12 h	12 h	12 h	3 h	Less to control
19	Os07g0640900	30 min-6 h	3 h-12 h	equal to control	equal to control	1 h-3 h	equal to control	equal to control	6 h	6 h	12 h	3 h	Less to control
20	Os08g0471000	3h-6 h	1h-12 h	less to control	equal to control	3h-6 h	3 h	less to control	less to control	less to control	less to control	1 h and 6 h	3h-12 h
21	Os08g0546800	3 h-6 h	3 h-12 h	3 h	equal to control	3 h-6 h	equal to control	1 h-6 h	Less to control	3 h-6 h	Less to control	1 h and 6 h	3 h-12 h
22	Os09g0455200	equal to control	almost equal to control	less to control	equal to control	less to control	6 h	less to control	equal to control	less to control	12 h	less to control	Less to control
23	Os09g0456800	3 h-6 h	1 h-12 h	less to control	equal to control	3 h-6 h	3 h	less to control	less to control	less to control	less to control	1 h and 6 h	3 h-12 h
24	Os09g0526600	3 h-6 h	1 h-12 h	equal to control	equal to control	3 h	1 h-3 h	less to control	equal to control	less to control	less to control	1 h and 6 h	3 h-12 h
25	Os10g0419300	Increase then decrease	less to control	3 h	less to control	equal to control	equal to control	equal to control	equal to control	less to control	12 h	first increase then decrease	12 h

### Coexpression of *OsHSFs* gene family

**Fig. 13:**
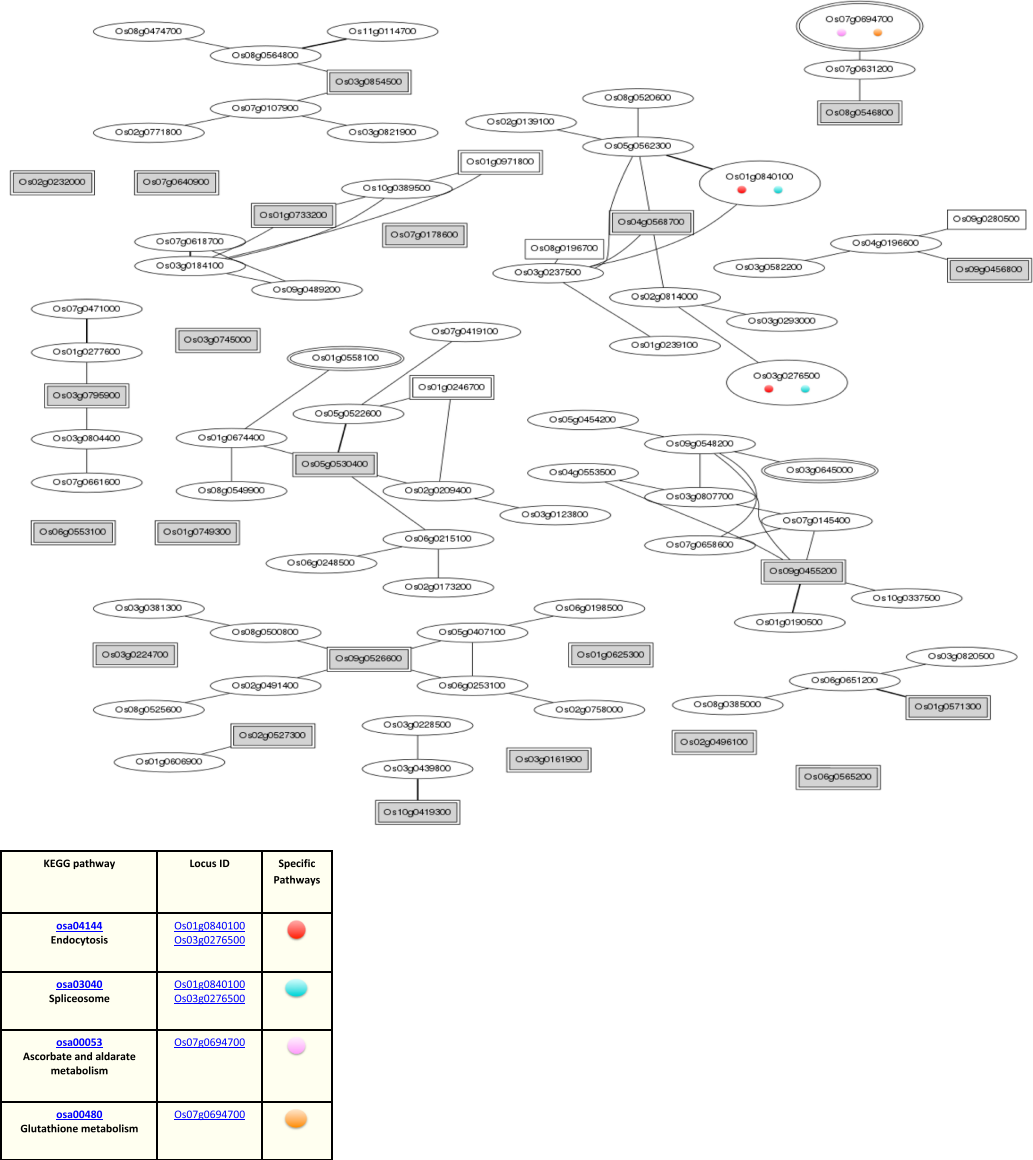
Networking of HSF gene family members [25] triggering multiple genes. Red dots represents the endocytosis process, blue dot for the spliceosome process, pink dot for the ascorbate and aldarate metabolism and yellow dot for glutathione metabolism

**Fig. 14:**
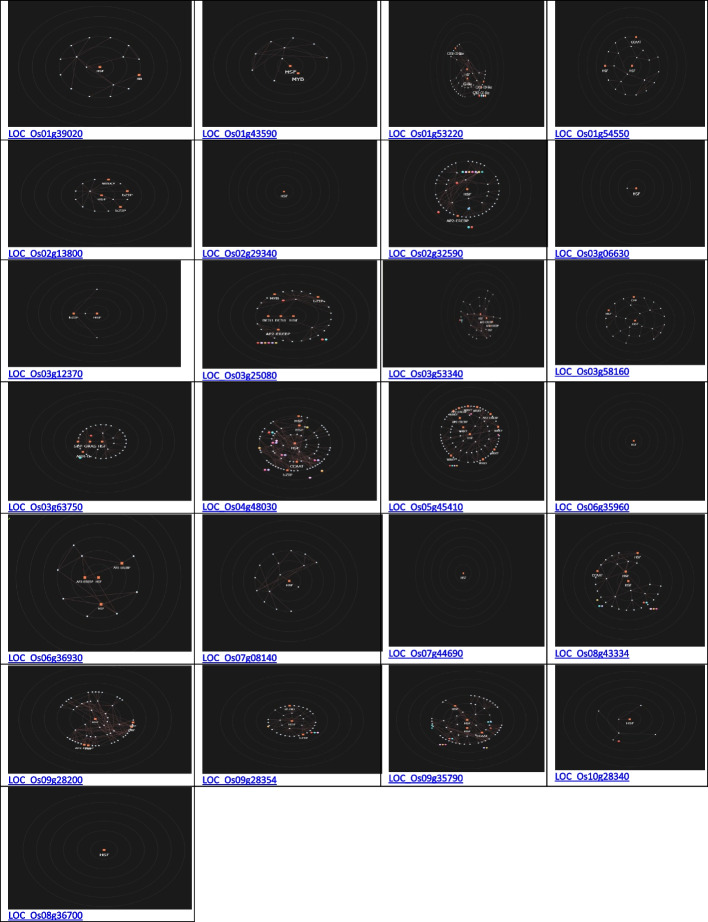
Hyper tree of single guide gene of heat shock factor gene family

According to a hierarchy and mutual rank (MR) value on an ascending MR value, as illustrated in Fig. 13, the HyperTree graphical presentation illustrates the relationships between coexpressed genes. The HyperTree nodes were labeled with transcription factors name. It reveals the association of HSF genes with other TF such as G-2 like, GRAS, RWP, RWK, bZIP, trihelix, WRKY as shown in Fig. 14. As a result, the gene network would show how the 25 HSF genes had overlapping activities and provide valuable information that could be used to better understand the molecular mechanism of rice reproductive evolution.

## Discussion

To provide food security under diverse climate scenario and ever-increasing global populace, it is imperious to comprehend the molecular mechanisms of plants and discover genetic resources related to agricultural productivity. It has been discovered through the sequencing of the crop plants that the number of *OsHSFs* may not be influenced by the genome's size. As an illustration, *Zea mays* (2.4 Gb) has 25 genes, *Oryza sativa* (430 Mb) has 25 HSFs, *A. thaliana* (135 Mb) has 21 HSFs and *Medicago truncatula* (375 Mb) has 15 HSFs, Based on the drafted rice genome sequence [29, 30], Baniwal et al. [31] hypothesised that the rice genome contained 23 genes that encode HSF. *OsHSFs* are essential to plant growth, according to earlier findings. So, using the RiceXPro database, we looked at the specific expression of *OsHSFs* across 12 different developmental stages (Table 4). Many genes had increased expression, which was indicative of how they functioned at various developmental stages. In particular, *Os01g39020*, *Os01g53220*, *Os03g25080*, *Os01g54550*, *Os02g13800* and *Os10g28340* expressed extremely across all the growth stages. This gives significant support for the outcome of our analysis and creates a solid foundation for subsequent research to characterize the functions of *Os01g39020*, *Os01g53220*, *Os01g54550*, *Os02g13800* and *Os10g28340* under different plant hormonal level. Similarly, it has been suggested that *OsHSFs* are crucial for plants to cope with abiotic stresses. According to Kumar et al. (2018), *TaHSFs* A6e modulates wheat's resistance to drought and heat stress during the reproductive phases [32]. By inducing the expression of heat shock proteins (HSPs), Yokotani et al. (2018) shown that *HSFsA2e* restores *Arabidopsis* adaptation to salt and heat stress [33]. The expression of *OsHSF*s was assessed under abiotic stress conditions using microarray analysis. Most of *OsHSF*s family genes displayed stress-specific expression; however some *OsHSF*s exhibited up-regulation under particular stress. According to Jiang (2016), *OsHSFs* improves plant tolerance to heat and salinity stress and escalated sensitivity to the abscisic acid [34]. Similarly in our study, *Os01g39020*, *Os01g53220*, *Os01g54550*, *Os02g13800* and *Os10g28340* genes exhibited highest expression may be used to improved development of plant reproductive organs, leaf diurnal, root diurnal and grains ripening and these genes is also played better performance under different hormonal levels. The study found that there were various transcription factor transcripts under different stress conditions.

In *Arabidopsis*, sunflower and *Medicago truncatula* solely express *HSFA9* gene during seed development [35, 36]. The rice gene *Os03g12370*, which is an Arabidopsis and sunflower homolog, was not expressed during seed development. In our investigation, six *OsHSF* genes had enhanced expression in specific tissue*.* The *Os09g28354* and *Os01g39020* and *Os01g53220* genes have a relationship with reproductive organ tissues, respectively, as well as seed and root tissues. The *Os02g1380* is in root and reproductive organs, *Os05g45410* and *Os01g54550* in leaf, vegetative and ripening, *Os03g58160* in panicle, *Os01g53220* in flower and *Os03g25080* in the pistil has significant affect under stress conditions. Theoretical explanations for HSF A1a/A1b in *Arabidopsis* and HSF A1a in tomato suggest that constitutively produced *OsHSFs* may be crucial for the regulation of stress-induced HSFs genes [37, 38].

It is well known that osmotic stress, salt, cold, and heat all significantly increase the expression of HSF in *Arabidopsis*. In this study, expression profile analysis revealed that *OsHSF* also respond to various abiotic stresses. *Os03g53340, Os07g44690*, *Os01g53220*, *Os01g54550* and *Os02g13800* play major role in the ROS accumulation pathways. Our findings are consistent with those of Wang et al. (2022) [39], who hypothesised that *OsHSFs* would function as sensors for changes in ROS intensity. Among all *OsHSFs*, the gene *Os03g53340* showed the greatest level of expression at both oxidative stress time points. Furthermore, it implies that *Os02g32590* and *Os01g39020* could be involved in the delayed reaction to oxidative stress. It is notable that *Os07g08140* seems to be the least responsive to the stress circumstances whereas *Os03g53340* had the greatest transcript regulation under stress. The co-induction of *OsHSF* genes may provide important details about the pathways that respond to stress. The DNA-binding domain of plant *OsHSFs* genes is divided into two portions by an intron. This intron is located in the same location at each *OsHSF*, however its size varies [40]. The majority of HSF genes only contain one intron in the DBD, and rice is not an exception to this rule (Fig. 6). Besides, it was revealed that the rice HSFs gene is not intron-less, contrary to the general finding that roughly 20% of rice genes are intron-less [19, 41]. Intron-less genes have been found in several rice transcription factors such as MADS box [42], C2H2 zinc finger [43], bZIP [44], SAUR [45] and F-box [46] gene families. Alternative splicing may occur and vary according to environmental stresses and at certain developmental stages. The *Oryza* 10% and *Arabidopsis* 11.6% genes exhibited alternatively spliced across numerous tissues [41, 47]. It is hypothesized that the evolution of a gene family is significantly influenced by the increase or decrease in exon number. As a result, the quantity and distribution of introns and exons in *OsHSF* genes were examined. Our findings showed that, with the exception of *Os08g43334*, *Os03g12370*, *Os09g35790*, and *Os03g06630*, all *OsHSF* genes had one intron and two exons (Fig. 6). Furthermore, exon and intron length and positions varied significantly between various subclasses as they were highly conserved within the same subclasses. It is reported that the improvement of translational efficiency through the promotion of gene expression by intron transcription initiation, increased mRNA accumulation [48].

According to Xie et al. 2019, the *OsHSF* family of genes exhibits a co-expression pattern under various abiotic stimuli in *Arabidopsis* [49]. Our study indicates that *OsHSF* TF regulate multiple mechanisms in rice. During co-expression analysis of selected *OsHSF* gene(s), it was found that the *OsHSF* genes trigger the C2H2 type zinc finger proteins that enhance plant drought resistance through activating the expression of related targeted genes and increasing the levels of osmotic regulations [50]. The *OsHSF* genes also co-expressed with Golden 2-like family genes (G2-like) that have been characterized by regulating the formation of chloroplasts during the transition and early maturing phases Fig. 14 [51].

The CCAAT-binding complex (CBC), which regulates primary and secondary metabolism, development, stress reactions, and pathogenicity in fungi and plants, is activated by the *OsHSFs*. The CBC is normally composed of heterotrimeric core subunits. [52]. Moreover, the sequence-specific DNA-binding TF known as "growth regulating factors" (GRFs) regulate numerous aspects of plant growth and development [53, 54]. The BZR1/BES1 and *OsHSFs* play a crucial role in BR signaling and also act as a regulator in multi-signal-regulated plant growth and development events by directly networking with other key proteins or genes [42, 55].

In the co-expression analysis, the AP2/EREBP genes were also triggered that play indispensable roles in root initiation and growth of rice plant [56]. The basic leucine zipper (bZIP) family control key processes in all eukaryotes. In plants, bZIPs with *OsHSFs* regulates many central developmental and physiological processes like photomorphogenesis, energy homeostasis, leaf formation, seed development under biotic and biotic stresses. The rice drought stress is caused by TF that are encoded by the bZIP genes. By modifying amino acid metabolism, *OsMYB55* and *OsHSFs* co-expressed in rice promote vegetative development and increase grain production under high temperature circumstances [57]. Systematic investigation of the rice TF family gene reveals that they are up-regulated under heat stress and contribute in a multiplicative way to the *OsHSF* genes [58].

When plant is growing, auxin stimulates the cell wall and also influences root formation [59].

The root nodules (RN) symbiosis is dependent on two GRAS domain transcription factors known as the nodulation signaling pathway (NSP1 and NSP2). Their rice homologs, *OsNSP1* and *OsNSP2*, effectively reversed the RN symbiosis-defective phenotypes of the mutants of the corresponding genes in the model legume *Lotus japonicas* [60, 61]. Through cell differentiation*, OsHSFs* and the RWP-RK domain regulate the development of female gametophytes. This is good attribute to identify the early maturing rice genotypes which flower under high temperature [62].

Co-expression of HSF and WRKY TF, which respond to biotic and abiotic stresses, controls plant growth and development. It is still unclear how WRKY TFs regulate plant height in rice and react to drought stress at the molecular level. In rice, the majority of the WRKY genes show variable responses towards cold, heat, PEG and salinity stresses [63]. Recently, the *HSFA2e* gene has been annotated to confer thermo-tolerance in transgenic *Arabidopsis* plants [64].

In the field, plants are subjected to a variety of stresses; hence it’s crucial to develop crop types that are resilient to a variety of stress conditions. The ability of *OsHSFs* to respond to stress can be used to create transgenic rice plants that are tolerant to abiotic stress [65].

## Conclusion

Comprehensive *in-silico* investigation, including phylogenetic analysis, gene structure and conserved motif analysis, chromosomal location, evolutionary analysis, and *OsHSF* expression profile, was carried out to better understand the function of 25 *OsHSF* genes. According to expression profiling, *Os03g53340*, *Os01g54550*, *Os02g13800*, and *Os01g39020* are the key heat shock regulators (HSR) in rice, and *Os03g53340* is crucial for the early activation of the heat shock protein gene under heat stress. These findings laid the foundation for developmental processes and responses to various stresses using various functional validation processes, such as overexpression, knockout via CRISPR/Cas9 systems, etc. The role of *OsHSFs* in the abiotic stress response pathway was initiated not only in heat shock but also in other abiotic stresses. This information can be used to produce stress-tolerant rice cultivars suitable under changing climate conditions.

### Supplementary Information

**Additional file 1. **

**Additional file 2. **

**Additional file 3. **

## Data Availability

All data generated or analysed during this study are included in public repository, [PERSISTENT WEB LINK OR ACCESSION NUMBER TO DATASETS]”. 1. The datasets generated during the current study are available in the public repositories and analysed using online tools/softwares. The National Centre for Biotechnology Information (NCBI) https://www.ncbi.nlm.nih.gov/, the Database of Rice Transcription Factors (DRTF) http://planttfdb.gao-lab. org/index.php?sp = Osj, MSU Rice Genome Annotation Project Database http://rice.uga.edu/, Plant Genome Database (PlantGDB) https://www.plantgdb.org/, The RAP-DB website's BLAST online tool http://rice.uga.edu/analyses_search_blast.shtml GENSCAN http://hollywood.mit.edu/GENSCAN.html The Simple Modular Architecture Research Tool (SMART) http://smart.embl-heidelberg.de/. Clustal Omega https://www.ebi.ac.uk/Tools/msa/clustalo/. Multiple Em for Motif Elicitation (MEME Suite version 5.5.0) https://meme-suite.org/meme/tools/meme. Gene Structure Display Server (GSDS) http://gsds.gao-lab.org/, Tbtool https://github.com/CJ-Chen/TBtools /releases, AlphaFold https://alphafold.ebi.ac.uk/, RiceXPro https://ricexpro.dna.affrc.go.jp/, Ricefrnd https://ricefrend.dna.affrc.go.jp/single-guide-gene.html.
