# Supplementary material for: In-silico analysis of heat shock transcription factor (OsHSF) gene family in rice (Oryza sativa L.)
Source: BMC Plant Biol. 2023 Aug 17;23:395. doi: 10.1186/s12870-023-04399-1 (PMC10433574; doi:10.1186/s12870-023-04399-1)
Supplement: Supplementary file 1 — Additional file 1. [file 12870_2023_4399_MOESM1_ESM.docx]

**S1.**

The protein sequences of HSF family used in this study

>LOC_Os01g39020

MLKPQTPRARRAAHPNSHMASSSSSSSLCRLLIPRPTTRRFSGGGGEGGMAAAAPVKREV

KPEAGEGWGGGDLGVVPPPPRPMEGLGEAGPAPFVAKTYEMVADAATDAVVSWGPGGSGA

SFVVWDPHALAAGVLPRFFKHANFSSFVRQLNTYGFRKVTPDRWEFANEAFLAGQKHLLK

NIKRRRVSKPLVDSQLRNKASVVFGQPEAPGEVVSLKRDRAALRAEVIMLKQQYNACKSQ

LIAMEEMVRNIERRQQQTIGFFAKVLTNPAFVQQVLLNYVNKNGLRGAAKRQRLMENEEQ

HADSPLNKGMEAASVMEADVSPGSTGCGTVGKVETTPMCNFQNIENMCDDVWEELDALPE

TGMEQEEKAGIGSFDVEEFVGRPCGWVDDCPYLVEPMQFVEH*

>LOC_Os01g43590

MDGLHTELALGLIGCCGGDGQQQTAPFVAKTYQMVCDPRTDALVRWGRDNNSFVVVDPAA

FSQLLLPCFFKHGNFSSFVRQLNTYVSIIQSPAPGFRKVHPDRWEFAHESFLRGQTHLLP

RIVRRKKRGEGGGGGGGASCSFGGGAGEHQVAAAAASVGMSGEEEDAAEDVLAKEAALFE

EVQRLRHEQTAIGEELARMSQRLQATERRPDQLMSFLAKLADDPNAVTGHLLEQAAERKR

RRQHLPSHEPTVCPLPPAPPPQPPQPLLALAGAAAMDGTYWWTTEHHHHHHHQMKPMTVL

PSLEPPTASCGVHQVPELGGGGVMGLTTDGEAKVEPPFPFCLLGQAFF*

>LOC_Os01g53220

MMGGECKVHQLQAAGDGGPGAVAPFVAKTFHMVSDPSTNAVVRWGGAGNTFLVLDPAAFS

DFLLPSYFKHRNFASFVRQLNTYGFRKVDPDRWEFAHESFLRGQAQLLPRIVRKKKKGGA

APGCRELCEEGEEVRGTIEAVQRLREEQRGMEEELQAMDQRLRAAESRPGQMMAFLAKLA

DEPGVVLRAMLAKKEELAAAGNNGSDPCKRRRIGADTGRGGVATGGDAAEMAQSRGTVPF

PFSVLGQVFY*

>LOC_Os01g54550

MEGGGGGGSLPPFLSKTYEMVDDPSTDAVVGWTPAGTSFVVANQPEFCRDLLPKYFKHNN

FSSFVRQLNTYGFRKVDPEQWEFANEDFIKGQRHRLKNIHRRKPIFSHSSHSQGAGPLTD

NERKDYEEEIERLKSDNAALSSELQNNTLKKLNMEKRMQALEEKLFVVEDQQRSLISYVR

EIVKAPGFLSSFVQQQDHHRKKRRLPIPISFHEDANTQENQIMPCDLTNSPAQTFYRESF

DKMESSLNSLENFLREASEEFGNDISYDDGVPGPSSTVVLTELHSPGESDPRVSSPPTRM

RTSSAGAGDSHSSRDVAESTSCAESPPIPQMHSRVDTRAKVSEIDVNSEPAVTETGPSRD

QPAEEPPAVTPGANDGFWQQFLTEQPGSSDAHQEAQSERRDGGNKVDEMKSGDRQHLWWG

KRNVEQITEKLGLLTSTEKT*

>LOC_Os02g13800

MTTTTAEGGGGVAPFVAKTYRMVDDPATDGVIAWGRDSNSFVVADPFAFSQTLLPAHFKH

SNFSSFVRQLNTYGFRKVDPDRWEFAHVSFLRGQTHLLRRIVRRSSGGGGAKRKEEAGGC

GGGGEAAAGDVDEESAVVALEVARLRREQREIEGRVAAMWRRVQETERRPKQMLAFLVKV

VGDPQVLRRLVDRDNTNAAASNADDSAVHHQVKRPRLLLDSSSTTTTHGDRHLVTAAADG

FYAGGCGPEAAAAAAFVPDDAVDFTGLYTGGDGFGNAVVDAGVDYPPAYAFPVVDSGY*

>LOC_Os02g29340

MEVAAGARGGGAGGGGGGPAPFLLKTYEMVDDPSTDAVVSWSDASDASFVVWNHPEFAAR

LLPAYFKHSNFSSFIRQLNTYGFRKIDPERWEFANEYFIKGQKHLLKNIHRRKPIHSHSH

PPGALPDNERAIFEDEIERLSREKSNLQADLWKSKQQQSGTMNQIEDLERRVLGMEQRQT

KMIAFLQQASKNPQFVNKLVKMAEASSIFTDAFNKKRRLPGLDYSIENTETTSFYDDHSS

TSKQETGNLLNQHFSDKLRLGLCPAMTESNIITLSTQSSNEDNRSPHGKHPECDMMGREC

LPLVPQMMELSDTGTSICPSKSSCFAPPISDEGLLTCHLSLTLASCSMDVDKSQGLNANG

TTIDNPTEAATATMEKDDTIDRSFDDNQKKSADSRTADATTPRADARVASEAPAAPAAVV

NDKFWEQFLTERPGCSETEEASSGLRTDTSREQMENRQAYDHSRNDREDVEQLKL*

>LOC_Os02g32590

MDHNTDPPPTTMVDAAAALLLEPKLEGYDDDGGGEPLQPAPFVSPLDQLMQPPRPLEALL

QGPQLPPFLSKTYDLVCEPELDGVISWGHAGNSFVVWDPSAFARDVLPHHFKHNNFSSFV

RQLNTYGFRKVHADRWEFAHEDFLRHSKHLLKKIVRRRSSPTQQSGLQPGSSGESGLDPE

LNTLRREKSALLQEVTRLKQEHLQTIEQMSTLNQRLESAEDRQKQMVSFLAKLLQNPTFL

RQLKMHRQQKEIDSTRVKRKFLKHVPHGNIDSGESSSQHTGESNLDFSPTSLDLPATHSD

ILDLQNFLLEDGDLNLAMLPENIGLDGIEAPDDIGALVQGFDTQEELELGSGVELLEIPP

ASGPRGQDPTIGRSKGKNVLSPGLDATSSEADCLGSFSDNMGMLSDSMLQTAGKLMDADD

DERIWGVDASSALQSSCSGTSQQAYGSLVSDPYLMEMANKPEKFWELDFQALDDGDLQLD

KCVIDDPALQQQRGNMNS*

>LOC_Os03g06630

MEKMMPGMVKEEWPPSSPEEGEAPRPMEGLHEVGPPPFLTKTFDLVADPATDGVVSWGRA

GSSFVVWDPHVFAAVFLPRFFKHNNFSSFVRQLNTYFLVRTNYLNKRSHFYSLRFQGFRK

IDPDRWEFANDGFLRGQRHLLKMIKRRRPLSYLPGSQQALGTCLEVGQFGLDEEIDRLKR

DKNILLAEVVKLRHKQQSTKANMRAMEERLQHAEQKQVQMMGFLARAMQNPDFFHQLIHQ

QDKMKGLEDTFSKKRTRSIDIVPFLNPGEVSQGDQLESTLLFDPRPFAELNDEPAKSELE

NLALNIQGLGKGKQDVNRTRNQPRNQASNETELTDDFWEELLNEGARDDAGIPGMERRRP

RYVDALAQKLGYLSNSSQK*

>LOC_Os03g12370

MGSKKRSPQHPAAAAPPPAVGGGGGGEVSGDGGASTANGPVVPKPSEVAPFLTKVYDMVS

DPATDNVISWAEGGGSFVIWDSHAFERDLHRHFKHSNFTSFIRQLNTYGFRKVHPDRWEW

ANEGFIMGQKHLLKTIKRRKKSSQESPSEIQKAPVKTAPGTENIEIGKYGGLEKEVETLK

RDKALLMQQLVDLRHYQQTSNLEVQNLIERLQVMEQNQQQMMALLAIVVQNPSFLNQLVQ

QQQQQRRSNWWSPDGSKKRRFHALEQGPVTDQETSGRGAHIVEYLPPVPETSGQVNPVEG

AICSANSQPVPSPAVATPMDMQTSNVADTLGSSEEPFADNSTLHEWDDNDMQLLFDDNLD

PILPPFENDGQMGPPLSVQDYDFPQLEQDCLMEAQYNSNNPQYADVITEA*

>LOC_Os03g25080 or >LOC_Os03g25120

MAFLVERCGGEMVVSMERSHGRSTTTAAAVTAAPAPFLSKTYQLVDDPSTDDVVSWGEDE

ATFVVWRPPEFARDLLPNYFKHNNFSSFVRQLNTYGFRKIVADRWEFANEFFRKGAKHLL

SEIHRRKSSSCSQPQPPPPFPMHQHYPLSLFSPPTTPRSPPVGAAAAAAYHFQEEYCSSP

ADYAGGGGDLLAALSEDNRQLRRRNSLLLSELAHMRKLYNDIIYFLQNHVEPVAPPPLAA

ATSCRLVELGPSTTERRRCAASPSGDNDDDAAVRLFGVRLDDDHGKKRRVQLVQEDEGDE

QGSEG*

>LOC_Os03g53340

MNPLRVIVKEEELDFAAAAAAAAAGEGSPSSWAVGVMDLPRPMEGLGEAGPPPFLCKTYE

VVDDPGTDTVISWGFAGNSFVVWDANAFAAVLLPRYFKHSNFSSFVRQLNTYGFRKVDPD

RWEFANEGFLRGKKELLKTIKRRRPPPSSPPSSSSSSSSSQHQQQPAAACLEVGQFGRDG

VVNRLQRDKSVLIAEVVKLRQEQQTTRAQMQAMEERISAAEQKQQQMTVFLARAMKNPGF

LQMLVDRQAGQHGARNRVLEDALSKKRRRPIEYLLTRNGETCAAGESAAMLAADGVAEPD

GDTTPRGDGGGGGGGDTESFWMQLLSLGLEEKQREDGVAGGVQESNSGGADVDNDEEDDD

DDVDVLVQSIYHLSPK*

>LOC_Os03g58160

MNYRVVNPVKVESGPSTGVANGQPPRPMDGLADGGPPPFLTKTYDMVDDPTTDAVVSWSA

TNNSFVVWDPHLFGNVLLPRYFKHNNFSSFVRQLNTYGFRKVDPDKWEFANEGFLRGQKH

LLKSIKRRKPPNSSPSQQSLGSFLEVGHFGYEGEIDQLKRDKHLLMAEVVKLRQEQQNTK

SDLQAMEQKLQGTEQKQQHMMAFLSRVMHNPEFIRQLFSQSEMRKELEEFVSKKRRRRID

QGPELDSMGTGSSPEQVSQVMFEPHDPVDSLFNGVPSDLESSSVEANGGKAQQDVASSSS

EHGKIKPSNGELNEDFWEDLLHEGGLDEDTRNPAIDDMNLLSQKMGYLNSSSTKSPQ*

>LOC_Os03g63750

MEAAVAAAAAAAGAVTTAVAPPPGAAVSNGVATAPPPFLMKTYEMVDDPATDAVVSWGPG

NNSFVVWNTPEFARDLLPKYFKHSNFSSFVRQLNTYGFRKVDPDRWEFANEGFLRGQKHL

LKTINRRKPTHGNNQVQQPQLPAAPVPACVEVGKFGMEEEIEMLKRDKNVLMQELVRLRQ

QQQTTDHQLQTLGKRLQGMEQRQQQMMSFLAKAMHSPGFLAQFVQQNENSRRRIVASNKK

RRLPKQDGSLDSESASLDGQIVKYQPMINEAAKAMLRKILKLDSSHRFESMGNSDNFLLE

NYMPNGQGLDSSSSTRNSGVTLAEVPANSGLPYVATSSGLSAICSTSTPQIQCPVVLDNG

IPKEVPNMSAVPSVPKAVAPGPTDINILEFPDLQDIVAEENVDIPGGGFEMPGPEGVFSL

PEEGDDSVPIETDEILYNDDTQKLPAIIDSFWEQFLVASPLSVDNDEVDSGVLDQKETQQ

GNGWTKAENMANLTEQMGLLSSHHTG*

>LOC_Os04g48030

MASPAAGTPPFLTKTYAMVEDPSTDETISWNDSGTAFVVWRPAEFARDLLPKHFKHSNFS

SFVRQLNTYGFKKVVADRWEFANDCFRRGEKHLLGGIQRRKGSGTGGAGAAPAGGIPTAI

PISSPPTSSGGEPAVSSSPPRGAAGIAAGVSGAVAELEEENARLRRENARLARELARARR

VCDGVRRLVSRYDHDHGGGEEEAGEGDVKPMLFGVAIGGKRSREENGEDEEEEEEEGADE

DGEDDEVEEDDEERERHAARRVPVREGKVRRTTELSDLDVLALSVRAAAAARPGGASRDR

KSSVS*

>LOC_Os05g45410

MESSNLGGGGGGGGGGGPPPFLIKTYEMVEDAATNHVVSWGPGGASFVVWNPLDFSRDLL

PKYFKHNNFSSFIRQLNTYGFRKIDPERWEFANEDFIRGHTHLLKNIHRRKPVHSHSLQN

QINGPLAESERRELEEEINRLKYEKSILVADLQRQNQQQYVINWQMQAMEGRLVAMEQRQ

KNIVASLCEMLQRRGGAVSSSLLESDHFSKKRRVPKMDLFVDDCAAGEEQKVFQFQGIGT

DAPAMPPVLPVTNGEAFDRVELSLVSLEKLFQRANDACTAAEEMYSHGHGGTEPSTAICP

EEMNTAPMETGIDLQLPASLHPSSPNTGNAHLHLSTELTESPGFVQSPELPMAEIREDIH

VTRYPTQADVNSEIASSTDTSQDGTSETEASHGPTNDVFWERFLTETPRSCLDESERQES

PKDDVKAELGCNGFHHREKVDQITEQMGHLASAEQTLHT*

>LOC_Os06g35960

MAAAAGGGAAPFVWKTYRMVEDPGTDGVIGWGKGNNSFVVADPFVFSQTLLPAHFKHNNF

SSFVRQLNTYGFRKVDPDRWEFAHASFLRGQTHLLRNIVRRGSAAAGGGGGGGGGKRRDA

SADGGGGGGDEDMTMVATEVVRLKQEQRTIDDRVAAMWRRVQETERRPKQMLAFLLKVVG

DRDKLHRLVGGGGNGNGAATAAAADNGFADAARAGCGEKRARLLLDGDNTGAFGPDAVDF

AGFYTGADMFPDVAVDAAAAAAGGSAGCSFAFGVDSGY*

>LOC_Os06g36930

MDYSTVKQEEVEVVVLDGEEEAAAAAAPVPLPAAMGVGAAVAPFLVKTFEMVEDPATDAV

VSWGGAARNSFVVWDPHAFAAGLLPLHFKHANFSSFLRQLNTYGFRKVSADRWEFANEDF

LGGQRHLLANIRRRRRGAGTGSTTPRAVNCGGGGGEGEVERLRRDKEALARELARLRRQQ

QEARAQLLDMERRVRGTERRQEQCTEFLARALRSPDVLDNIARRHAAAVERKKRRMLAAA

ADDDGLTFEALALAAAADTSHSTGGAVTTDMIWYELLGEEQAEIDIEVDQLVASASAAAD

TASEAEPWEEMGEEEVQELVQQIDCLASPSS*

>LOC_Os07g08140

MDDPMLNAVKEEESHGDGGGLEVVAGEDGAAAVAAGVAPRPMEGLHDAGPPPFLTKTYDM

VDDAGTDAAVSWSATSNSFVVWDPHAFATVLLPRFFKHNNFSSFVRQLNTYGFRKVDPDR

WEFANENFLRGQRHLLKNIKRRKPPSHTASNQQSLGPYLEVGHFGYDAEIDRLKRDKQLL

MAEVVKLRQEQQNTKANLKAMEDRLQGTEQRQQQMMAFLARVMKNPEFLKQLMSQNEMRK

ELQDAISKKRRRRIDQGPEVDDVGTSSSIEQESPALFDPQESVEFLIDGIPSDLENSAMD

AGGLVEPQDFDVGASEQQQIGPQGELNDNFWEELLNEGLVGEENDNPVVEDDMNVLSEKM

GYLNSNGPTAGE*

>LOC_Os07g44690

MAFLVERCGEMVVSMEMGPHGGGGAAAGKPVPAPFLTKTYQLVDDPCTDHIVSWGEDDTT

FVVWRPPEFARDLLPNYFKHNNFSSFVRQLNTYGFRKIVADRWEFANEFFRKGAKHLLAE

IHRRKSSQPPPPPMPHQPYHHHHHLNPFSLPPPPPAYHHHHLIQEEPATTAHCTVAGDGG

EGGDFLAALSEDNRQLRRRNSLLLSELAHMKKLYNDIIYFLQNHVAPVTTTTTTPSSTAM

AAAQHHLPAAASCRLMELDSPDHSPPPPPPKTPATDGGDTVKLFGVSLHGRKKRAHRDDD

DGVHDQGSEV*

>LOC_Os08g36700

MEWEEESEAARQKAAAASASVVPAPFLTKTYQLVDDPATDHVVSWEDDDGGESASSFVVW

RPPEFARDILPNYFKHSNFSSFVRQLNTYGFRKVVPERWEFANEFFRKGEKQLLCEIHRR

KSAAATWPPFPPPPPPFFAPRHFAAGAFFRHGDGMLHGRLGALVTTTERRHWFESAALPV

APSSRLLSQLGPVIAPARRAAATPEEEALMQENHRLLRGNAALVQELAHMRKLYSDIIYF

VQNHVRPVAPSPAAAAALHGLGVLRPPPAGGKAPASEVRGASGRSATSSSSLTVAEDQPT

LLALRLPRTTEKIINEVSGGNGGGSTKLFGVHLSSADEQTSSGASRKRSPPQEQPPTSPA

PKRTLVVEHSELRLSIVSPP*

>LOC_Os08g43334

MKGSRLAVKESCLPMTMPMPETFAQYSNPLRSTRAYGQLCRVPGRRRHACVDGWGQDRAR

TRGDDGQRRYAQCRGGWGDIWRWGGYITCGSPRETGMVNGAPSPPPPSPMVMSFGPLDSP

WVKQPDTTVYPGQICAAAGGGGGMADQTAAAVVVGGGAAATMGEPSPPPPAPAAEAAGVG

VGQQQRTVPTPFLTKTYQLVDDPAVDDVISWNDDGSTFVVWRPAEFARDLLPKYFKHNNF

SSFVRQLNTYGFRKIVPDRWEFANDCFRRGERRLLCEIHRRKVTPPAPAATTAAVAAAIP

MALPVTTTRDGSPVLSGEEQVISSSSSPEPPLVLPQAPSGSGSGGVASGDVGDENERLRR

ENAQLARELSQMRKLCNNILLLMSKYASTQQLDAANASSAAGNNNNNNCSGESAEAATPL

PLPAVLDLMPSCPGAASAAAPVSDNEEGMMSAKLFGVSIGRKRMRHDGGGDDDHAATVKA

EPMDGRPHGKDEQSAETQAWPIYRPRPVYQPIRACNGYEYDRAGSDQDECGAKPSNQDKK

DLLRRWVVLISESLQLHGRQVVGAVQEWRRDRRGHRRPPAGHVQLRTCVNNLLVTGAGTL

DAGREYETNSLPRPRR*

>LOC_Os09g28200

MERCGSWSDCEAAAAAAQKAVPAPFLTKTYQLVDDPATDHIVSWGDDRVSTFVVWRPPEF

ARDILPNYFKHNNFSSFVRQLNTYGFRKVVPERWEFANEFFRKGEKQLLTEIHRRKTSSA

STASPSPPPFFAPPHFPLFHHPGVAAAQHHHAFVGDDGVVAAHGIGMPFPQPHWREPNLP

VATRLLALGGPAPSPSSAEAGGAGRAATAAVLMEENERLRRSNTALLQELAHMRKLYNDI

IYFVQNHVRPVAPSPAAAAFLQGLGMQARKKPAAANVLNNSGGSTTSSSSLTIAEEPSPP

PQQQHLAGEKSGGEAGNSSAARSSAPTKLFGVHLSAAPCGAGSKRASSPEEHPPTSPATK

PRLVLECDDLSLTVAPSSSSQQQLSAASSPTSTS*

>LOC_Os09g28354

MAAAEAAAAVGKQQQKGGGGRGGGGGGPAPFLTKTNQMVEESATDEVISWGKEGRSFVVW

KPVEFARDLLPLHFKHCNFSSFVRQLNTYGFRKVVPDRWEFANGNFRRGEQGLLSGIRRR

KATTPQSSKSCGSGVNVAFPPPLPPLPPEPSATTSSGNDRSSSSASSPPRADITSENEQL

RKDNQTLTMELARARRHCEELLGFLSRFLDVRQLDLRLLMQEDMRAAAGGVGGEQRVQEH

AREEKCVKLFGVLLDDTHGAATRKRARCEEAAASERPIKMIRIGEPWVSVPSSGPARCGG

DN*

>LOC_Os09g35790

MAEQGAGEADAGGGEPPPAAVMTAAAEALAGQRSLPTPFLTKTYQLVEDPAVDDVISWNE

DGSTFVVWRPAEFARDLLPKYFKHNNFSSFVRQLNTYGFRKIVPDRWEFANDCFRRGEKR

LLCDIHRRKVVAAAAAAPPPPSPGMATAAAAVASGAVTVAAAPIPMALPVTRAGSPAHSS

EEQVLSSNSGSGEEHRQASGSGSAPGGGGGGSASGGDMGEENERLRRENARLTRELGHMK

KLCNNILLLMSKYAATQHVEGSAGISSIANCSGESSEAVPPPPPLPPAILDLMPSCPALA

TAAAAAGLAIDGEPDPSARLFGVSIGLKRTRDDAAAAADEDGGGEDQAEHGGADVKPEAA

DPHPAGGGGGSSTEASPESHPWPIYRPTPMYHAVRPTCNGPDRAGSDQDGSSSSQTMGPG

EFDDLQKMMVVQQSNFVMHWGRSECGSGVRGFGW*

>LOC_Os10g28340

MDPAAAGIVKEEMLESQQQQRQEDGGAAPRPMEGLHEVGPPPFLTKTYDLVEDPATDGVV

SWSRAGNSFVVWDPHVFADLLLPRLFKHNNFSSFVRQLNTYGFRKVDPDRWEFANEGFLR

GQRHLLKTIKRRKPPSNAPPSQQQSLTSCLEVGEFGFEEEIDRLKRDKNILITEVVKLRQ

EQQATKDHVKAMEDRLRAAEQKQVQMMGFLARAMRNPEFFQQLAQQKEKRKELEDAISKK

RRRPIDNVPFYDPGETSQTEQLDSPYLFDSGVLNELSEPGIPELENLAVNIQDLGKGKVD

EERQNQTNGQAELGDDFWAELLVEDFTGKEEQSELDGKIDGIDELAQQLGYLSSTSPK*
