# Supplementary material for: In-silico analysis of heat shock transcription factor (OsHSF) gene family in rice (Oryza sativa L.)
Source: BMC Plant Biol. 2023 Aug 17;23:395. doi: 10.1186/s12870-023-04399-1 (PMC10433574; doi:10.1186/s12870-023-04399-1)
Supplement: Supplementary file 2 — Additional file 2. [file 12870_2023_4399_MOESM2_ESM.docx]

**S2.**

The nucleotide sequences of HSF family used in this study

>LOC_Os01g39020

ATGCTCAAGCCGCAGACCCCGCGCGCCCGCCGCGCCGCCCACCCGAACTCCCACATGGCC

TCCTCCTCCTCCTCCTCCTCCCTCTGCCGCCTCCTCATCCCGCGCCCGACCACCCGCCGC

TTCTCCGGCGGCGGCGGCGAGGGCGGCATGGCCGCCGCCGCCCCGGTGAAGCGCGAGGTG

AAGCCGGAGGCCGGCGAGGGCTGGGGCGGCGGCGACCTCGGCGTGGTGCCGCCGCCGCCG

CGCCCGATGGAGGGCCTCGGCGAGGCCGGCCCGGCCCCGTTCGTGGCCAAGACCTACGAG

ATGGTGGCCGACGCCGCCACCGACGCCGTGGTGTCCTGGGGCCCGGGCGGCTCCGGCGCC

TCCTTCGTGGTGTGGGACCCGCACGCCCTCGCCGCCGGCGTGCTCCCGCGCTTCTTCAAG

CACGCCAACTTCTCCTCCTTCGTGCGCCAGCTCAACACCTACGGCTTCCGCAAGGTGACC

CCGGACCGCTGGGAGTTCGCCAACGAGGCCTTCCTCGCCGGCCAGAAGCACCTCCTCAAG

AACATCAAGCGCCGCCGCGTGTCCAAGCCGCTCGTGGACTCCCAGCTCCGCAACAAGGCC

TCCGTGGTGTTCGGCCAGCCGGAGGCCCCGGGCGAGGTGGTGTCCCTCAAGCGCGACCGC

GCCGCCCTCCGCGCCGAGGTGATCATGCTCAAGCAGCAGTACAACGCCTGCAAGTCCCAG

CTCATCGCCATGGAGGAGATGGTGCGCAACATCGAGCGCCGCCAGCAGCAGACCATCGGC

TTCTTCGCCAAGGTGCTCACCAACCCGGCCTTCGTGCAGCAGGTGCTCCTCAACTACGTG

AACAAGAACGGCCTCCGCGGCGCCGCCAAGCGCCAGCGCCTCATGGAGAACGAGGAGCAG

CACGCCGACTCCCCGCTCAACAAGGGCATGGAGGCCGCCTCCGTGATGGAGGCCGACGTG

TCCCCGGGCTCCACCGGCTGCGGCACCGTGGGCAAGGTGGAGACCACCCCGATGTGCAAC

TTCCAGAACATCGAGAACATGTGCGACGACGTGTGGGAGGAGCTCGACGCCCTCCCGGAG

ACCGGCATGGAGCAGGAGGAGAAGGCCGGCATCGGCTCCTTCGACGTGGAGGAGTTCGTG

GGCCGCCCGTGCGGCTGGGTGGACGACTGCCCGTACCTCGTGGAGCCGATGCAGTTCGTG

GAGCACTGA

>LOC_Os01g43590

ATGGACGGCCTCCACACCGAGCTCGCCCTCGGCCTCATCGGCTGCTGCGGCGGCGACGGC

CAGCAGCAGACCGCCCCGTTCGTGGCCAAGACCTACCAGATGGTGTGCGACCCGCGCACC

GACGCCCTCGTGCGCTGGGGCCGCGACAACAACTCCTTCGTGGTGGTGGACCCGGCCGCC

TTCTCCCAGCTCCTCCTCCCGTGCTTCTTCAAGCACGGCAACTTCTCCTCCTTCGTGCGC

CAGCTCAACACCTACGTGTCCATCATCCAGTCCCCGGCCCCGGGCTTCCGCAAGGTGCAC

CCGGACCGCTGGGAGTTCGCCCACGAGTCCTTCCTCCGCGGCCAGACCCACCTCCTCCCG

CGCATCGTGCGCCGCAAGAAGCGCGGCGAGGGCGGCGGCGGCGGCGGCGGCGCCTCCTGC

TCCTTCGGCGGCGGCGCCGGCGAGCACCAGGTGGCCGCCGCCGCCGCCTCCGTGGGCATG

TCCGGCGAGGAGGAGGACGCCGCCGAGGACGTGCTCGCCAAGGAGGCCGCCCTCTTCGAG

GAGGTGCAGCGCCTCCGCCACGAGCAGACCGCCATCGGCGAGGAGCTCGCCCGCATGTCC

CAGCGCCTCCAGGCCACCGAGCGCCGCCCGGACCAGCTCATGTCCTTCCTCGCCAAGCTC

GCCGACGACCCGAACGCCGTGACCGGCCACCTCCTCGAGCAGGCCGCCGAGCGCAAGCGC

CGCCGCCAGCACCTCCCGTCCCACGAGCCGACCGTGTGCCCGCTCCCGCCGGCCCCGCCG

CCGCAGCCGCCGCAGCCGCTCCTCGCCCTCGCCGGCGCCGCCGCCATGGACGGCACCTAC

TGGTGGACCACCGAGCACCACCACCACCACCACCACCAGATGAAGCCGATGACCGTGCTC

CCGTCCCTCGAGCCGCCGACCGCCTCCTGCGGCGTGCACCAGGTGCCGGAGCTCGGCGGC

GGCGGCGTGATGGGCCTCACCACCGACGGCGAGGCCAAGGTGGAGCCGCCGTTCCCGTTC

TGCCTCCTCGGCCAGGCCTTCTTCTGA

>LOC_Os01g53220

ATGATGGGCGGCGAGTGCAAGGTGCACCAGCTCCAGGCCGCCGGCGACGGCGGCCCGGGC

GCCGTGGCCCCGTTCGTGGCCAAGACCTTCCACATGGTGTCCGACCCGTCCACCAACGCC

GTGGTGCGCTGGGGCGGCGCCGGCAACACCTTCCTCGTGCTCGACCCGGCCGCCTTCTCC

GACTTCCTCCTCCCGTCCTACTTCAAGCACCGCAACTTCGCCTCCTTCGTGCGCCAGCTC

AACACCTACGGCTTCCGCAAGGTGGACCCGGACCGCTGGGAGTTCGCCCACGAGTCCTTC

CTCCGCGGCCAGGCCCAGCTCCTCCCGCGCATCGTGCGCAAGAAGAAGAAGGGCGGCGCC

GCCCCGGGCTGCCGCGAGCTCTGCGAGGAGGGCGAGGAGGTGCGCGGCACCATCGAGGCC

GTGCAGCGCCTCCGCGAGGAGCAGCGCGGCATGGAGGAGGAGCTCCAGGCCATGGACCAG

CGCCTCCGCGCCGCCGAGTCCCGCCCGGGCCAGATGATGGCCTTCCTCGCCAAGCTCGCC

GACGAGCCGGGCGTGGTGCTCCGCGCCATGCTCGCCAAGAAGGAGGAGCTCGCCGCCGCC

GGCAACAACGGCTCCGACCCGTGCAAGCGCCGCCGCATCGGCGCCGACACCGGCCGCGGC

GGCGTGGCCACCGGCGGCGACGCCGCCGAGATGGCCCAGTCCCGCGGCACCGTGCCGTTC

CCGTTCTCCGTGCTCGGCCAGGTGTTCTACTGA

>LOC_Os01g54550

ATGGAGGGCGGCGGCGGCGGCGGCTCCCTCCCGCCGTTCCTCTCCAAGACCTACGAGATG

GTGGACGACCCGTCCACCGACGCCGTGGTGGGCTGGACCCCGGCCGGCACCTCCTTCGTG

GTGGCCAACCAGCCGGAGTTCTGCCGCGACCTCCTCCCGAAGTACTTCAAGCACAACAAC

TTCTCCTCCTTCGTGCGCCAGCTCAACACCTACGGCTTCCGCAAGGTGGACCCGGAGCAG

TGGGAGTTCGCCAACGAGGACTTCATCAAGGGCCAGCGCCACCGCCTCAAGAACATCCAC

CGCCGCAAGCCGATCTTCTCCCACTCCTCCCACTCCCAGGGCGCCGGCCCGCTCACCGAC

AACGAGCGCAAGGACTACGAGGAGGAGATCGAGCGCCTCAAGTCCGACAACGCCGCCCTC

TCCTCCGAGCTCCAGAACAACACCCTCAAGAAGCTCAACATGGAGAAGCGCATGCAGGCC

CTCGAGGAGAAGCTCTTCGTGGTGGAGGACCAGCAGCGCTCCCTCATCTCCTACGTGCGC

GAGATCGTGAAGGCCCCGGGCTTCCTCTCCTCCTTCGTGCAGCAGCAGGACCACCACCGC

AAGAAGCGCCGCCTCCCGATCCCGATCTCCTTCCACGAGGACGCCAACACCCAGGAGAAC

CAGATCATGCCGTGCGACCTCACCAACTCCCCGGCCCAGACCTTCTACCGCGAGTCCTTC

GACAAGATGGAGTCCTCCCTCAACTCCCTCGAGAACTTCCTCCGCGAGGCCTCCGAGGAG

TTCGGCAACGACATCTCCTACGACGACGGCGTGCCGGGCCCGTCCTCCACCGTGGTGCTC

ACCGAGCTCCACTCCCCGGGCGAGTCCGACCCGCGCGTGTCCTCCCCGCCGACCCGCATG

CGCACCTCCTCCGCCGGCGCCGGCGACTCCCACTCCTCCCGCGACGTGGCCGAGTCCACC

TCCTGCGCCGAGTCCCCGCCGATCCCGCAGATGCACTCCCGCGTGGACACCCGCGCCAAG

GTGTCCGAGATCGACGTGAACTCCGAGCCGGCCGTGACCGAGACCGGCCCGTCCCGCGAC

CAGCCGGCCGAGGAGCCGCCGGCCGTGACCCCGGGCGCCAACGACGGCTTCTGGCAGCAG

TTCCTCACCGAGCAGCCGGGCTCCTCCGACGCCCACCAGGAGGCCCAGTCCGAGCGCCGC

GACGGCGGCAACAAGGTGGACGAGATGAAGTCCGGCGACCGCCAGCACCTCTGGTGGGGC

AAGCGCAACGTGGAGCAGATCACCGAGAAGCTCGGCCTCCTCACCTCCACCGAGAAGACC

TGA

>LOC_Os02g13800

ATGACCACCACCACCGCCGAGGGCGGCGGCGGCGTGGCCCCGTTCGTGGCCAAGACCTAC

CGCATGGTGGACGACCCGGCCACCGACGGCGTGATCGCCTGGGGCCGCGACTCCAACTCC

TTCGTGGTGGCCGACCCGTTCGCCTTCTCCCAGACCCTCCTCCCGGCCCACTTCAAGCAC

TCCAACTTCTCCTCCTTCGTGCGCCAGCTCAACACCTACGGCTTCCGCAAGGTGGACCCG

GACCGCTGGGAGTTCGCCCACGTGTCCTTCCTCCGCGGCCAGACCCACCTCCTCCGCCGC

ATCGTGCGCCGCTCCTCCGGCGGCGGCGGCGCCAAGCGCAAGGAGGAGGCCGGCGGCTGC

GGCGGCGGCGGCGAGGCCGCCGCCGGCGACGTGGACGAGGAGTCCGCCGTGGTGGCCCTC

GAGGTGGCCCGCCTCCGCCGCGAGCAGCGCGAGATCGAGGGCCGCGTGGCCGCCATGTGG

CGCCGCGTGCAGGAGACCGAGCGCCGCCCGAAGCAGATGCTCGCCTTCCTCGTGAAGGTG

GTGGGCGACCCGCAGGTGCTCCGCCGCCTCGTGGACCGCGACAACACCAACGCCGCCGCC

TCCAACGCCGACGACTCCGCCGTGCACCACCAGGTGAAGCGCCCGCGCCTCCTCCTCGAC

TCCTCCTCCACCACCACCACCCACGGCGACCGCCACCTCGTGACCGCCGCCGCCGACGGC

TTCTACGCCGGCGGCTGCGGCCCGGAGGCCGCCGCCGCCGCCGCCTTCGTGCCGGACGAC

GCCGTGGACTTCACCGGCCTCTACACCGGCGGCGACGGCTTCGGCAACGCCGTGGTGGAC

GCCGGCGTGGACTACCCGCCGGCCTACGCCTTCCCGGTGGTGGACTCCGGCTACTGA

>LOC_Os02g29340

ATGGAGGTGGCCGCCGGCGCCCGCGGCGGCGGCGCCGGCGGCGGCGGCGGCGGCCCGGCC

CCGTTCCTCCTCAAGACCTACGAGATGGTGGACGACCCGTCCACCGACGCCGTGGTGTCC

TGGTCCGACGCCTCCGACGCCTCCTTCGTGGTGTGGAACCACCCGGAGTTCGCCGCCCGC

CTCCTCCCGGCCTACTTCAAGCACTCCAACTTCTCCTCCTTCATCCGCCAGCTCAACACC

TACGGCTTCCGCAAGATCGACCCGGAGCGCTGGGAGTTCGCCAACGAGTACTTCATCAAG

GGCCAGAAGCACCTCCTCAAGAACATCCACCGCCGCAAGCCGATCCACTCCCACTCCCAC

CCGCCGGGCGCCCTCCCGGACAACGAGCGCGCCATCTTCGAGGACGAGATCGAGCGCCTC

TCCCGCGAGAAGTCCAACCTCCAGGCCGACCTCTGGAAGTCCAAGCAGCAGCAGTCCGGC

ACCATGAACCAGATCGAGGACCTCGAGCGCCGCGTGCTCGGCATGGAGCAGCGCCAGACC

AAGATGATCGCCTTCCTCCAGCAGGCCTCCAAGAACCCGCAGTTCGTGAACAAGCTCGTG

AAGATGGCCGAGGCCTCCTCCATCTTCACCGACGCCTTCAACAAGAAGCGCCGCCTCCCG

GGCCTCGACTACTCCATCGAGAACACCGAGACCACCTCCTTCTACGACGACCACTCCTCC

ACCTCCAAGCAGGAGACCGGCAACCTCCTCAACCAGCACTTCTCCGACAAGCTCCGCCTC

GGCCTCTGCCCGGCCATGACCGAGTCCAACATCATCACCCTCTCCACCCAGTCCTCCAAC

GAGGACAACCGCTCCCCGCACGGCAAGCACCCGGAGTGCGACATGATGGGCCGCGAGTGC

CTCCCGCTCGTGCCGCAGATGATGGAGCTCTCCGACACCGGCACCTCCATCTGCCCGTCC

AAGTCCTCCTGCTTCGCCCCGCCGATCTCCGACGAGGGCCTCCTCACCTGCCACCTCTCC

CTCACCCTCGCCTCCTGCTCCATGGACGTGGACAAGTCCCAGGGCCTCAACGCCAACGGC

ACCACCATCGACAACCCGACCGAGGCCGCCACCGCCACCATGGAGAAGGACGACACCATC

GACCGCTCCTTCGACGACAACCAGAAGAAGTCCGCCGACTCCCGCACCGCCGACGCCACC

ACCCCGCGCGCCGACGCCCGCGTGGCCTCCGAGGCCCCGGCCGCCCCGGCCGCCGTGGTG

AACGACAAGTTCTGGGAGCAGTTCCTCACCGAGCGCCCGGGCTGCTCCGAGACCGAGGAG

GCCTCCTCCGGCCTCCGCACCGACACCTCCCGCGAGCAGATGGAGAACCGCCAGGCCTAC

GACCACTCCCGCAACGACCGCGAGGACGTGGAGCAGCTCAAGCTCTGA

>LOC_Os02g32590

ATGGACCACAACACCGACCCGCCGCCGACCACCATGGTGGACGCCGCCGCCGCCCTCCTC

CTCGAGCCGAAGCTCGAGGGCTACGACGACGACGGCGGCGGCGAGCCGCTCCAGCCGGCC

CCGTTCGTGTCCCCGCTCGACCAGCTCATGCAGCCGCCGCGCCCGCTCGAGGCCCTCCTC

CAGGGCCCGCAGCTCCCGCCGTTCCTCTCCAAGACCTACGACCTCGTGTGCGAGCCGGAG

CTCGACGGCGTGATCTCCTGGGGCCACGCCGGCAACTCCTTCGTGGTGTGGGACCCGTCC

GCCTTCGCCCGCGACGTGCTCCCGCACCACTTCAAGCACAACAACTTCTCCTCCTTCGTG

CGCCAGCTCAACACCTACGGCTTCCGCAAGGTGCACGCCGACCGCTGGGAGTTCGCCCAC

GAGGACTTCCTCCGCCACTCCAAGCACCTCCTCAAGAAGATCGTGCGCCGCCGCTCCTCC

CCGACCCAGCAGTCCGGCCTCCAGCCGGGCTCCTCCGGCGAGTCCGGCCTCGACCCGGAG

CTCAACACCCTCCGCCGCGAGAAGTCCGCCCTCCTCCAGGAGGTGACCCGCCTCAAGCAG

GAGCACCTCCAGACCATCGAGCAGATGTCCACCCTCAACCAGCGCCTCGAGTCCGCCGAG

GACCGCCAGAAGCAGATGGTGTCCTTCCTCGCCAAGCTCCTCCAGAACCCGACCTTCCTC

CGCCAGCTCAAGATGCACCGCCAGCAGAAGGAGATCGACTCCACCCGCGTGAAGCGCAAG

TTCCTCAAGCACGTGCCGCACGGCAACATCGACTCCGGCGAGTCCTCCTCCCAGCACACC

GGCGAGTCCAACCTCGACTTCTCCCCGACCTCCCTCGACCTCCCGGCCACCCACTCCGAC

ATCCTCGACCTCCAGAACTTCCTCCTCGAGGACGGCGACCTCAACCTCGCCATGCTCCCG

GAGAACATCGGCCTCGACGGCATCGAGGCCCCGGACGACATCGGCGCCCTCGTGCAGGGC

TTCGACACCCAGGAGGAGCTCGAGCTCGGCTCCGGCGTGGAGCTCCTCGAGATCCCGCCG

GCCTCCGGCCCGCGCGGCCAGGACCCGACCATCGGCCGCTCCAAGGGCAAGAACGTGCTC

TCCCCGGGCCTCGACGCCACCTCCTCCGAGGCCGACTGCCTCGGCTCCTTCTCCGACAAC

ATGGGCATGCTCTCCGACTCCATGCTCCAGACCGCCGGCAAGCTCATGGACGCCGACGAC

GACGAGCGCATCTGGGGCGTGGACGCCTCCTCCGCCCTCCAGTCCTCCTGCTCCGGCACC

TCCCAGCAGGCCTACGGCTCCCTCGTGTCCGACCCGTACCTCATGGAGATGGCCAACAAG

CCGGAGAAGTTCTGGGAGCTCGACTTCCAGGCCCTCGACGACGGCGACCTCCAGCTCGAC

AAGTGCGTGATCGACGACCCGGCCCTCCAGCAGCAGCGCGGCAACATGAACTCCTGA

>LOC_Os03g06630

ATGGAGAAGATGATGCCGGGCATGGTGAAGGAGGAGTGGCCGCCGTCCTCCCCGGAGGAG

GGCGAGGCCCCGCGCCCGATGGAGGGCCTCCACGAGGTGGGCCCGCCGCCGTTCCTCACC

AAGACCTTCGACCTCGTGGCCGACCCGGCCACCGACGGCGTGGTGTCCTGGGGCCGCGCC

GGCTCCTCCTTCGTGGTGTGGGACCCGCACGTGTTCGCCGCCGTGTTCCTCCCGCGCTTC

TTCAAGCACAACAACTTCTCCTCCTTCGTGCGCCAGCTCAACACCTACTTCCTCGTGCGC

ACCAACTACCTCAACAAGCGCTCCCACTTCTACTCCCTCCGCTTCCAGGGCTTCCGCAAG

ATCGACCCGGACCGCTGGGAGTTCGCCAACGACGGCTTCCTCCGCGGCCAGCGCCACCTC

CTCAAGATGATCAAGCGCCGCCGCCCGCTCTCCTACCTCCCGGGCTCCCAGCAGGCCCTC

GGCACCTGCCTCGAGGTGGGCCAGTTCGGCCTCGACGAGGAGATCGACCGCCTCAAGCGC

GACAAGAACATCCTCCTCGCCGAGGTGGTGAAGCTCCGCCACAAGCAGCAGTCCACCAAG

GCCAACATGCGCGCCATGGAGGAGCGCCTCCAGCACGCCGAGCAGAAGCAGGTGCAGATG

ATGGGCTTCCTCGCCCGCGCCATGCAGAACCCGGACTTCTTCCACCAGCTCATCCACCAG

CAGGACAAGATGAAGGGCCTCGAGGACACCTTCTCCAAGAAGCGCACCCGCTCCATCGAC

ATCGTGCCGTTCCTCAACCCGGGCGAGGTGTCCCAGGGCGACCAGCTCGAGTCCACCCTC

CTCTTCGACCCGCGCCCGTTCGCCGAGCTCAACGACGAGCCGGCCAAGTCCGAGCTCGAG

AACCTCGCCCTCAACATCCAGGGCCTCGGCAAGGGCAAGCAGGACGTGAACCGCACCCGC

AACCAGCCGCGCAACCAGGCCTCCAACGAGACCGAGCTCACCGACGACTTCTGGGAGGAG

CTCCTCAACGAGGGCGCCCGCGACGACGCCGGCATCCCGGGCATGGAGCGCCGCCGCCCG

CGCTACGTGGACGCCCTCGCCCAGAAGCTCGGCTACCTCTCCAACTCCTCCCAGAAGTGA

>LOC_Os03g12370

ATGGGCTCCAAGAAGCGCTCCCCGCAGCACCCGGCCGCCGCCGCCCCGCCGCCGGCCGTG

GGCGGCGGCGGCGGCGGCGAGGTGTCCGGCGACGGCGGCGCCTCCACCGCCAACGGCCCG

GTGGTGCCGAAGCCGTCCGAGGTGGCCCCGTTCCTCACCAAGGTGTACGACATGGTGTCC

GACCCGGCCACCGACAACGTGATCTCCTGGGCCGAGGGCGGCGGCTCCTTCGTGATCTGG

GACTCCCACGCCTTCGAGCGCGACCTCCACCGCCACTTCAAGCACTCCAACTTCACCTCC

TTCATCCGCCAGCTCAACACCTACGGCTTCCGCAAGGTGCACCCGGACCGCTGGGAGTGG

GCCAACGAGGGCTTCATCATGGGCCAGAAGCACCTCCTCAAGACCATCAAGCGCCGCAAG

AAGTCCTCCCAGGAGTCCCCGTCCGAGATCCAGAAGGCCCCGGTGAAGACCGCCCCGGGC

ACCGAGAACATCGAGATCGGCAAGTACGGCGGCCTCGAGAAGGAGGTGGAGACCCTCAAG

CGCGACAAGGCCCTCCTCATGCAGCAGCTCGTGGACCTCCGCCACTACCAGCAGACCTCC

AACCTCGAGGTGCAGAACCTCATCGAGCGCCTCCAGGTGATGGAGCAGAACCAGCAGCAG

ATGATGGCCCTCCTCGCCATCGTGGTGCAGAACCCGTCCTTCCTCAACCAGCTCGTGCAG

CAGCAGCAGCAGCAGCGCCGCTCCAACTGGTGGTCCCCGGACGGCTCCAAGAAGCGCCGC

TTCCACGCCCTCGAGCAGGGCCCGGTGACCGACCAGGAGACCTCCGGCCGCGGCGCCCAC

ATCGTGGAGTACCTCCCGCCGGTGCCGGAGACCTCCGGCCAGGTGAACCCGGTGGAGGGC

GCCATCTGCTCCGCCAACTCCCAGCCGGTGCCGTCCCCGGCCGTGGCCACCCCGATGGAC

ATGCAGACCTCCAACGTGGCCGACACCCTCGGCTCCTCCGAGGAGCCGTTCGCCGACAAC

TCCACCCTCCACGAGTGGGACGACAACGACATGCAGCTCCTCTTCGACGACAACCTCGAC

CCGATCCTCCCGCCGTTCGAGAACGACGGCCAGATGGGCCCGCCGCTCTCCGTGCAGGAC

TACGACTTCCCGCAGCTCGAGCAGGACTGCCTCATGGAGGCCCAGTACAACTCCAACAAC

CCGCAGTACGCCGACGTGATCACCGAGGCCTGA

>LOC_Os03g25080 or >LOC_Os03g25120

ATGGCCTTCCTCGTGGAGCGCTGCGGCGGCGAGATGGTGGTGTCCATGGAGCGCTCCCAC

GGCCGCTCCACCACCACCGCCGCCGCCGTGACCGCCGCCCCGGCCCCGTTCCTCTCCAAG

ACCTACCAGCTCGTGGACGACCCGTCCACCGACGACGTGGTGTCCTGGGGCGAGGACGAG

GCCACCTTCGTGGTGTGGCGCCCGCCGGAGTTCGCCCGCGACCTCCTCCCGAACTACTTC

AAGCACAACAACTTCTCCTCCTTCGTGCGCCAGCTCAACACCTACGGCTTCCGCAAGATC

GTGGCCGACCGCTGGGAGTTCGCCAACGAGTTCTTCCGCAAGGGCGCCAAGCACCTCCTC

TCCGAGATCCACCGCCGCAAGTCCTCCTCCTGCTCCCAGCCGCAGCCGCCGCCGCCGTTC

CCGATGCACCAGCACTACCCGCTCTCCCTCTTCTCCCCGCCGACCACCCCGCGCTCCCCG

CCGGTGGGCGCCGCCGCCGCCGCCGCCTACCACTTCCAGGAGGAGTACTGCTCCTCCCCG

GCCGACTACGCCGGCGGCGGCGGCGACCTCCTCGCCGCCCTCTCCGAGGACAACCGCCAG

CTCCGCCGCCGCAACTCCCTCCTCCTCTCCGAGCTCGCCCACATGCGCAAGCTCTACAAC

GACATCATCTACTTCCTCCAGAACCACGTGGAGCCGGTGGCCCCGCCGCCGCTCGCCGCC

GCCACCTCCTGCCGCCTCGTGGAGCTCGGCCCGTCCACCACCGAGCGCCGCCGCTGCGCC

GCCTCCCCGTCCGGCGACAACGACGACGACGCCGCCGTGCGCCTCTTCGGCGTGCGCCTC

GACGACGACCACGGCAAGAAGCGCCGCGTGCAGCTCGTGCAGGAGGACGAGGGCGACGAG

CAGGGCTCCGAGGGCTGA

>LOC_Os03g53340

ATGAACCCGCTCCGCGTGATCGTGAAGGAGGAGGAGCTCGACTTCGCCGCCGCCGCCGCC

GCCGCCGCCGCCGGCGAGGGCTCCCCGTCCTCCTGGGCCGTGGGCGTGATGGACCTCCCG

CGCCCGATGGAGGGCCTCGGCGAGGCCGGCCCGCCGCCGTTCCTCTGCAAGACCTACGAG

GTGGTGGACGACCCGGGCACCGACACCGTGATCTCCTGGGGCTTCGCCGGCAACTCCTTC

GTGGTGTGGGACGCCAACGCCTTCGCCGCCGTGCTCCTCCCGCGCTACTTCAAGCACTCC

AACTTCTCCTCCTTCGTGCGCCAGCTCAACACCTACGGCTTCCGCAAGGTGGACCCGGAC

CGCTGGGAGTTCGCCAACGAGGGCTTCCTCCGCGGCAAGAAGGAGCTCCTCAAGACCATC

AAGCGCCGCCGCCCGCCGCCGTCCTCCCCGCCGTCCTCCTCCTCCTCCTCCTCCTCCTCC

CAGCACCAGCAGCAGCCGGCCGCCGCCTGCCTCGAGGTGGGCCAGTTCGGCCGCGACGGC

GTGGTGAACCGCCTCCAGCGCGACAAGTCCGTGCTCATCGCCGAGGTGGTGAAGCTCCGC

CAGGAGCAGCAGACCACCCGCGCCCAGATGCAGGCCATGGAGGAGCGCATCTCCGCCGCC

GAGCAGAAGCAGCAGCAGATGACCGTGTTCCTCGCCCGCGCCATGAAGAACCCGGGCTTC

CTCCAGATGCTCGTGGACCGCCAGGCCGGCCAGCACGGCGCCCGCAACCGCGTGCTCGAG

GACGCCCTCTCCAAGAAGCGCCGCCGCCCGATCGAGTACCTCCTCACCCGCAACGGCGAG

ACCTGCGCCGCCGGCGAGTCCGCCGCCATGCTCGCCGCCGACGGCGTGGCCGAGCCGGAC

GGCGACACCACCCCGCGCGGCGACGGCGGCGGCGGCGGCGGCGGCGACACCGAGTCCTTC

TGGATGCAGCTCCTCTCCCTCGGCCTCGAGGAGAAGCAGCGCGAGGACGGCGTGGCCGGC

GGCGTGCAGGAGTCCAACTCCGGCGGCGCCGACGTGGACAACGACGAGGAGGACGACGAC

GACGACGTGGACGTGCTCGTGCAGTCCATCTACCACCTCTCCCCGAAGTGA

>LOC_Os03g58160

ATGAACTACCGCGTGGTGAACCCGGTGAAGGTGGAGTCCGGCCCGTCCACCGGCGTGGCC

AACGGCCAGCCGCCGCGCCCGATGGACGGCCTCGCCGACGGCGGCCCGCCGCCGTTCCTC

ACCAAGACCTACGACATGGTGGACGACCCGACCACCGACGCCGTGGTGTCCTGGTCCGCC

ACCAACAACTCCTTCGTGGTGTGGGACCCGCACCTCTTCGGCAACGTGCTCCTCCCGCGC

TACTTCAAGCACAACAACTTCTCCTCCTTCGTGCGCCAGCTCAACACCTACGGCTTCCGC

AAGGTGGACCCGGACAAGTGGGAGTTCGCCAACGAGGGCTTCCTCCGCGGCCAGAAGCAC

CTCCTCAAGTCCATCAAGCGCCGCAAGCCGCCGAACTCCTCCCCGTCCCAGCAGTCCCTC

GGCTCCTTCCTCGAGGTGGGCCACTTCGGCTACGAGGGCGAGATCGACCAGCTCAAGCGC

GACAAGCACCTCCTCATGGCCGAGGTGGTGAAGCTCCGCCAGGAGCAGCAGAACACCAAG

TCCGACCTCCAGGCCATGGAGCAGAAGCTCCAGGGCACCGAGCAGAAGCAGCAGCACATG

ATGGCCTTCCTCTCCCGCGTGATGCACAACCCGGAGTTCATCCGCCAGCTCTTCTCCCAG

TCCGAGATGCGCAAGGAGCTCGAGGAGTTCGTGTCCAAGAAGCGCCGCCGCCGCATCGAC

CAGGGCCCGGAGCTCGACTCCATGGGCACCGGCTCCTCCCCGGAGCAGGTGTCCCAGGTG

ATGTTCGAGCCGCACGACCCGGTGGACTCCCTCTTCAACGGCGTGCCGTCCGACCTCGAG

TCCTCCTCCGTGGAGGCCAACGGCGGCAAGGCCCAGCAGGACGTGGCCTCCTCCTCCTCC

GAGCACGGCAAGATCAAGCCGTCCAACGGCGAGCTCAACGAGGACTTCTGGGAGGACCTC

CTCCACGAGGGCGGCCTCGACGAGGACACCCGCAACCCGGCCATCGACGACATGAACCTC

CTCTCCCAGAAGATGGGCTACCTCAACTCCTCCTCCACCAAGTCCCCGCAGTGA

>LOC_Os03g63750

ATGGAGGCCGCCGTGGCCGCCGCCGCCGCCGCCGCCGGCGCCGTGACCACCGCCGTGGCC

CCGCCGCCGGGCGCCGCCGTGTCCAACGGCGTGGCCACCGCCCCGCCGCCGTTCCTCATG

AAGACCTACGAGATGGTGGACGACCCGGCCACCGACGCCGTGGTGTCCTGGGGCCCGGGC

AACAACTCCTTCGTGGTGTGGAACACCCCGGAGTTCGCCCGCGACCTCCTCCCGAAGTAC

TTCAAGCACTCCAACTTCTCCTCCTTCGTGCGCCAGCTCAACACCTACGGCTTCCGCAAG

GTGGACCCGGACCGCTGGGAGTTCGCCAACGAGGGCTTCCTCCGCGGCCAGAAGCACCTC

CTCAAGACCATCAACCGCCGCAAGCCGACCCACGGCAACAACCAGGTGCAGCAGCCGCAG

CTCCCGGCCGCCCCGGTGCCGGCCTGCGTGGAGGTGGGCAAGTTCGGCATGGAGGAGGAG

ATCGAGATGCTCAAGCGCGACAAGAACGTGCTCATGCAGGAGCTCGTGCGCCTCCGCCAG

CAGCAGCAGACCACCGACCACCAGCTCCAGACCCTCGGCAAGCGCCTCCAGGGCATGGAG

CAGCGCCAGCAGCAGATGATGTCCTTCCTCGCCAAGGCCATGCACTCCCCGGGCTTCCTC

GCCCAGTTCGTGCAGCAGAACGAGAACTCCCGCCGCCGCATCGTGGCCTCCAACAAGAAG

CGCCGCCTCCCGAAGCAGGACGGCTCCCTCGACTCCGAGTCCGCCTCCCTCGACGGCCAG

ATCGTGAAGTACCAGCCGATGATCAACGAGGCCGCCAAGGCCATGCTCCGCAAGATCCTC

AAGCTCGACTCCTCCCACCGCTTCGAGTCCATGGGCAACTCCGACAACTTCCTCCTCGAG

AACTACATGCCGAACGGCCAGGGCCTCGACTCCTCCTCCTCCACCCGCAACTCCGGCGTG

ACCCTCGCCGAGGTGCCGGCCAACTCCGGCCTCCCGTACGTGGCCACCTCCTCCGGCCTC

TCCGCCATCTGCTCCACCTCCACCCCGCAGATCCAGTGCCCGGTGGTGCTCGACAACGGC

ATCCCGAAGGAGGTGCCGAACATGTCCGCCGTGCCGTCCGTGCCGAAGGCCGTGGCCCCG

GGCCCGACCGACATCAACATCCTCGAGTTCCCGGACCTCCAGGACATCGTGGCCGAGGAG

AACGTGGACATCCCGGGCGGCGGCTTCGAGATGCCGGGCCCGGAGGGCGTGTTCTCCCTC

CCGGAGGAGGGCGACGACTCCGTGCCGATCGAGACCGACGAGATCCTCTACAACGACGAC

ACCCAGAAGCTCCCGGCCATCATCGACTCCTTCTGGGAGCAGTTCCTCGTGGCCTCCCCG

CTCTCCGTGGACAACGACGAGGTGGACTCCGGCGTGCTCGACCAGAAGGAGACCCAGCAG

GGCAACGGCTGGACCAAGGCCGAGAACATGGCCAACCTCACCGAGCAGATGGGCCTCCTC

TCCTCCCACCACACCGGCTGA

>LOC_Os04g48030

ATGGCCTCCCCGGCCGCCGGCACCCCGCCGTTCCTCACCAAGACCTACGCCATGGTGGAG

GACCCGTCCACCGACGAGACCATCTCCTGGAACGACTCCGGCACCGCCTTCGTGGTGTGG

CGCCCGGCCGAGTTCGCCCGCGACCTCCTCCCGAAGCACTTCAAGCACTCCAACTTCTCC

TCCTTCGTGCGCCAGCTCAACACCTACGGCTTCAAGAAGGTGGTGGCCGACCGCTGGGAG

TTCGCCAACGACTGCTTCCGCCGCGGCGAGAAGCACCTCCTCGGCGGCATCCAGCGCCGC

AAGGGCTCCGGCACCGGCGGCGCCGGCGCCGCCCCGGCCGGCGGCATCCCGACCGCCATC

CCGATCTCCTCCCCGCCGACCTCCTCCGGCGGCGAGCCGGCCGTGTCCTCCTCCCCGCCG

CGCGGCGCCGCCGGCATCGCCGCCGGCGTGTCCGGCGCCGTGGCCGAGCTCGAGGAGGAG

AACGCCCGCCTCCGCCGCGAGAACGCCCGCCTCGCCCGCGAGCTCGCCCGCGCCCGCCGC

GTGTGCGACGGCGTGCGCCGCCTCGTGTCCCGCTACGACCACGACCACGGCGGCGGCGAG

GAGGAGGCCGGCGAGGGCGACGTGAAGCCGATGCTCTTCGGCGTGGCCATCGGCGGCAAG

CGCTCCCGCGAGGAGAACGGCGAGGACGAGGAGGAGGAGGAGGAGGAGGGCGCCGACGAG

GACGGCGAGGACGACGAGGTGGAGGAGGACGACGAGGAGCGCGAGCGCCACGCCGCCCGC

CGCGTGCCGGTGCGCGAGGGCAAGGTGCGCCGCACCACCGAGCTCTCCGACCTCGACGTG

CTCGCCCTCTCCGTGCGCGCCGCCGCCGCCGCCCGCCCGGGCGGCGCCTCCCGCGACCGC

AAGTCCTCCGTGTCCTGA

>LOC_Os05g45410

ATGGAGTCCTCCAACCTCGGCGGCGGCGGCGGCGGCGGCGGCGGCGGCGGCCCGCCGCCG

TTCCTCATCAAGACCTACGAGATGGTGGAGGACGCCGCCACCAACCACGTGGTGTCCTGG

GGCCCGGGCGGCGCCTCCTTCGTGGTGTGGAACCCGCTCGACTTCTCCCGCGACCTCCTC

CCGAAGTACTTCAAGCACAACAACTTCTCCTCCTTCATCCGCCAGCTCAACACCTACGGC

TTCCGCAAGATCGACCCGGAGCGCTGGGAGTTCGCCAACGAGGACTTCATCCGCGGCCAC

ACCCACCTCCTCAAGAACATCCACCGCCGCAAGCCGGTGCACTCCCACTCCCTCCAGAAC

CAGATCAACGGCCCGCTCGCCGAGTCCGAGCGCCGCGAGCTCGAGGAGGAGATCAACCGC

CTCAAGTACGAGAAGTCCATCCTCGTGGCCGACCTCCAGCGCCAGAACCAGCAGCAGTAC

GTGATCAACTGGCAGATGCAGGCCATGGAGGGCCGCCTCGTGGCCATGGAGCAGCGCCAG

AAGAACATCGTGGCCTCCCTCTGCGAGATGCTCCAGCGCCGCGGCGGCGCCGTGTCCTCC

TCCCTCCTCGAGTCCGACCACTTCTCCAAGAAGCGCCGCGTGCCGAAGATGGACCTCTTC

GTGGACGACTGCGCCGCCGGCGAGGAGCAGAAGGTGTTCCAGTTCCAGGGCATCGGCACC

GACGCCCCGGCCATGCCGCCGGTGCTCCCGGTGACCAACGGCGAGGCCTTCGACCGCGTG

GAGCTCTCCCTCGTGTCCCTCGAGAAGCTCTTCCAGCGCGCCAACGACGCCTGCACCGCC

GCCGAGGAGATGTACTCCCACGGCCACGGCGGCACCGAGCCGTCCACCGCCATCTGCCCG

GAGGAGATGAACACCGCCCCGATGGAGACCGGCATCGACCTCCAGCTCCCGGCCTCCCTC

CACCCGTCCTCCCCGAACACCGGCAACGCCCACCTCCACCTCTCCACCGAGCTCACCGAG

TCCCCGGGCTTCGTGCAGTCCCCGGAGCTCCCGATGGCCGAGATCCGCGAGGACATCCAC

GTGACCCGCTACCCGACCCAGGCCGACGTGAACTCCGAGATCGCCTCCTCCACCGACACC

TCCCAGGACGGCACCTCCGAGACCGAGGCCTCCCACGGCCCGACCAACGACGTGTTCTGG

GAGCGCTTCCTCACCGAGACCCCGCGCTCCTGCCTCGACGAGTCCGAGCGCCAGGAGTCC

CCGAAGGACGACGTGAAGGCCGAGCTCGGCTGCAACGGCTTCCACCACCGCGAGAAGGTG

GACCAGATCACCGAGCAGATGGGCCACCTCGCCTCCGCCGAGCAGACCCTCCACACCTGA

>LOC_Os06g35960

ATGGCCGCCGCCGCCGGCGGCGGCGCCGCCCCGTTCGTGTGGAAGACCTACCGCATGGTG

GAGGACCCGGGCACCGACGGCGTGATCGGCTGGGGCAAGGGCAACAACTCCTTCGTGGTG

GCCGACCCGTTCGTGTTCTCCCAGACCCTCCTCCCGGCCCACTTCAAGCACAACAACTTC

TCCTCCTTCGTGCGCCAGCTCAACACCTACGGCTTCCGCAAGGTGGACCCGGACCGCTGG

GAGTTCGCCCACGCCTCCTTCCTCCGCGGCCAGACCCACCTCCTCCGCAACATCGTGCGC

CGCGGCTCCGCCGCCGCCGGCGGCGGCGGCGGCGGCGGCGGCGGCAAGCGCCGCGACGCC

TCCGCCGACGGCGGCGGCGGCGGCGGCGACGAGGACATGACCATGGTGGCCACCGAGGTG

GTGCGCCTCAAGCAGGAGCAGCGCACCATCGACGACCGCGTGGCCGCCATGTGGCGCCGC

GTGCAGGAGACCGAGCGCCGCCCGAAGCAGATGCTCGCCTTCCTCCTCAAGGTGGTGGGC

GACCGCGACAAGCTCCACCGCCTCGTGGGCGGCGGCGGCAACGGCAACGGCGCCGCCACC

GCCGCCGCCGCCGACAACGGCTTCGCCGACGCCGCCCGCGCCGGCTGCGGCGAGAAGCGC

GCCCGCCTCCTCCTCGACGGCGACAACACCGGCGCCTTCGGCCCGGACGCCGTGGACTTC

GCCGGCTTCTACACCGGCGCCGACATGTTCCCGGACGTGGCCGTGGACGCCGCCGCCGCC

GCCGCCGGCGGCTCCGCCGGCTGCTCCTTCGCCTTCGGCGTGGACTCCGGCTACTGA

>LOC_Os06g36930

ATGGACTACTCCACCGTGAAGCAGGAGGAGGTGGAGGTGGTGGTGCTCGACGGCGAGGAG

GAGGCCGCCGCCGCCGCCGCCCCGGTGCCGCTCCCGGCCGCCATGGGCGTGGGCGCCGCC

GTGGCCCCGTTCCTCGTGAAGACCTTCGAGATGGTGGAGGACCCGGCCACCGACGCCGTG

GTGTCCTGGGGCGGCGCCGCCCGCAACTCCTTCGTGGTGTGGGACCCGCACGCCTTCGCC

GCCGGCCTCCTCCCGCTCCACTTCAAGCACGCCAACTTCTCCTCCTTCCTCCGCCAGCTC

AACACCTACGGCTTCCGCAAGGTGTCCGCCGACCGCTGGGAGTTCGCCAACGAGGACTTC

CTCGGCGGCCAGCGCCACCTCCTCGCCAACATCCGCCGCCGCCGCCGCGGCGCCGGCACC

GGCTCCACCACCCCGCGCGCCGTGAACTGCGGCGGCGGCGGCGGCGAGGGCGAGGTGGAG

CGCCTCCGCCGCGACAAGGAGGCCCTCGCCCGCGAGCTCGCCCGCCTCCGCCGCCAGCAG

CAGGAGGCCCGCGCCCAGCTCCTCGACATGGAGCGCCGCGTGCGCGGCACCGAGCGCCGC

CAGGAGCAGTGCACCGAGTTCCTCGCCCGCGCCCTCCGCTCCCCGGACGTGCTCGACAAC

ATCGCCCGCCGCCACGCCGCCGCCGTGGAGCGCAAGAAGCGCCGCATGCTCGCCGCCGCC

GCCGACGACGACGGCCTCACCTTCGAGGCCCTCGCCCTCGCCGCCGCCGCCGACACCTCC

CACTCCACCGGCGGCGCCGTGACCACCGACATGATCTGGTACGAGCTCCTCGGCGAGGAG

CAGGCCGAGATCGACATCGAGGTGGACCAGCTCGTGGCCTCCGCCTCCGCCGCCGCCGAC

ACCGCCTCCGAGGCCGAGCCGTGGGAGGAGATGGGCGAGGAGGAGGTGCAGGAGCTCGTG

CAGCAGATCGACTGCCTCGCCTCCCCGTCCTCCTGA

>LOC_Os07g08140

ATGGACGACCCGATGCTCAACGCCGTGAAGGAGGAGGAGTCCCACGGCGACGGCGGCGGC

CTCGAGGTGGTGGCCGGCGAGGACGGCGCCGCCGCCGTGGCCGCCGGCGTGGCCCCGCGC

CCGATGGAGGGCCTCCACGACGCCGGCCCGCCGCCGTTCCTCACCAAGACCTACGACATG

GTGGACGACGCCGGCACCGACGCCGCCGTGTCCTGGTCCGCCACCTCCAACTCCTTCGTG

GTGTGGGACCCGCACGCCTTCGCCACCGTGCTCCTCCCGCGCTTCTTCAAGCACAACAAC

TTCTCCTCCTTCGTGCGCCAGCTCAACACCTACGGCTTCCGCAAGGTGGACCCGGACCGC

TGGGAGTTCGCCAACGAGAACTTCCTCCGCGGCCAGCGCCACCTCCTCAAGAACATCAAG

CGCCGCAAGCCGCCGTCCCACACCGCCTCCAACCAGCAGTCCCTCGGCCCGTACCTCGAG

GTGGGCCACTTCGGCTACGACGCCGAGATCGACCGCCTCAAGCGCGACAAGCAGCTCCTC

ATGGCCGAGGTGGTGAAGCTCCGCCAGGAGCAGCAGAACACCAAGGCCAACCTCAAGGCC

ATGGAGGACCGCCTCCAGGGCACCGAGCAGCGCCAGCAGCAGATGATGGCCTTCCTCGCC

CGCGTGATGAAGAACCCGGAGTTCCTCAAGCAGCTCATGTCCCAGAACGAGATGCGCAAG

GAGCTCCAGGACGCCATCTCCAAGAAGCGCCGCCGCCGCATCGACCAGGGCCCGGAGGTG

GACGACGTGGGCACCTCCTCCTCCATCGAGCAGGAGTCCCCGGCCCTCTTCGACCCGCAG

GAGTCCGTGGAGTTCCTCATCGACGGCATCCCGTCCGACCTCGAGAACTCCGCCATGGAC

GCCGGCGGCCTCGTGGAGCCGCAGGACTTCGACGTGGGCGCCTCCGAGCAGCAGCAGATC

GGCCCGCAGGGCGAGCTCAACGACAACTTCTGGGAGGAGCTCCTCAACGAGGGCCTCGTG

GGCGAGGAGAACGACAACCCGGTGGTGGAGGACGACATGAACGTGCTCTCCGAGAAGATG

GGCTACCTCAACTCCAACGGCCCGACCGCCGGCGAGTGA

>LOC_Os07g44690

ATGGCCTTCCTCGTGGAGCGCTGCGGCGAGATGGTGGTGTCCATGGAGATGGGCCCGCAC

GGCGGCGGCGGCGCCGCCGCCGGCAAGCCGGTGCCGGCCCCGTTCCTCACCAAGACCTAC

CAGCTCGTGGACGACCCGTGCACCGACCACATCGTGTCCTGGGGCGAGGACGACACCACC

TTCGTGGTGTGGCGCCCGCCGGAGTTCGCCCGCGACCTCCTCCCGAACTACTTCAAGCAC

AACAACTTCTCCTCCTTCGTGCGCCAGCTCAACACCTACGGCTTCCGCAAGATCGTGGCC

GACCGCTGGGAGTTCGCCAACGAGTTCTTCCGCAAGGGCGCCAAGCACCTCCTCGCCGAG

ATCCACCGCCGCAAGTCCTCCCAGCCGCCGCCGCCGCCGATGCCGCACCAGCCGTACCAC

CACCACCACCACCTCAACCCGTTCTCCCTCCCGCCGCCGCCGCCGGCCTACCACCACCAC

CACCTCATCCAGGAGGAGCCGGCCACCACCGCCCACTGCACCGTGGCCGGCGACGGCGGC

GAGGGCGGCGACTTCCTCGCCGCCCTCTCCGAGGACAACCGCCAGCTCCGCCGCCGCAAC

TCCCTCCTCCTCTCCGAGCTCGCCCACATGAAGAAGCTCTACAACGACATCATCTACTTC

CTCCAGAACCACGTGGCCCCGGTGACCACCACCACCACCACCCCGTCCTCCACCGCCATG

GCCGCCGCCCAGCACCACCTCCCGGCCGCCGCCTCCTGCCGCCTCATGGAGCTCGACTCC

CCGGACCACTCCCCGCCGCCGCCGCCGCCGAAGACCCCGGCCACCGACGGCGGCGACACC

GTGAAGCTCTTCGGCGTGTCCCTCCACGGCCGCAAGAAGCGCGCCCACCGCGACGACGAC

GACGGCGTGCACGACCAGGGCTCCGAGGTGTGA

>LOC_Os08g36700

ATGGAGTGGGAGGAGGAGTCCGAGGCCGCCCGCCAGAAGGCCGCCGCCGCCTCCGCCTCC

GTGGTGCCGGCCCCGTTCCTCACCAAGACCTACCAGCTCGTGGACGACCCGGCCACCGAC

CACGTGGTGTCCTGGGAGGACGACGACGGCGGCGAGTCCGCCTCCTCCTTCGTGGTGTGG

CGCCCGCCGGAGTTCGCCCGCGACATCCTCCCGAACTACTTCAAGCACTCCAACTTCTCC

TCCTTCGTGCGCCAGCTCAACACCTACGGCTTCCGCAAGGTGGTGCCGGAGCGCTGGGAG

TTCGCCAACGAGTTCTTCCGCAAGGGCGAGAAGCAGCTCCTCTGCGAGATCCACCGCCGC

AAGTCCGCCGCCGCCACCTGGCCGCCGTTCCCGCCGCCGCCGCCGCCGTTCTTCGCCCCG

CGCCACTTCGCCGCCGGCGCCTTCTTCCGCCACGGCGACGGCATGCTCCACGGCCGCCTC

GGCGCCCTCGTGACCACCACCGAGCGCCGCCACTGGTTCGAGTCCGCCGCCCTCCCGGTG

GCCCCGTCCTCCCGCCTCCTCTCCCAGCTCGGCCCGGTGATCGCCCCGGCCCGCCGCGCC

GCCGCCACCCCGGAGGAGGAGGCCCTCATGCAGGAGAACCACCGCCTCCTCCGCGGCAAC

GCCGCCCTCGTGCAGGAGCTCGCCCACATGCGCAAGCTCTACTCCGACATCATCTACTTC

GTGCAGAACCACGTGCGCCCGGTGGCCCCGTCCCCGGCCGCCGCCGCCGCCCTCCACGGC

CTCGGCGTGCTCCGCCCGCCGCCGGCCGGCGGCAAGGCCCCGGCCTCCGAGGTGCGCGGC

GCCTCCGGCCGCTCCGCCACCTCCTCCTCCTCCCTCACCGTGGCCGAGGACCAGCCGACC

CTCCTCGCCCTCCGCCTCCCGCGCACCACCGAGAAGATCATCAACGAGGTGTCCGGCGGC

AACGGCGGCGGCTCCACCAAGCTCTTCGGCGTGCACCTCTCCTCCGCCGACGAGCAGACC

TCCTCCGGCGCCTCCCGCAAGCGCTCCCCGCCGCAGGAGCAGCCGCCGACCTCCCCGGCC

CCGAAGCGCACCCTCGTGGTGGAGCACTCCGAGCTCCGCCTCTCCATCGTGTCCCCGCCG

TGA

>LOC_Os08g43334

ATGAAGGGCTCCCGCCTCGCCGTGAAGGAGTCCTGCCTCCCGATGACCATGCCGATGCCG

GAGACCTTCGCCCAGTACTCCAACCCGCTCCGCTCCACCCGCGCCTACGGCCAGCTCTGC

CGCGTGCCGGGCCGCCGCCGCCACGCCTGCGTGGACGGCTGGGGCCAGGACCGCGCCCGC

ACCCGCGGCGACGACGGCCAGCGCCGCTACGCCCAGTGCCGCGGCGGCTGGGGCGACATC

TGGCGCTGGGGCGGCTACATCACCTGCGGCTCCCCGCGCGAGACCGGCATGGTGAACGGC

GCCCCGTCCCCGCCGCCGCCGTCCCCGATGGTGATGTCCTTCGGCCCGCTCGACTCCCCG

TGGGTGAAGCAGCCGGACACCACCGTGTACCCGGGCCAGATCTGCGCCGCCGCCGGCGGC

GGCGGCGGCATGGCCGACCAGACCGCCGCCGCCGTGGTGGTGGGCGGCGGCGCCGCCGCC

ACCATGGGCGAGCCGTCCCCGCCGCCGCCGGCCCCGGCCGCCGAGGCCGCCGGCGTGGGC

GTGGGCCAGCAGCAGCGCACCGTGCCGACCCCGTTCCTCACCAAGACCTACCAGCTCGTG

GACGACCCGGCCGTGGACGACGTGATCTCCTGGAACGACGACGGCTCCACCTTCGTGGTG

TGGCGCCCGGCCGAGTTCGCCCGCGACCTCCTCCCGAAGTACTTCAAGCACAACAACTTC

TCCTCCTTCGTGCGCCAGCTCAACACCTACGGCTTCCGCAAGATCGTGCCGGACCGCTGG

GAGTTCGCCAACGACTGCTTCCGCCGCGGCGAGCGCCGCCTCCTCTGCGAGATCCACCGC

CGCAAGGTGACCCCGCCGGCCCCGGCCGCCACCACCGCCGCCGTGGCCGCCGCCATCCCG

ATGGCCCTCCCGGTGACCACCACCCGCGACGGCTCCCCGGTGCTCTCCGGCGAGGAGCAG

GTGATCTCCTCCTCCTCCTCCCCGGAGCCGCCGCTCGTGCTCCCGCAGGCCCCGTCCGGC

TCCGGCTCCGGCGGCGTGGCCTCCGGCGACGTGGGCGACGAGAACGAGCGCCTCCGCCGC

GAGAACGCCCAGCTCGCCCGCGAGCTCTCCCAGATGCGCAAGCTCTGCAACAACATCCTC

CTCCTCATGTCCAAGTACGCCTCCACCCAGCAGCTCGACGCCGCCAACGCCTCCTCCGCC

GCCGGCAACAACAACAACAACAACTGCTCCGGCGAGTCCGCCGAGGCCGCCACCCCGCTC

CCGCTCCCGGCCGTGCTCGACCTCATGCCGTCCTGCCCGGGCGCCGCCTCCGCCGCCGCC

CCGGTGTCCGACAACGAGGAGGGCATGATGTCCGCCAAGCTCTTCGGCGTGTCCATCGGC

CGCAAGCGCATGCGCCACGACGGCGGCGGCGACGACGACCACGCCGCCACCGTGAAGGCC

GAGCCGATGGACGGCCGCCCGCACGGCAAGGACGAGCAGTCCGCCGAGACCCAGGCCTGG

CCGATCTACCGCCCGCGCCCGGTGTACCAGCCGATCCGCGCCTGCAACGGCTACGAGTAC

GACCGCGCCGGCTCCGACCAGGACGAGTGCGGCGCCAAGCCGTCCAACCAGGACAAGAAG

GACCTCCTCCGCCGCTGGGTGGTGCTCATCTCCGAGTCCCTCCAGCTCCACGGCCGCCAG

GTGGTGGGCGCCGTGCAGGAGTGGCGCCGCGACCGCCGCGGCCACCGCCGCCCGCCGGCC

GGCCACGTGCAGCTCCGCACCTGCGTGAACAACCTCCTCGTGACCGGCGCCGGCACCCTC

GACGCCGGCCGCGAGTACGAGACCAACTCCCTCCCGCGCCCGCGCCGCTGA

>LOC_Os09g28200

ATGGAGCGCTGCGGCTCCTGGTCCGACTGCGAGGCCGCCGCCGCCGCCGCCCAGAAGGCC

GTGCCGGCCCCGTTCCTCACCAAGACCTACCAGCTCGTGGACGACCCGGCCACCGACCAC

ATCGTGTCCTGGGGCGACGACCGCGTGTCCACCTTCGTGGTGTGGCGCCCGCCGGAGTTC

GCCCGCGACATCCTCCCGAACTACTTCAAGCACAACAACTTCTCCTCCTTCGTGCGCCAG

CTCAACACCTACGGCTTCCGCAAGGTGGTGCCGGAGCGCTGGGAGTTCGCCAACGAGTTC

TTCCGCAAGGGCGAGAAGCAGCTCCTCACCGAGATCCACCGCCGCAAGACCTCCTCCGCC

TCCACCGCCTCCCCGTCCCCGCCGCCGTTCTTCGCCCCGCCGCACTTCCCGCTCTTCCAC

CACCCGGGCGTGGCCGCCGCCCAGCACCACCACGCCTTCGTGGGCGACGACGGCGTGGTG

GCCGCCCACGGCATCGGCATGCCGTTCCCGCAGCCGCACTGGCGCGAGCCGAACCTCCCG

GTGGCCACCCGCCTCCTCGCCCTCGGCGGCCCGGCCCCGTCCCCGTCCTCCGCCGAGGCC

GGCGGCGCCGGCCGCGCCGCCACCGCCGCCGTGCTCATGGAGGAGAACGAGCGCCTCCGC

CGCTCCAACACCGCCCTCCTCCAGGAGCTCGCCCACATGCGCAAGCTCTACAACGACATC

ATCTACTTCGTGCAGAACCACGTGCGCCCGGTGGCCCCGTCCCCGGCCGCCGCCGCCTTC

CTCCAGGGCCTCGGCATGCAGGCCCGCAAGAAGCCGGCCGCCGCCAACGTGCTCAACAAC

TCCGGCGGCTCCACCACCTCCTCCTCCTCCCTCACCATCGCCGAGGAGCCGTCCCCGCCG

CCGCAGCAGCAGCACCTCGCCGGCGAGAAGTCCGGCGGCGAGGCCGGCAACTCCTCCGCC

GCCCGCTCCTCCGCCCCGACCAAGCTCTTCGGCGTGCACCTCTCCGCCGCCCCGTGCGGC

GCCGGCTCCAAGCGCGCCTCCTCCCCGGAGGAGCACCCGCCGACCTCCCCGGCCACCAAG

CCGCGCCTCGTGCTCGAGTGCGACGACCTCTCCCTCACCGTGGCCCCGTCCTCCTCCTCC

CAGCAGCAGCTCTCCGCCGCCTCCTCCCCGACCTCCACCTCCTGA

>LOC_Os09g28354

ATGGCCGCCGCCGAGGCCGCCGCCGCCGTGGGCAAGCAGCAGCAGAAGGGCGGCGGCGGC

CGCGGCGGCGGCGGCGGCGGCCCGGCCCCGTTCCTCACCAAGACCAACCAGATGGTGGAG

GAGTCCGCCACCGACGAGGTGATCTCCTGGGGCAAGGAGGGCCGCTCCTTCGTGGTGTGG

AAGCCGGTGGAGTTCGCCCGCGACCTCCTCCCGCTCCACTTCAAGCACTGCAACTTCTCC

TCCTTCGTGCGCCAGCTCAACACCTACGGCTTCCGCAAGGTGGTGCCGGACCGCTGGGAG

TTCGCCAACGGCAACTTCCGCCGCGGCGAGCAGGGCCTCCTCTCCGGCATCCGCCGCCGC

AAGGCCACCACCCCGCAGTCCTCCAAGTCCTGCGGCTCCGGCGTGAACGTGGCCTTCCCG

CCGCCGCTCCCGCCGCTCCCGCCGGAGCCGTCCGCCACCACCTCCTCCGGCAACGACCGC

TCCTCCTCCTCCGCCTCCTCCCCGCCGCGCGCCGACATCACCTCCGAGAACGAGCAGCTC

CGCAAGGACAACCAGACCCTCACCATGGAGCTCGCCCGCGCCCGCCGCCACTGCGAGGAG

CTCCTCGGCTTCCTCTCCCGCTTCCTCGACGTGCGCCAGCTCGACCTCCGCCTCCTCATG

CAGGAGGACATGCGCGCCGCCGCCGGCGGCGTGGGCGGCGAGCAGCGCGTGCAGGAGCAC

GCCCGCGAGGAGAAGTGCGTGAAGCTCTTCGGCGTGCTCCTCGACGACACCCACGGCGCC

GCCACCCGCAAGCGCGCCCGCTGCGAGGAGGCCGCCGCCTCCGAGCGCCCGATCAAGATG

ATCCGCATCGGCGAGCCGTGGGTGTCCGTGCCGTCCTCCGGCCCGGCCCGCTGCGGCGGC

GACAACTGA

>LOC_Os09g35790

ATGGCCGAGCAGGGCGCCGGCGAGGCCGACGCCGGCGGCGGCGAGCCGCCGCCGGCCGCC

GTGATGACCGCCGCCGCCGAGGCCCTCGCCGGCCAGCGCTCCCTCCCGACCCCGTTCCTC

ACCAAGACCTACCAGCTCGTGGAGGACCCGGCCGTGGACGACGTGATCTCCTGGAACGAG

GACGGCTCCACCTTCGTGGTGTGGCGCCCGGCCGAGTTCGCCCGCGACCTCCTCCCGAAG

TACTTCAAGCACAACAACTTCTCCTCCTTCGTGCGCCAGCTCAACACCTACGGCTTCCGC

AAGATCGTGCCGGACCGCTGGGAGTTCGCCAACGACTGCTTCCGCCGCGGCGAGAAGCGC

CTCCTCTGCGACATCCACCGCCGCAAGGTGGTGGCCGCCGCCGCCGCCGCCCCGCCGCCG

CCGTCCCCGGGCATGGCCACCGCCGCCGCCGCCGTGGCCTCCGGCGCCGTGACCGTGGCC

GCCGCCCCGATCCCGATGGCCCTCCCGGTGACCCGCGCCGGCTCCCCGGCCCACTCCTCC

GAGGAGCAGGTGCTCTCCTCCAACTCCGGCTCCGGCGAGGAGCACCGCCAGGCCTCCGGC

TCCGGCTCCGCCCCGGGCGGCGGCGGCGGCGGCTCCGCCTCCGGCGGCGACATGGGCGAG

GAGAACGAGCGCCTCCGCCGCGAGAACGCCCGCCTCACCCGCGAGCTCGGCCACATGAAG

AAGCTCTGCAACAACATCCTCCTCCTCATGTCCAAGTACGCCGCCACCCAGCACGTGGAG

GGCTCCGCCGGCATCTCCTCCATCGCCAACTGCTCCGGCGAGTCCTCCGAGGCCGTGCCG

CCGCCGCCGCCGCTCCCGCCGGCCATCCTCGACCTCATGCCGTCCTGCCCGGCCCTCGCC

ACCGCCGCCGCCGCCGCCGGCCTCGCCATCGACGGCGAGCCGGACCCGTCCGCCCGCCTC

TTCGGCGTGTCCATCGGCCTCAAGCGCACCCGCGACGACGCCGCCGCCGCCGCCGACGAG

GACGGCGGCGGCGAGGACCAGGCCGAGCACGGCGGCGCCGACGTGAAGCCGGAGGCCGCC

GACCCGCACCCGGCCGGCGGCGGCGGCGGCTCCTCCACCGAGGCCTCCCCGGAGTCCCAC

CCGTGGCCGATCTACCGCCCGACCCCGATGTACCACGCCGTGCGCCCGACCTGCAACGGC

CCGGACCGCGCCGGCTCCGACCAGGACGGCTCCTCCTCCTCCCAGACCATGGGCCCGGGC

GAGTTCGACGACCTCCAGAAGATGATGGTGGTGCAGCAGTCCAACTTCGTGATGCACTGG

GGCCGCTCCGAGTGCGGCTCCGGCGTGCGCGGCTTCGGCTGGTGA

>LOC_Os10g28340

ATGGACCCGGCCGCCGCCGGCATCGTGAAGGAGGAGATGCTCGAGTCCCAGCAGCAGCAG

CGCCAGGAGGACGGCGGCGCCGCCCCGCGCCCGATGGAGGGCCTCCACGAGGTGGGCCCG

CCGCCGTTCCTCACCAAGACCTACGACCTCGTGGAGGACCCGGCCACCGACGGCGTGGTG

TCCTGGTCCCGCGCCGGCAACTCCTTCGTGGTGTGGGACCCGCACGTGTTCGCCGACCTC

CTCCTCCCGCGCCTCTTCAAGCACAACAACTTCTCCTCCTTCGTGCGCCAGCTCAACACC

TACGGCTTCCGCAAGGTGGACCCGGACCGCTGGGAGTTCGCCAACGAGGGCTTCCTCCGC

GGCCAGCGCCACCTCCTCAAGACCATCAAGCGCCGCAAGCCGCCGTCCAACGCCCCGCCG

TCCCAGCAGCAGTCCCTCACCTCCTGCCTCGAGGTGGGCGAGTTCGGCTTCGAGGAGGAG

ATCGACCGCCTCAAGCGCGACAAGAACATCCTCATCACCGAGGTGGTGAAGCTCCGCCAG

GAGCAGCAGGCCACCAAGGACCACGTGAAGGCCATGGAGGACCGCCTCCGCGCCGCCGAG

CAGAAGCAGGTGCAGATGATGGGCTTCCTCGCCCGCGCCATGCGCAACCCGGAGTTCTTC

CAGCAGCTCGCCCAGCAGAAGGAGAAGCGCAAGGAGCTCGAGGACGCCATCTCCAAGAAG

CGCCGCCGCCCGATCGACAACGTGCCGTTCTACGACCCGGGCGAGACCTCCCAGACCGAG

CAGCTCGACTCCCCGTACCTCTTCGACTCCGGCGTGCTCAACGAGCTCTCCGAGCCGGGC

ATCCCGGAGCTCGAGAACCTCGCCGTGAACATCCAGGACCTCGGCAAGGGCAAGGTGGAC

GAGGAGCGCCAGAACCAGACCAACGGCCAGGCCGAGCTCGGCGACGACTTCTGGGCCGAG

CTCCTCGTGGAGGACTTCACCGGCAAGGAGGAGCAGTCCGAGCTCGACGGCAAGATCGAC

GGCATCGACGAGCTCGCCCAGCAGCTCGGCTACCTCTCCTCCACCTCCCCGAAGTGA
