# Supplementary material for: In-silico analysis of heat shock transcription factor (OsHSF) gene family in rice (Oryza sativa L.)
Source: BMC Plant Biol. 2023 Aug 17;23:395. doi: 10.1186/s12870-023-04399-1 (PMC10433574; doi:10.1186/s12870-023-04399-1)
Supplement: Supplementary file 3 — Additional file 3. [file 12870_2023_4399_MOESM3_ESM.docx]

**S3.**

A total of 15 consensus sequences of HSF family motifs.

**Motif-1- ACTTCAAGCACWMCAACTTCTCCTCCTTCGTGCGCCAGCTCAACACCTAC**

**
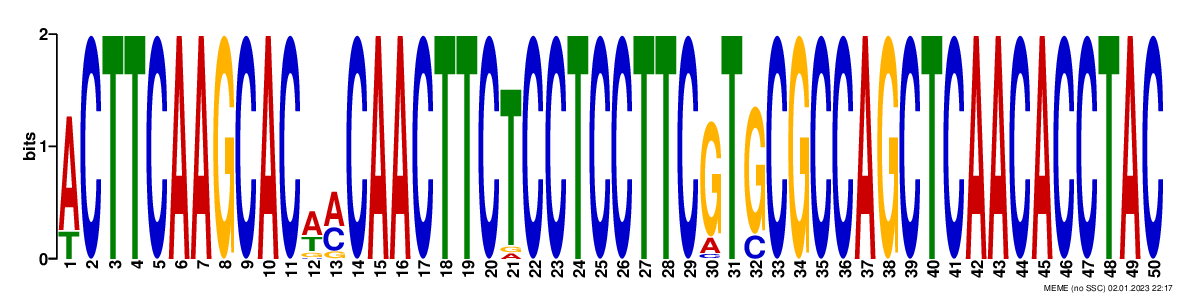
**

**Motif 2- GGCTTCCGCAAGGTGGWCCCGGACCGCTGGGAGTTCGCCAACGAGKVCTT**

**
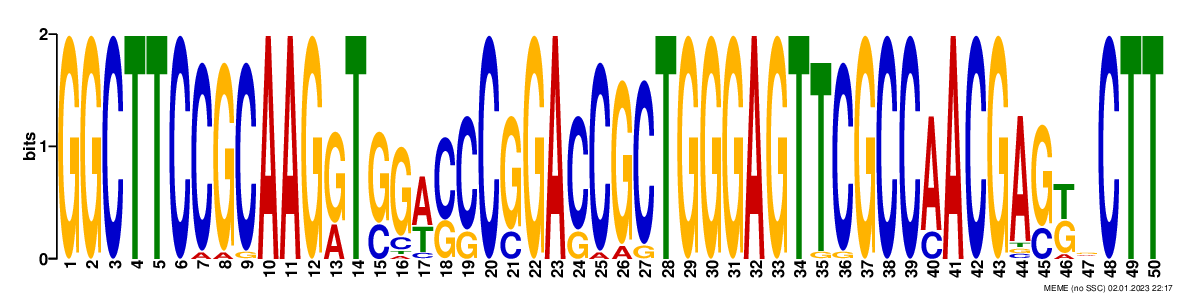
**

**Motif 3- CSCCGTTCCTCACCAAGACCTACSASMTSGTGGACGACCCGGCCACCGAC**

**
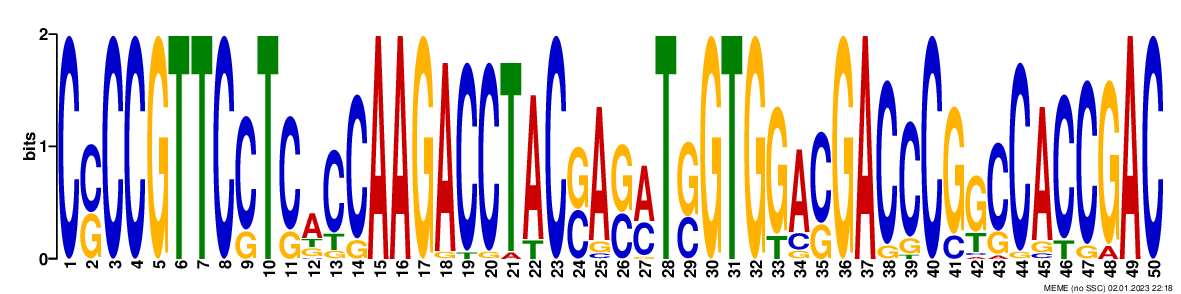
**

**Motif 4- MCTCCTTCGTGGTGTGGSACCCGCMCGMSTTCGCCCGCGWCCTCCTCCCG**

**
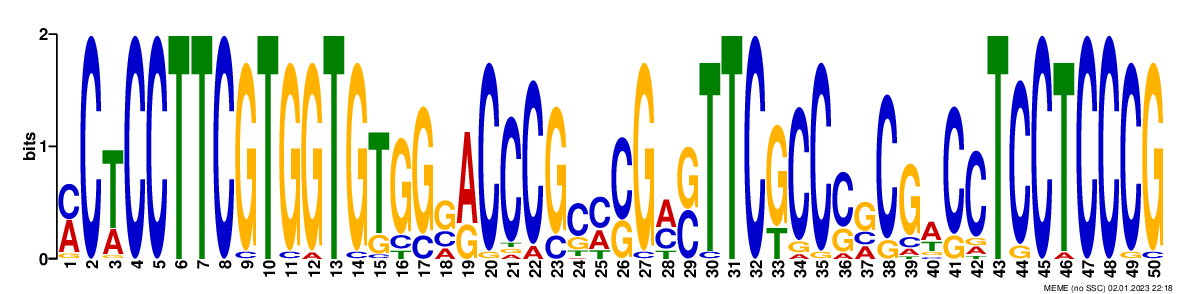
**

**Motif 5- CCTCCGCGGCSAGAASCACCTCCTCAVSRACATCMASCGCCGCAAGBCS**

**
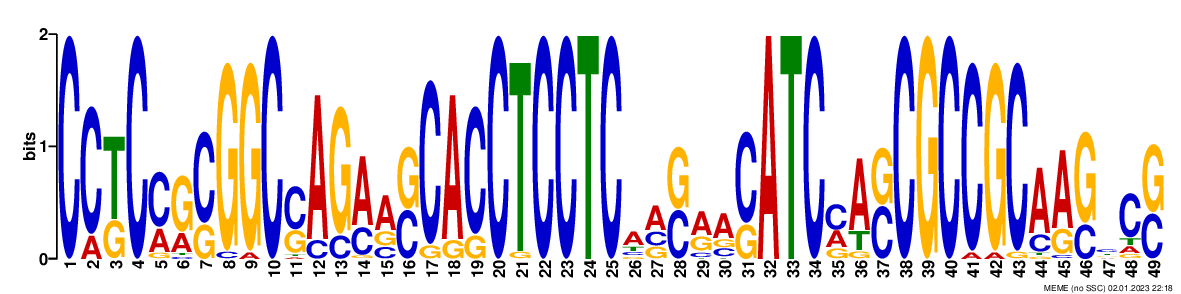
**

**Motif 6- AGGAGAWCGASCGCCTCCRSCGCGASAASNCCSYCCTCVTCSMSGAGSTS**

**
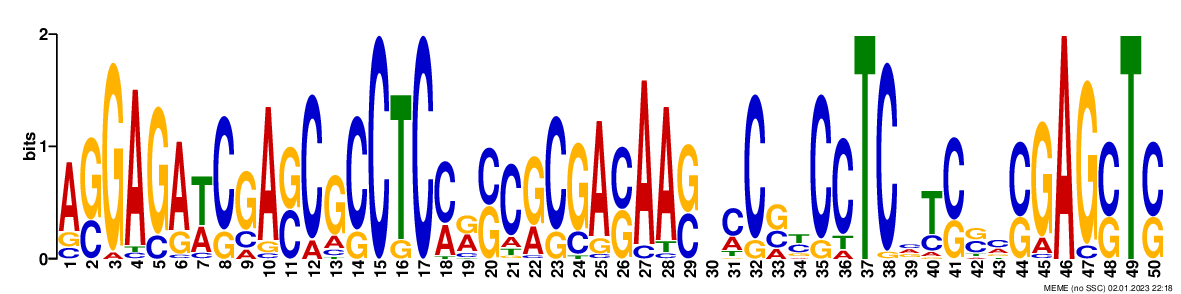
**

**Motif 7- SVCRYSGAGCASCDCCASVASSASATSATSKCCTTCCTCSCCMRSSHCVT**

**
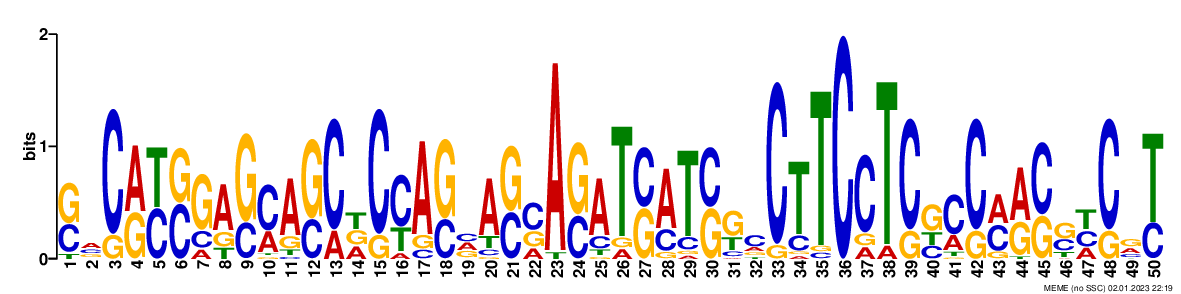
**

**Motif 8- TCCGCCASSASCAGCASRMCACSAAGSNCSASVTSSAGGMCMTSGAGGAS**

**
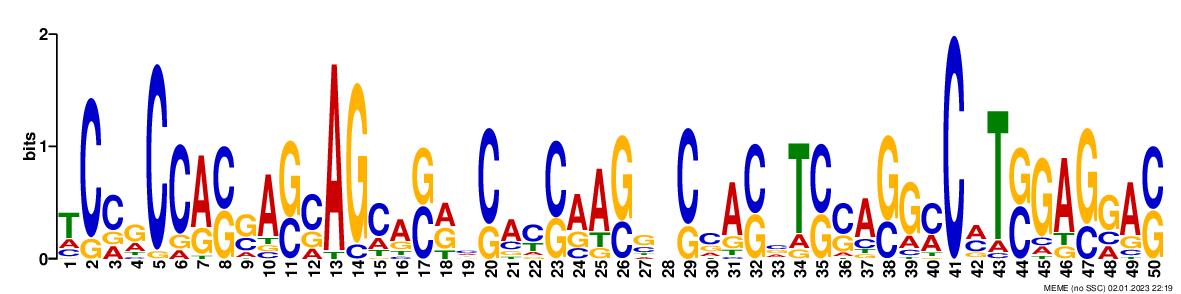
**

**Motif 9- MCGACVYSSACSHSYTCDCCSASVASRWSGRCBHCCYCWCCTCCDCCKMC**

**
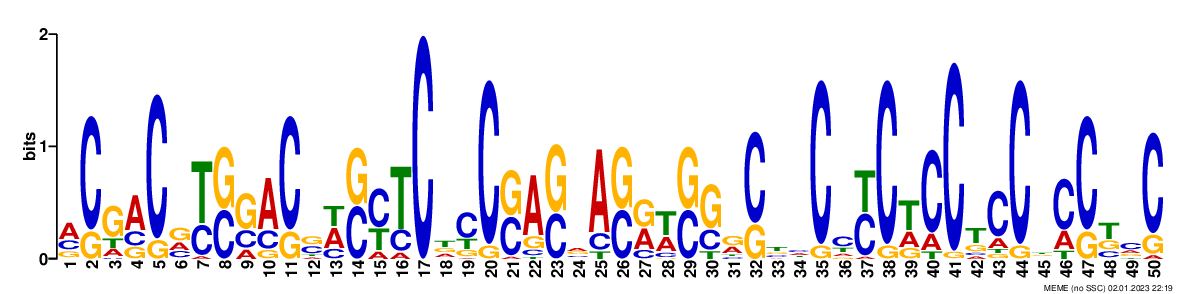
**

**Motif 10- CGMSVHCRACGACGRCKKCKGSGWGSAGCTCCTCRVCGWS**

**
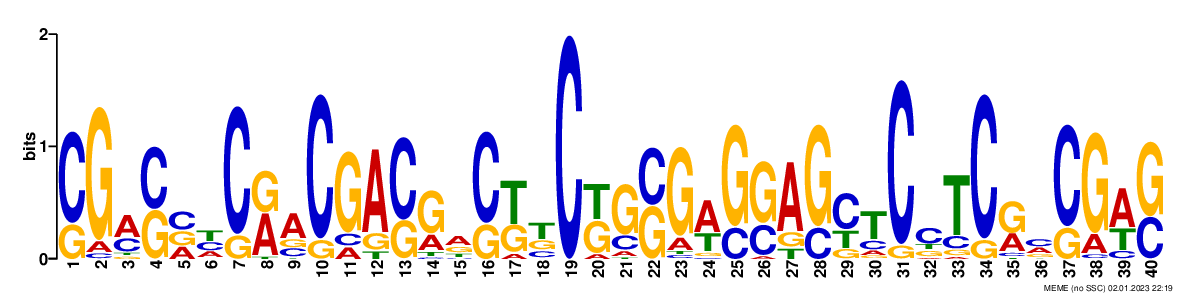
**

**Motif 11- VCGTGRTSTCCTGGGGCSVSG**

**
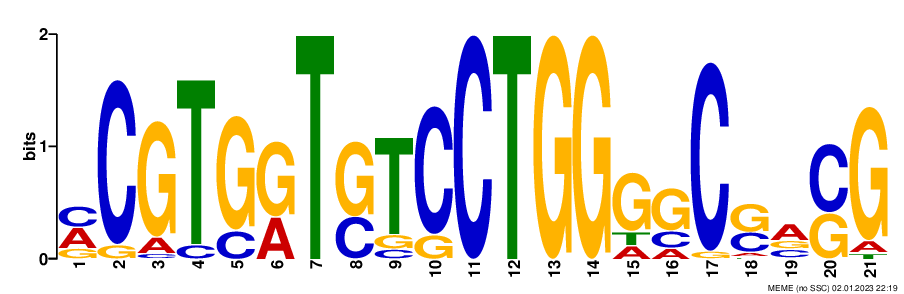
**

**Motif 12- CMGAACCCGGMSTYCCTSCASCAGCTCDYSCMSCASCMCSA**

**
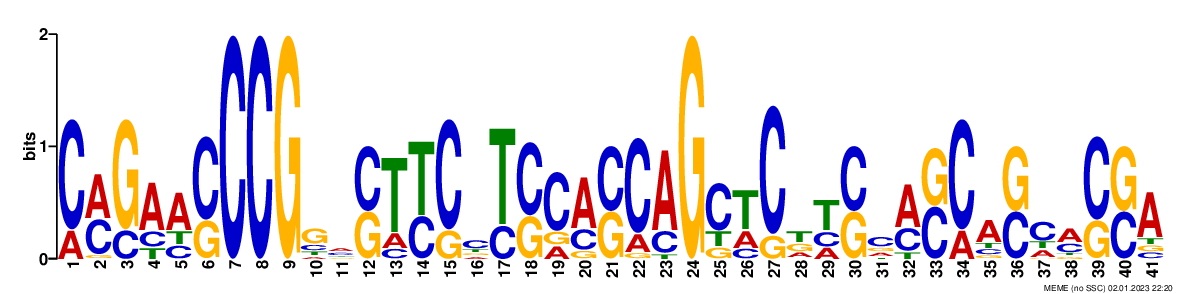
**

**Motif 13- CGVSGMSGCCGCCGCCGCCGCCGCCSYCG**

**
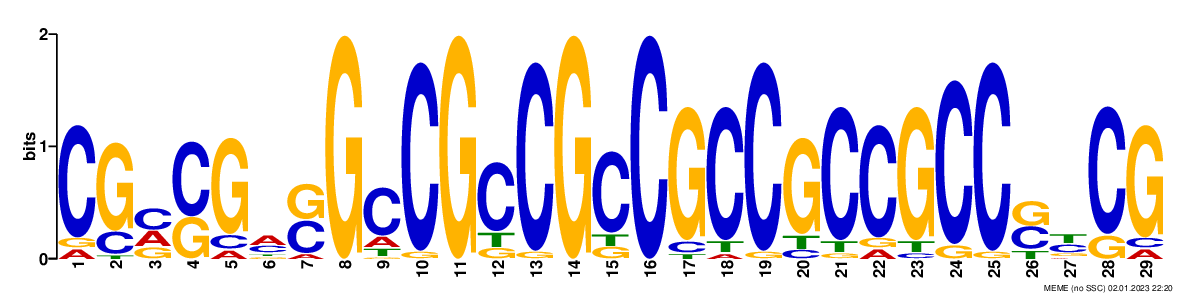
**

**Motif 14- CCNYCBCCVNSRAGCGCCGCCKCSBSRTSGASGVCGNCBMCVWCSDCGAC**

**
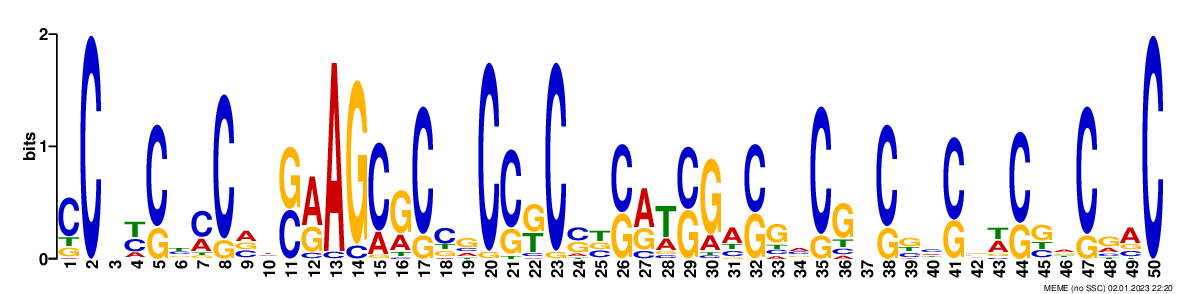
**

**Motif 15- YSCCGKMCSTSGCCRCSKCCTCCGCCSYCNMCKCCWCCGGC**

**
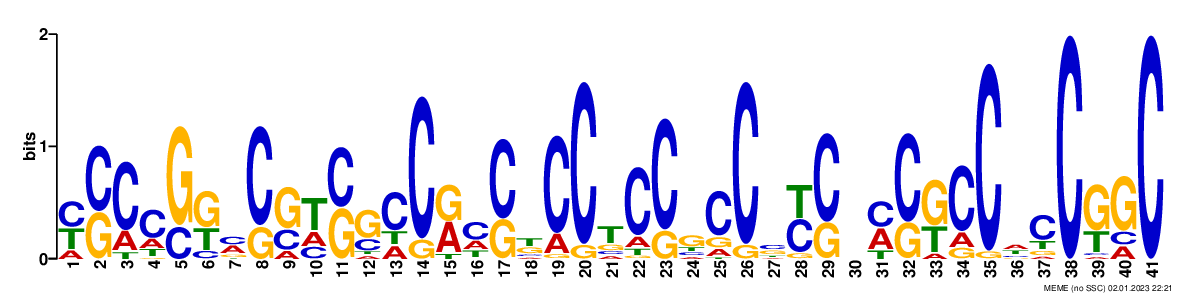
**
